# Endoscopic treatments for Barrett's esophagus: a systematic review of safety and effectiveness compared to esophagectomy

**DOI:** 10.1186/1471-230X-10-111

**Published:** 2010-09-27

**Authors:** Devidas Menon, Tania Stafinski, Heng Wu, Darren Lau, Clarence Wong

**Affiliations:** 1 Department of Public Health Sciences, University of Alberta, Room 3021, Research Transition Facility, 8308 114 Street, Edmonton, Alberta, T6G 2V2, Canada; 2 Department of Public Health Sciences, University of Alberta, 2-040 Health Research Innovation Facility East, 8602 112 Street, Edmonton, Alberta, T6G 2E1, Canada; 3 Division of Gastroenterology, University of Alberta, 331 Community Services Centre, Royal Alexandra Hospital, Edmonton, Alberta, T5H 3V9, Canada

## Abstract

**Background:**

Recently, several new endoscopic treatments have been used to treat patients with Barrett's esophagus with high grade dysplasia. This systematic review aimed to determine the safety and effectiveness of these treatments compared with esophagectomy.

**Methods:**

A comprehensive literature search was undertaken to identify studies of endoscopic treatments for Barrett's esophagus or early stage esophageal cancer. Information from the selected studies was extracted by two independent reviewers. Study quality was assessed and information was tabulated to identify trends or patterns. Results were pooled across studies for each outcome. Safety (occurrence of adverse events) and effectiveness (complete eradication of dysplasia) were compared across different treatments.

**Results:**

The 101 studies that met the selection criteria included 8 endoscopic techniques and esophagectomy; only 12 were comparative studies. The quality of evidence was generally low. Methods and outcomes were inconsistently reported. Protocols, outcomes measured, follow-up times and numbers of treatment sessions varied, making it difficult to calculate pooled estimates.

The surgical mortality rate was 1.2%, compared to 0.04% in 2831 patients treated endoscopically (1 death). Adverse events were more severe and frequent with esophagectomy, and included anastomotic leaks (9.4%), wound infections (4.1%) and pulmonary complications (4.1%). Four patients (0.1%) treated endoscopically experienced bleeding requiring transfusions. The stricture rate with esophagectomy (5.3%) was lower than with porfimer sodium photodynamic therapy (18.5%), but higher than aminolevulinic acid (ALA) 60 mg/kg PDT (1.4%). Dysphagia and odynophagia varied in frequency across modalities, with the highest rates reported for multipolar electrocoagulation (MPEC). Photosensitivity, an adverse event that occurs only with photodynamic therapy, was experienced by 26.4% of patients who received porfimer sodium.

Some radiofrequency ablation (RFA) or argon plasma coagulation (APC) studies (used in multiple sessions) reported rates of almost 100% for complete eradication of dysplasia. But the study methods and findings were not adequately described. The other studies of endoscopic treatments reported similarly high rates of complete eradication.

**Conclusions:**

Endoscopic treatments offer safe and effective alternatives to esophagectomy for patients with Barrett's esophagus and high grade dysplasia. Unfortunately, shortcomings in the published studies make it impossible to determine the comparative effectiveness of each of the endoscopic treatments.

## Background

Barrett's esophagus (BE) is a benign condition where abnormal cells (intestinal metaplasia), replace the normal lining of the esophagus. It is typically caused by long-term gastroesophageal reflux disease. Between 2% to 6% of Canadians may have Barrett's esophagus[1]. Similar prevalence rates have been reported in studies from Sweden (1.6%) and the United States (5.9%)[2,3].

Although Barrett's esophagus itself is not harmful, in some individuals, precancerous dysplasia develops in the Barrett's tissue. The presence of dysplasia carries a higher risk of developing esophageal adenocarcinoma - a type of esophageal cancer. In addition to the cancer risk, Barrett's esophagus decreases patients' quality of life and increases health care costs[4-7].

The rising incidence of esophageal adenocarcinoma has focused attention on preventing cancer by removing the dysplasia and allowing normal, squamous esophageal mucosa to regenerate. Endoscopic techniques have been developed as a result. They can be applied sequentially to increase diagnostic yield and improve treatment outcomes. There are two categories: endoscopic mucosal resection (EMR) and endoscopic ablation. EMR with a cautery snare excision technique can remove visible raised or flat lesions for diagnostic and therapeutic roles. Diagnostically, it allows for complete histopathological assessment of the target mucosa. Those with superficial lesions can go on to further ablative techniques. Lesions that are found to invade submucosa may need referral for surgical resection. Therapeutically, EMR can be used for curative intent if the target lesion is small. However, in most cases of Barrett's esophagus, it is used to remove dysplastic nodules leaving the larger surface area for endoscopic ablation.

Photodynamic therapy (PDT) is one of the new endoscopic treatments used to remove dysplasia. Other endoscopic treatments include: argon plasma coagulation (APC), cryoablation, laser ablation, multipolar electrocoagulation (MPEC), radiofrequency ablation (RFA), and thermocoagulation. Depending on the extent of dysplasia, several endoscopic treatment sessions or a combination of treatments may be used.

Patients also receive long-term drug therapy to control gastroesophageal reflux and prevent further damage to the esophagus[8]. Endoscopic therapies are less invasive alternatives to esophagectomy (surgical removal of the esophagus), which is associated with high rates of morbidity and mortality, and with decreased quality of life[9-11].

Clinicians now have a variety of technologies to choose from when treating Barrett's esophagus with dysplasia. This systematic review of published clinical studies compares the evidence on the safety and effectiveness of the endoscopic treatments and of esophagectomy and may provide some guidance for clinical practice.

## Methods

### Data collection

#### Literature search

An extensive search for published and unpublished studies of endoscopic and non-endoscopic procedures for Barrett's esophagus was performed. Search terms included keywords and controlled vocabulary terms used to describe Barrett's, photodynamic therapy and other endoscopic techniques, and esophagectomy. Searches for the alternative treatments (i.e., other than PDT) were limited to studies from 2003 to January 2009. The bibliographic databases searched included: PubMed (MEDLINE), The Cochrane Library, the UK Centre for Reviews and Dissemination (DARE, Health Technology Assessment, and NHS Economic Evaluation) databases, EMBASE, CINAHL, Web of Science and EconLit. Monthly update searches in PubMed were run throughout the project to identify new studies. Meeting abstracts from the American Society of Clinical Oncology and Digestive Disease Week, as well as practice guidelines and clinical trials web sites were also searched, as were the reference lists from relevant papers and earlier health technology assessments.

#### Study selection

Results from the literature searches were imported into a Reference Manager^® ^database to remove duplicates and manage bibliographic citations. Titles and abstracts (where available), were independently screened by two researchers. The full papers of potentially relevant studies were retrieved and assessed against pre-defined inclusion criteria (Table 1). Non-English language studies were excluded, unless an English language abstract provided sufficient detail on patients and outcomes.

**Table 1:** Criteria for including studies in this review

Parameter	Inclusion criteria	Exclusion criteria
	• Randomized or controlled (e.g., pseudo-randomized or quasi-randomized) trials	
*Study design*	• Non-randomized clinical trials	• Editorials & opinion pieces
	• Retrospective, prospective, or concurrent cohort studies	• Review articles
	• Case or clinical series	
*Participants*	• Patients diagnosed with BE	• Patients diagnosed with esophageal cancer or other conditions
	• PDT	
*Interventions*	• Esophagectomy	
	• EMR	
	• Other endoscopic treatments (cryoablation, laser ablation, APC, MPEC, RFA, thermocoagulation)	
*Comparators*	• Same as interventions above	
*Outcomes*	• Not used for inclusion/exclusion due to heterogeneity of data	

#### Critical appraisal and synthesis

Information from the studies was extracted by two reviewers using a pre-tested data abstraction form and a set of decision rules. The form contained elements for examining the purpose and methods of each study (Table 2). Missing data were sought from study authors. Consensus between reviewers on the information collected was assessed using the Kappa statistic.

**Table 2:** Summary of elements in the data abstraction form

Parameter	Description of information collected
*Cancer/cell type*	BE; dysplasia
*Study design*	Setting; study type; treatment(s) used; length of follow-up
*Patients*	Number of patients by treatment group; age; gender; length of Barrett's; inclusion*/*exclusion criteria; prior treatments
*Intervention*	Details of the treatment; number of patients who underwent each treatment; co-interventions
*Outcomes*	Complete and partial response; survival; recurrence; progression to cancer; reduction in length of Barrett's; adverse events

The quality of each study was also assessed by two reviewers using the Oxford Centre for Evidence-based Medicine Levels of Evidence[12]. Discrepancies were resolved through consensus and Kappa scores were calculated.

### Data analysis

#### Qualitative

Information was summarized in tabular form to more easily identify trends or patterns in findings reported across studies.

#### Quantitative

Results from individual studies were pooled using weighted mean values to generate summary estimates for each of the outcomes of interest. All quantitative analyses were conducted in accordance with intention-to-treat principles (i.e., patients were analyzed in the groups to which they were originally allocated, regardless of whether or not they received the assigned treatment).

## Results

### Description of studies

**Figure 1:**
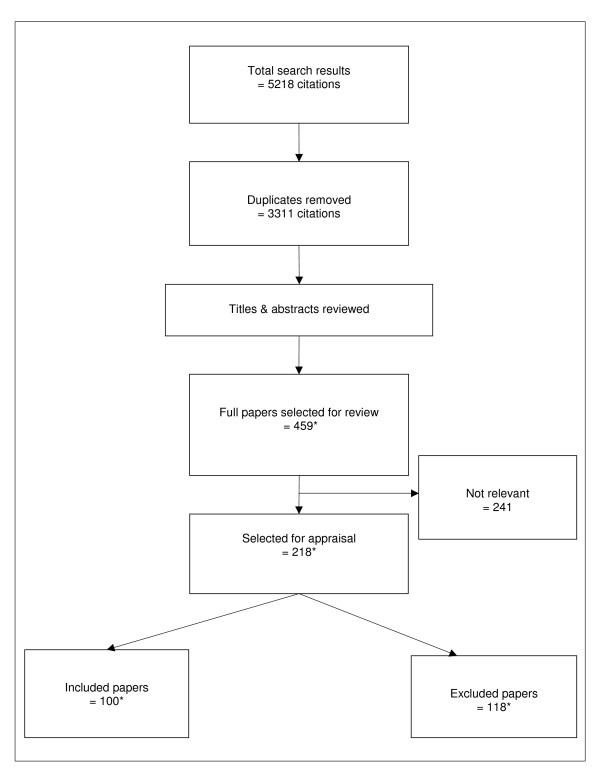
**Flow diagram of study selection**. Notes: *10 new studies were added since the first literature review. Of these studies, 6 were included, and 4 were excluded.

Over 400 potentially relevant papers were selected from the literature search results and reviewed for inclusion (Figure 1). Of these, 99 papers, reporting on 101 separate studies and 3042 patients, met the inclusion criteria. Descriptions of each study are presented in Additional files 1, 2, 3, 4, 5, 6, 7, and 8. A breakdown of studies by the type of intervention and study design is presented in Table 3.

**Table 3:** Numbers of included studies and patients

	Treatment	Number of studies	Comparative studies	Non-comparative studies	Number of patients
**Endoscopic**	PDT	37	5[13-16,25]	32[22,26-54]	1028
	APC	26	7[13-18,55]	19[23,56-73]	792
	Cryoablation	2	0	2[74,75]	31
	Combined EMR & PDT	2	1[25]	1[76]	6
	Thermocoagulation	1	0	1[77]	13
	EMR	6	2[20,25]	4[78-81]	38
	Laser ablation	7	0	7[24,82-87]	91
	MPEC	6	2[17,18]	4[88-90]	118
	RFA	16	2[19,92]	14[93-105]	714
	Total*	95*	11*	86	2831
**Surgical**	Esophagectomy	8	3[21,57,79]	5[109-111]112	211

Total*		101*	12*	91	3042

***Notes: ***(1) APC (argon plasma coagulation), EMR (endoscopic mucosal resection), MPEC (multipolar electrocoagulation), PDT (photodynamic therapy), RFA (radiofrequency ablation) (2) *9 comparative studies are reported in both intervention groups (e.g., APC and PDT), thus the number of separate comparative trials is less than the sum of entries in the column.

### Quality of studies

The quality of the evidence reviewed was generally low. Only 12 studies were comparative (Table 3), and of these, 5 were cohort studies with uncontrolled allocation of patients to each treatment group. Details of the study methods used were sparse, with missing information or inconsistent reporting of outcomes across patient groups.

Seven randomized controlled trials (RCTs) were identified, but these compared only APC with PDT (4 trials[13-16]), or APC with MPEC (2 trials[17,18]), or RFA with sham procedure (1 trial[19]). Sample sizes were small and follow-up times were short. Because the types of patients and the treatment protocols (e.g., number of treatment sessions) varied a meta-analysis was not used.

In the non-comparative studies of endoscopic techniques (single arm clinical trials or case series), the treatment protocols (e.g., number of treatment sessions), outcomes measured, and follow-up times also differed. There were few studies that reported long-term follow up results, and so pooling of study outcome and adverse event results is limited. As well, the number of treatment sessions provided before outcomes were measured was not often reported, although in many cases, it was after a single ablation. Patients often received interventions in addition to the study treatment. For example, in most studies EMR was performed during endoscopy to confirm the diagnosis of dysplasia. In addition, if one treatment failed to eradicate the dysplasia another treatment would typically be administered. The analyses of outcomes did not usually account for the effects of these additional interventions.

### Safety

Adverse events reported for individual studies of esophagectomy and endoscopic alternatives are summarized in Table 4 and Additional file 9. The pooled estimates for esophagectomy and endoscopic treatments are shown in Tables 5 and 6. There were 2 deaths [20,21] attributed to esophagectomy among the 170 patients who underwent the procedure (a mortality rate of 1.2%). (The surgical studies employed various approaches; in some studies, the actual approach was not specified, and in the others, a number of approaches were used (i.e., the patients did not all have the same surgical approach). Therefore, data could not be analysed according to individual surgical approaches). In contrast, 1 death [13] was reported in the 2831 patients who received endoscopic treatment (0.04%). This death was due to cardiac arrhythmia in a patient who received PDT with aminolevulinic acid (ALA) at a dose of 60 mg/kg of body weight[13].

**Table 4:** Studies of adverse events post-esophagectomy

		Adverse event rates (No. of patients who suffered adverse events/No. of patients who received esophagectomy)									
**Study**	**No. of patients**	A.leak*	CV compl.*	Del. gastric emptying*	**Mortality**	Pneumon.*	Pulm. compl.*	Pulm. embol.*	Small bowel perf.*	**Strictures**	Wound infec.*

*Comparative studies*											
Prasad GA, et al. (2007)[21]	70	0% (0/70)	0% (0/70)	0% (0/70)	1.4% (1/70)	0% (0/70)	0% (0/70)	0% (0/70)	0% (0/70)	12.9% (9/70)	0% (0/70)
Reed MF, et al. (2005)[20]	49	4.1% (2/49)	0% (0/49)	0% (0/49)	2.0% (1/49)	0% (0/49)	0% (0/49)	0% (0/49)	0% (0/49)	0% (0/49)	0% (0/49)
Thomas T, et al. (2005)[55]	8	Not reported	Not reported	Not reported	Not reported	Not reported	Not reported	Not reported	Not reported	Not reported	Not reported
*Non-comparative studies*											
Ferguson MK, et al. (1997)[107]	15	73.3% (11/15)	20.0% (3/15)	0% (0/15)	0% (0/15)	0% (0/15)	26.7% (4/15)	0% (0/15)	0% (0/15)	0% (0/15)	33.3% (5/15)
Nguyen NT, et al. (2000)[108]	12	0% (0/12)	0% (0/12)	25.0% (3/12)	0% (0/12)	0% (0/12)	16.7% (2/12)	0% (0/12)	8.3% (1/12)	0% (0/12)	8.3% (1/12)
Romagnoli R (2003)[109]	33	Not reported	Not reported	Not reported	Not reported	Not reported	Not reported	Not reported	Not reported	Not reported	Not reported
Sujendran V, et al. (2005)[110]	17	17.6% (3/17)	0% (0/17)	0% (0/17)	0% (0/17)	17.6% (3/17)	5.9% (1/17)	0% (0/17)	0% (0/17)	0% (0/17)	0% (0/17)
Thomson BNJ & Cade RJ (2003)[111]	7	0% (0/7)	0% (0/7)	0% (0/7)	0% (0/7)	0% (0/7)	0% (0/7)	14.3% (1/7)	0% (0/7)	0% (0/7)	14.3% (1/7)
Pooled total	211	9.4% (16/170) 0-73.3%‡	1.8% (3/170) 0-20.0%‡	1.8% (3/170) 0-25.0%‡	1.2% (2/170) 0-2.0%‡	1.8% (3/170) 0-17.6%‡	4.1% (7/170) 0-26.7%‡	0.6% (1/170) 0-14.3%‡	0.6% (1/170) 0-8.3%‡	5.3% (9/170) 0-12.9%‡	4.1% (7/170) 0-33.3%‡

***Notes: ***(1) *A.leak (anastomotic leak), CV compl. (cardiovascular complication), Del. gastric emptying (delayed gastric emptying), Pneumon. (pneumonia), Pulm. compl. (pulmonary complication), Pulm. embol. (pulmonary embolsim), Small bowel perf. (small bowel perforation), Wound infec. (wound infection) (2) ‡Ranges of the adverse event rates post-esophagectomy for the included studies

**Table 5:** Summary of adverse events post-esophagectomy

A. leak*	CV compl.*	Del. gastric emptying*	Mortality	Pneumon.*	Pulm. compl.*	Pulm. embol.*	Small bowel perf.*	Strictures	Wound infec.*
9.4%٭	1.8%٭	1.8%٭	1.2%٭	1.8%٭	4.1%٭	0.6%٭	0.6%٭	5.3%٭	4.1%٭
16/170‡	3/170‡	3/170‡	2/170‡	3/170‡	7/170‡	1/170‡	1/170‡	9/170‡	7/170‡
0 -73.3%†	0 -20.0%†	0 - 25.0%†	0 - 2.0%†	0 - 17.6%†	0 - 26.7%†	0 - 14.3%†	0 - 8.3%†	0 - 12.9%†	0 - 33.3%†

***Notes: ***(1) *A.leak (anastomotic leak), CV compl. (cardiovascular complication), Del. gastric emptying (delayed gastric emptying), Pneumon. (pneumonia), Pulm. compl. (pulmonary complication), Pulm. embol. (pulmonary embolism), Small bowel perf. (small bowel perforation), Wound infec. (wound infection) (2) ٭Adverse event rates post-esophagectomy, ‡ No. of patients who suffered adverse events/No. of patients who received esophagectomy, † Ranges of the adverse event rates post-esophagectomy for the included studies

In the studies that reported bleeding complications following endoscopic treatments, 4 of 2218 patients (0.2%) experienced bleeding requiring transfusions: 1 after PDT[22], 2 after APC[23] and 1 after laser ablation[24]. Strictures were most frequently reported with porfimer sodium PDT (18.5%), followed by laser ablation (4.4%) and APC (2.9%) (Table 6). Although there were no perforations reported in the PDT studies that used a single photosensitizer (reported in Table 6), there was a perforation reported in the Prasad et al study [21], which compared esophagectomy with PDT using two different photosensitizers. Since it was impossible to separate the patients according to the photosensitizer type, this study was not included in Table 6. Patients experiencing dysphagia and odynophagia varied across the treatment modalities, but were highest with MPEC (Table 6). Photosensitivity following PDT was more common with porfimer sodium (26.4%) than with ALA (ranging from 0% to 13.6%). However, in a small series of 5 patients who received hematoporphyrin derivative (HpD), 40% experienced photosensitivity reactions (Table 6).

**Table 6:** Summary of adverse events post-endoscopic treatments

	Adverse event rates post- endoscopic treatments (No. of patients who suffered adverse events/No. of patients who received treatments)					
**Study**	**Dysphagia**	Photosen.*	**Stricture**	Perfor.*	Odynoph.*	Bleed.*

***PDT***						
**ALA 15 mg/kg**	15.4% (2/13)	13.6% (3/22) 0-23.1%†	0% (0/22)	0% (0/22)	0% (0/22)	0% (0/22)
**ALA 30 mg/kg**	0% (0/106)	5.7% (6/106) 0-14.7%†	0% (0/106)	0% (0/106)	0.9% (1/106) 0-2.9%†	0%^(4) ^(0/90)
**ALA 40 mg/kg**	0% (0/22)	0% (0/22)	0% (0/22)	0% (0/22)	0% (0/22)	0% (0/22)
**ALA**^**(1) **^**60 mg/kg**	2.7% (4/148) 0-40.0%†	4.3% (6/140) 0-75.0%†	1.4% (2/148) 0-12.5%†	0% (0/148)	16.2% (24/148) 0-92.3%†	0.9%^(4) ^(1/115) 0-7.7%†
**HpD 1.5 mg/kg**	0% (0/59)	40.0% (2/5)	0% (0/59)	0% (0/59)	0% (0/5)	0% (0/59)
**mTHPC 0.15 mg/kg**	-**	-**	-**	-**	-**	-**
**Porfimer sodium(2 mg/kg)**	6.6% (26/394) 0-18.8%†	26.4% (104/394) 0-68.8%†	18.5% 73/394 0-37.5%†	0% (0/394)	0% (0/394)	0% (0/394)
***Other endoscopic treatments***						
**APC**	3.8% (27/719) 0-100%†	0% (0/719)	2.9% (21/719) 0-23.1%†	0.3% (2/719) 0-3.4%†	11.8% (85/719) 0-94.1%†	0.4%^(2) ^(3/719) 0-3.9%†
**Cryoablation**	9.1% (1/11)	0% (0/11)	0% (0/11)	0% (0/11)	0% (0/11)	0% (0/11)
**Combined EMR & PDT**	0% (0/6)	0% (0/6)	0% (0/6)	0% (0/6)	0% (0/6)	0% (0/6)
**Thermocoagualtion**	0% (0/13)	0% (0/13)	0% (0/13)	0% (0/13)	0% (0/13)	0% (0/13)
**EMR**	0% (0/32)	0% (0/32)	0% (0/32)	0% (0/32)	0% (0/32)	9.4% (3/32) 0-25.0%†
**Laser ablation**	0% (0/68)	0% (0/68)	4.4% (3/68) 0-12.5%†	1.5% (1/68) 0-4.8%†	0% (0/68)	1.5%^(5) ^(1/68) 0-4.8%†
**MPEC**	19.4% (18/93) 0-40.7%†	0% (0/93)	1.1% (1/93) 0-3.7%†	0% (0/93)	16.1% (15/93) 0-40.7%†	1.1% (1/93) 0-10.0%†
**RFA**	1.4% (8/574) 0-23.1%†	0% (0/574)	1.9% (11/574) 0-6.1%†	0% (0/574)	0.5% (3/574) 0-23.1%†	0.5% (3/574) 0-1.6%†

***Notes: ***(1) 1 patient suffered cardiac arrhythmia resulting in sudden death [13]. (2) 2 patients required blood transfusions. (3) ALA (aminolevulinic acid), APC (argon plasma coagulation), EMR (endoscopic mucosal resection), HpD (hematoporphyrin derivative), MPEC (multipolar electrocoagulation), mTHPC (meta-tetrahydroxyphenylchlorin), PDT (photodynamic therapy), RFA (radiofrequency ablation) (4) 1 patient required blood transfusion, but it cannot be definitely attributed to the 30 mg/kg treatment or the 60 mg/kg ALA treatment. (5) 1 patient required blood transfusion. (6) * Photosen. (photosensitivity), Perfor. (perforation), Odynoph. (odynophagia), Bleed. (bleeding), ** - (pooled total not available), † Ranges of the adverse event rates post-endoscopic treatments for the included studies

The most commonly reported adverse events associated with esophagectomy were anastomotic leaks (9.4%), strictures (5.3%), wound infections (4.1%) and pulmonary complications (4.1%) (Table 5). None of the studies discussed the relationship between adverse events and clinician experience.

### Efficacy or effectiveness

Values reported for the complete eradication of BE or high grade dysplasia (HGD) with endoscopic treatments are presented in Additional files 10 and 11. Pooled values for complete eradication of BE and HGD with endoscopic treatments are presented in Table 7. For the purposes of this analysis, only the complete eradication rates reported in the individual studies within the first 3 months after ablation were included. Few studies provided enough data on longer follow up periods to make pooling of the data meaningful. The studies did not all report the number of ablations that were provided before the outcome was measured, and in many cases, the authors reported a range of number of treatments.

**Table 7:** Summary of complete eradication (CE) of BE and HGD post- endoscopic treatments

Study	CE rates of BE post -treatments (No. of patients who achieved CE of BE/No. of patients who received treatments)		CE rates of HGD post -treatments (No. of patients who achieved CE of HGD/No. of patients who received treatments)	
	
	Up to 3 months	At 12 months	Up to 3 months	At 12 months
***PDT***				
**ALA 15 mg/kg**	30.4% (7/23) 21.4-44.4%†	-**	No studies of HGD	No studies of HGD
**ALA 30 mg/kg**	43.9% (18/41) 14.3-50.0%†	-**	100% (4/4)	-**
**ALA 40 mg/kg**	0% (0/2)	-**	68.2% (15/22) 0-75.0%†	-**
**ALA 60 mg/kg**	19.2% (5/26)	-**	96.6% (28/29) 96.3-100%†	-**
**HpD 1.5 mg/kg**	0% (0/5)	-**	100% (1/1)	100% (1/1)
**mTHPC 0.15 mg/kg**	16.7% (1/6)	-**	66.7% (4/6)	-**
**Porfimer sodium (2 mg/kg)**	51.6% (94/182) 26.3-56.4%†	15.4% (2/13)	77.5% (62/80)	100% (2/2)
***Other endoscopic treatments***				
**APC**	85.5% (372/435) 0-100%†	64.9% (50/77) 0-93.8%†	85.7% (6/7)	0% (0/0)
**Cryoablation**	81.8% (9/11)	63.6% (7/11)	100% (1/1)	-**
**Combined EMR & PDT**	-**	-**	66.7 (2/3)	-**
**Thermocoagulation**	100% (13/13)	-**	No studies of HGD	No studies of HGD
**EMR**	100% (1/1)	-**	96.3% (26/27) 92.9-100%†	83.3% (10/12)
**Laser ablation**	77.3% (58/75) 22.2-100%†	-**	-**	-**
**MPEC**	88.5% (23/26)	100% (10/10)	No studies of HGD	No studies of HGD
**RFA**	69.0% (118/171) 21.9-97.7%†	72.3% (170/235) 46.2%-92.6%†	90.3% (93/103) 90.2-90.9%†	81.0% (34/42)

***Notes: ***(1) ALA (aminolevulinic acid), APC (argon plasma coagulation), EMR (endoscopic mucosal resection), HpD (hematoporphyrin derivative), MPEC (multipolar electrocoagulation), mTHPC (meta-tetrahydroxyphenylchlorin), PDT (photodynamic therapy), RFA (radiofrequency ablation) (2) **- (pooled total not available), † Ranges of the complete eradication rates post-endoscopic treatments for the included studies

Results of 2 of the 3 RCTs of PDT (with ALA) versus APC in patients with BE demonstrated a significant difference in complete eradication, favouring APC over PDT[13,14]. Specifically, the complete eradication rates in the APC group were almost double that of the PDT group (Additional file 10). In the third RCT, complete eradication rates were not reported for both groups of patients[16].

Two RCTs reported on complete eradication of HGD[15,19]. In the RCT which compared PDT using porfimer sodium to APC, there was no statistically significant difference in complete eradication rate between treatment groups at 4 months follow-up[15]. In the RCT which compared RFA with sham procedure, there was a statistically significant difference in complete eradication rate between the group treated by RFA (81.0%) and the control group (19.0%) at 12 months follow-up[19].

## Discussion

Endoscopic therapies appear to be viable and effective treatment options for Barrett's esophagus with high grade dysplasia. All of the endoscopic therapies are safer (i.e., have fewer adverse events and lower mortality rates) than esophagectomy.

Some key questions regarding these treatments cannot yet be answered and further studies are needed to address these "gaps in the evidence". In particular, we need studies:

• to confirm the long-term safety of these endoscopic treatments, and their effectiveness in preventing esophageal cancer

• to identify the endoscopic treatments (or combinations of treatments) that produce the best outcomes

• to determine whether or not continued drug therapy (e.g., with proton pump inhibitors) or surgery (e.g., fundoplication) to treat gastroesophageal reflux is beneficial after endoscopic treatment of dysplasia

• to provide guidance on the optimal frequency of post-treatment endoscopic surveillance for patients with Barrett's esophagus

• to measure patient preferences for, and quality of life after, the different endoscopic treatments.

## Conclusions

Given the current limitations in the evidence (in terms of both quantity and quality of studies), it was not possible to conclusively determine the comparative effectiveness of the different endoscopic treatments. However, the evidence suggests that endoscopic treatments are safe and reasonably effective alternatives to esophagectomy for patients with Barrett's esophagus with high grade dysplasia. Endoscopic treatments have the additional advantages of being outpatient procedures with shorter recovery times. They also provide treatment options for patients who would not be considered for esophagectomy due to other health conditions.

Of the endoscopic therapies, photosensitivity is only an issue with photodynamic therapy (more so with porfimer sodium than with other photosensitizing agents). Preventing adverse events due to photosensitivity requires patient and caregiver compliance and education.

There appears to be little difference between the endoscopic technologies in terms of overall efficacy. Patient and physician preferences, and the local availability of the different technologies will likely guide decision making. A combination of different endoscopic treatments may provide the best outcomes. Given that relatively few patients need these treatments each year, offering them at specialized centres will concentrate clinical expertise and be the most cost-effective approach.

## Competing interests

CW was the recipient of a one time clinical implementation grant from Axcan in 2007, and has given educational talks funded through unrestricted grants from Axcan. The remaining author(s) declare that they have no competing interests

## Authors' contributions

DM and CW made substantial contributions to the conception and design of the study, to interpretation of the data, and have reviewed and revised the manuscript for important intellectual and clinical content. TS made a substantial contribution to the conception and design of the study, interpretation of data, and was involved in writing and critically reviewing drafts of the manuscript. HW was involved in the acquisition, analysis and interpretation of the data and making major revisions to draft manuscripts. DL was involved in the conception and design of the study, acquisition and analysis of data and drafting the manuscript. All authors have given approval for submission of this version.

## Pre-publication history

The pre-publication history for this paper can be accessed here:

http://www.biomedcentral.com/1471-230X/10/111/prepub

## Supplementary Material

Additional file 1 **Studies of photodynamic therapy (PDT) for Barrett's esophagus with/without dysplasia**. Details of study and patient characteristics, outcomes and study quality of the included studies of PDT for BE with/without dysplasia are presented in Additional file 1. Click here for file

Additional file 2 **Studies of argon plasma coagulation (APC) for Barrett's esophagus with/without dysplasia**. Details of study and patient characteristics, outcomes and study quality of the included studies of APC for BE with/without dysplasia are presented in Additional file 2. Click here for file

Additional file 3 **Studies of cryoablation, combined endoscopic mucosal resection (EMR) and photodynamic therapy (PDT), and thermocoagulation for Barrett's esophagus with/without dysplasia**. Details of study and patient characteristics, outcomes and study quality of the included studies of cryoablation, combined EMR and PDT, and thermocoagulation for BE with/without dysplasia are presented in Additional file 3. Click here for file

Additional file 4 **Studies of endoscopic mucosal resection (EMR) for Barrett's esophagus with/without dysplasia**. Details of study and patient characteristics, outcomes and study quality of the included studies of EMR for BE with/without dysplasia are presented in Additional file 4. Click here for file

Additional file 5 **Studies of laser ablation for Barrett's esophagus with/without dysplasia**. Details of study and patient characteristics, outcomes and study quality of the included studies of laser ablation for BE with/without dysplasia are presented in Additional file 5. Click here for file

Additional file 6 **Studies of multipolar electrocoaguation (MPEC) for Barrett's esophagus with/without dysplasia**. Details of study and patient characteristics, outcomes and study quality of the included studies of MPEC for BE with/without dysplasia are presented in Additional file 6. Click here for file

Additional file 7 **Studies of radiofrequency ablation (RFA) for Barrett's esophagus with/without dysplasia**. Details of study and patient characteristics, outcomes and study quality of the included studies of RFA for BE with/without dysplasia are presented in Additional file 7. Click here for file

Additional file 8 **Studies of esophagectomy for Barrett's esophagus with/without dysplasia**. Details of study and patient characteristics, outcomes and study quality of the included studies of esophagectomy for BE with/without dysplasia are presented in Additional file 8. Click here for file

Additional file 9 **Studies of adverse events (endoscopic treatments)**. Adverse events reported for individual studies of endoscopic alternatives are presented in Additional file 9. Click here for file

Additional file 10 **Studies of complete eradication of Barrett's esophagus (endoscopic treatments)**. Values reported for the complete eradication of BE with endoscopic treatments are presented in Additional file 10. Click here for file

Additional file 11 **Studies of complete eradication of high grade dysplasia (endoscopic treatments)**. Values reported for the complete eradication of HGD with endoscopic treatments are presented in Additional file 11. Click here for file
