# Supplementary material for: Endoscopic treatments for Barrett's esophagus: a systematic review of safety and effectiveness compared to esophagectomy
Source: BMC Gastroenterol. 2010 Sep 27;10:111. doi: 10.1186/1471-230X-10-111 (PMC2955687; doi:10.1186/1471-230X-10-111)
Supplement: Additional file 1 — Studies of photodynamic therapy (PDT) for Barrett's esophagus with/without dysplasia. Details of study and patient characteristics, outcomes and study quality of the included studies of PDT for BE with/without dysplasia are presented in Additional file 1. [file 1471-230X-10-111-S1.DOC]

| **Study authors (year published)**  Additional file 1. Studies of photodynamic therapy (PDT) for Barrett's esophagus with/without dysplasia | **Cancer / Cell Type** | **Study Design** | **Patients** | **Intervention** | **Outcome Measures** | **Findings** | **Study quality** |
| --- | --- | --- | --- | --- | --- | --- | --- |
| **ALA 15mg/kg administered orally** | | | | | | | |
| *Comparative studies* | | | | | | | |
| None | | | | | | | |
| *Non-comparative studies* | | | | | | | |
| Ortner MA, et al. (2002)[26] | BE (7 patients)  BE + LGD (7 patients) | Clinical trial  Single centre  Prospective  *Countries:* Germany  *Length of follow-up:*  Mean: 32.6 months  Range: 12 to 48 months | *Number of patients:* 14  *Gender:*  Male: 12  Female:2  *Age:*  Mean: 61.8 yrs  *Prior treatments:* none reported  *Length of Barrett’s:* not reported  *Inclusion criteria:* none notable  *Exclusion criteria:*  Allergy to OM  Porphyria  Previous esophageal cancer  HGD  Contraindications for endoscopy  Clotting disturbances  Pregnant or lactating | PDT  *Drug:* 5-ALA  *Dose:* 15mg/kg  *Route of administration:* topical  *Light source*: argon dye laser @632nm  *Light dose:* 90 to 120 J/cm^2^  *Time to photoactivation:* 1.5 to 2 hours  *Treatment time:* not recorded  *Number of sessions:*  Mean: 1.4 sessions / patient  *Co-interventions:*  OM 80 mg/day for 2 months | *Outcomes:*  CR of BE    CR of LGD  Partial response of BE  *Adverse events* | *Outcomes:*  CR of BE:  - at 3 months: 3/14 patients (21%)  - at 6 months: 4/14 patients (29%)    CR of LGD:  - at 3 months: 4/7 patients (57%)  - at 6 months: 5/7 patients (71%)  Partial response of BE:  - at 3 months: 11/14 patients (79%)  - at 6 months: 10/14 patients (71%)  *Adverse events:*  Chest pain and dysphagia: 2/13 patients (15%)  Photosensitivity: 3/13 patients (23%)  Strictures: 0/13 patients (0%)  Hepatotoxicity: 0/13 patients (0%) | 4 |
| Ortner M, et al. (1997)[27] | BE | Case series  Single centre  *Countries:* Germany  *Length of follow-up:*  2 months | *Number of patients:* 9  *Gender:* not reported  *Age:* not reported  *Prior treatments:* none reported  *Length of Barrett’s:* not reported  *Inclusion criteria:* none notable  *Exclusion criteria:* none notable | PDT  *Drug:* 5-ALA  *Dose:* 14 to 16 mg/kg  *Route of administration:* topical spray  *Light source*: argon dye laser @ 632nm  *Light dose:* 180J/cm^2^  *Time to photoactivation:* 1.5 to 2 hours  *Treatment time:* 180 to 300 seconds  *Number of sessions:* 1 session / patient  *Co-interventions:*  OM 40 mg 4 times daily for 2 months | *Outcomes:*  CR of BE  Partial response of BE  Non-response of BE  *Adverse events* | *Outcomes:*  CR of BE at 2 months: 4/9 patients (44%)  Partial response of BE at 2 months: 3/9 patients (33%)  Non-response of BE at 2 months: 2/9 patients (22%)  *Adverse events:*  Chest pain: occasionally  Dysphagia, mild: occasionally | 4 |
| **ALA 30mg/kg administered orally** | | | | | | | |
| *Comparative studies* | | | | | | | |
| Kelty CJ, et al. (2004)[14] | BE | RCT  Single centre  Prospective  PDT vs. APC  *Countries:* UK  *Length of follow-up*: 24 months | *Number of patients:* 72  (PDT Group:35 patients; APC Group: 37 patients)  PDT Group  *Gender:*  Male: 28  Female: 7  *Age:*  Median: 61 yrs  Range: 33 to 83 yrs  APC Group  *Gender:*  Male: 30  Female: 7  *Age:*  Median: 59 yrs  Range: 28 to 79 yrs  *Prior treatments:* none reported  *Length of Barrett’s:*  PDT Group  Median: 4 cm  Range: 2 to 15 cm  APC Group  Median: 4 cm  Range: 2 to 8 cm  *Inclustion criteria:* none notable  *Exclusion criteria:* none notable | PDT vs. APC  PDT Group  *Drug:* 5-ALA  *Dose:* 30 mg/kg  *Route of administration:* oral  *Light source*: diode laser @ 633 nm  *Light dose:* 85 J/cm^2^  *Time to photoactivation:* 4 to 6 hours post ALA  *Treatment time:* not reported  *Number of sessions:*  Median: 5 sessions  Range: 1 to 5 sessions  Max allowed: 5 sessions  APC Group  *Gas flow:*  2L/minute  *Power:* 65 watts  *Number of sessions:*  Median: 3 sessions  Range: 1 to 5 sessions  Max allowed: 5 sessions  *Co-interventions:* none reported | *Outcomes:*  CR of BE (assessed through endoscopy and 4 quadrant biopsy every 2 cm)  Partial response of BE  Number of treatments to achieve CR of BE  *Adverse events* | *Outcomes:*  CR of BE at 4 weeks:  -PDT Group: 17/34 patients (50%)  -APC Group: 33/34 patients (97%)  Partial response of BE:  -PDT Group: 17/34 patients (50%)  -APC Group: 1/34 patients (3%)  Number of treatments to achieve CR of BE:  PDT Group  Median: 2 treatments  Range: 1 to 4 treatments  APC Group  Median: 3 treatments  Range: 1 to 5 treatments  *Adverse events:*  PDT Group  Nausea / vomiting: 11/34 patients (32%)  Photosensitivity: 5/34 patients (15 %)  Hypotension: 2/34 patients (6%)  Chest pain: 1/34 patients (3%)  Odynophagia: 1/34 patients (3%)  Dysphagia secondary to strictures: 0/34 patients (0%)  Elevated liver enzymes, mild: 4/34 patients (12%)  Buried glands (4 week follow-up): 4/17 patients (24%)  APC Group  Nausea / vomiting; photosensitivity; hypotension; chest pain; elevated liver enzymes, mild: 0/34 patients (0%)  Odynophagia: 32/34 patients (94%)  Dysphagia secondary to strictures: 1/34 patients (3%)  Buried glands (4 week follow-up): 7/33 patients (21%) | 1 |
| *Non-comparative studies* | | | | | | | |
| Akroyd R, et al. (2003)[28] | BE + LGD | Case series  Single centre  Prospective  *Countries:* UK  *Length of follow-up:*  Median: 53 months  Range: 18 to 68 months | *Number of patients:* 40  *Gender:*  Male: 36  Female: 4  *Age:*  Median: 61 yrs  Range: 34 to 86 yrs  *Prior treatments:* not reported  *Length of Barrett’s:*  Median: 6 cm  Range: 3 to 18 cm  *Inclusion criteria:* none notable  *Exclusion criteria:* none notable | PDT  *Drug:* ALA  *Dose:* 30 mg/kg  *Route of administration:* oral  *Light source*: copper vapour laser @514nm  *Light dose:* 60 J/cm^2^  *Time to photoactivation:* 4 hours  *Treatment time:* not reported  *Number of sessions:* 1 session / patient  *Co-interventions:*  OM 20-40 mg/day  Endoscopy at 1, 6, 12 months | *Outcomes:*  CR of LGD  Reduction of BE area  *Adverse events* | *Outcomes:*  CR of LGD  -at 1 month: 40/40 patients (100%)  -at 24 months: 38/38 patients (100%)  -at 60 months: 15/15 patients (100%)  Reduction of BE area:  Median: 30%  Range: 0 to 90%  *Adverse events:*  Buried glands: 1/40 patients (2.5%)  Strictures: 0/40 patients (0%)  Discomfort, duration ≤ 3 days: most patients  Nausea and vomiting ≤ 24 hours: most patients  Photosensitivity, mild (patient exposed to direct sun for several hours): 1/40 patients (2.5%) | 4 |
| Ackroyd R, et al. (1999)[30]  * Information extracted for BE or HGD patients only | BE + LGD (3 patients)  BE + HGD (4 patients) | Case series  Single centre  Prospective  *Countries:* UK  *Length of follow-up:* 28 months | *Number of patients:* 7  *Gender:*  Male: 5  Female: 2  *Age:*  Mean: 68.3 yrs  Range: 49 to 83 yrs  *Prior treatments:* not reported  *Length of Barrett’s:* not reported  *Inclusion criteria:* none notable  *Exclusion criteria:* none notable | PDT  *Drug:* ALA  *Dose:* 30 mg/kg  *Route of administration:* oral  *Light source*: copper vapour laser @514nm or 630nm  *Light dose:*  Mean: 80 J/cm^2^  Range: 50 to 100 J/cm^2^  *Time to photoactivation:* 4 hours  *Treatment time:* not reported  *Number of sessions:* 1 session / patient  *Co-interventions:*  OM 20 mg/day | *Outcomes:*  CR of BE  CR of HGD  CR of LGD  Reduction of BE area  Survival  *Adverse events:* No BE or HGD specific information available. | *Outcomes:*  CR of BE  -at 1 month: 1/7 patients (14%)  -at 24 months: 1/7 patients (14%)  CR of HGD  -at 1 month: 4/4 patients (100%)  -at 24 months: 4/4 patients (100%)  CR of LGD  -at 1 month: 3/3 patients (100%)  -at 24 months: 3/3 patients (100%)  Reduction of BE area at 1 month:  Mean: 44 %  Range: 10 to 100%  Survival  -at 1 month: 7/7 patients (100%)  -at 24 months: 7/7 patients (100%) | 4 |
| Ackroyd R, et al. (1997)[29] *  * Information extracted for BE or HGD patients only | BE + LGD (1 patient)  BE + HGD (4 patients) | Case series  Single centre  *Countries:* UK  *Length of follow-up:* not reported | *Number of patients:* 5  *Gender:* not reported  *Age:* not reported  *Prior treatments:* not reported  *Length of Barrett’s:* not reported  *Inclusion criteria:* none notable  *Exclusion criteria:* none notable | PDT  *Drug:* ALA  *Dose:* 30 mg/kg  *Route of administration:* oral  *Light source*: copper vapour laser @514nm  *Light dose:* 1000J/cm^2^  *Time to photoactivation:* 4 hours  *Treatment time:* 1000 seconds of laser activation  *Number of sessions:* 1 session / patient  *Co-interventions:* none reported | *Outcomes:*  CR of HGD  CR of LGD  Reduction in BE area  *Adverse events:* none | *Outcomes:*  CR of HGD at unknown follow-up: 4/4 patients (100%)  CR of LGD at unknown follow-up: 1/1 patients (100%)  Reduction in BE area:  Mean: 48 %  Range: 10 to 70 % | 4 |
| Mackenzie G, et al. (2005)[31]^8^ | BE + HGD | RCT  Single centre  Prospective  PDT (red light) vs. PDT (green light)  *Countries:* not reported  *Length of follow-up:* not reported | *Number of patients:*16  *Gender:* not reported  *Age:* not reported  PDT Red Light  *Number of patients:* not reported  PDT Green Light  *Number of patients:* not reported  *Prior treatments:*  EMR of nodular dysplasia in 4 patients  *Length of Barrett’s:* not reported  *Inclusion criteria:* none notable  *Exclusion criteria:* none notable | PDT with red light  *Drug: 5-*ALA  *Dose:* 30 mg/kg  *Route of administration:* oral  *Light source*: 600nm laser  *Light dose:* not reported  *Time to photoactivation:* 4 hours  *Treatment time:* not reported  *Number of sessions:*  Mean: 2.15 sessions / patient  PDT with green light  *Light source*: 520 to 570nm laser  *Number of sessions*:  Mean 2.15 sessions / patient  Other details as above  *Co-interventions:*  Treatments preceded by EMR | *Outcomes:*  Number of sessions reporting CR of dysplasia  *Adverse events:* no major complications. | *Outcomes:*  Number of sessions reporting CR of dysplasia:  -Red light: 4/17 sessions (24%)  -Green light: 1/19 sessions (5%) | 4 |
| Mackenzie G, et al. (2005)[22] | BE + HGD | Case series  Single centre  *Countries:* UK  *Length of follow-up:*  Mean: 15.8 months  Range: 1 to 72 months | *Number of patients:* 51*  *Gender:* not reported  *Age*: not reported  Group A  *Number of patients:* 21 patients  *Gender:* not reported  *Age:* not reported  Group B  *Number of patients:* 12 patients  *Gender:* not reported  *Age:* not reported  Group C  *Number of patients:* 16 patients  *Gender:* not reported  *Age:* not reported  *Prior treatments: none reported*  *Length of Barrett’s:* not reported  *Inclusion criteria:* none notable  *Exclusion criteria:* none notable  * 3 patients unaccounted for. | PDT  *Drug:* ALA  *Route of administration:* oral  *Light source*: not reported  *Time to photoactivation:* not reported  *Treatment time:* not reported  *Number of sessions:*  Mean: 1.74 sessions / patient  Group A  *Dose:* 60 mg/kg  *Light dose:* 1000J/ cm^2^  Other details as above  Group B  *Dose:* 60 mg/kg  *Light dose:* 500 to 750J/cm^2^  Other details as above  Group C  *Dose:* 30 mg/kg  *Light dose:* 1000J/ cm^2^  Other details as above  *Co-interventions:* none reported | *Outcomes:*  CR of HGD:  *Adverse events* | *Outcomes:*  CR of dysplasia at 15.8 months (mean):  Group A: 16/21 patients (76%)  Group B: 2/12 patients (17%)  Group C: 5/16 patient (31%)  All patients: 23/49 patients (47%)  *Adverse events:*  Strictures and photosensitivity: 0/51 patients (0%)  Hypotension: 3/51 patients (6%)  GI bleed requiring transfusion: 1/51 patients (2%) | 4 |
| **ALA 40mg/kg administered orally** | | | | | | | |
| *Comparative studies* | | | | | | | |
| None | | | | | | | |
| *Non-comparative studies* | | | | | | | |
| Peters F, et al. (2005)[32] | BE + HGD | Case series  Single centre  *Countries:* Netherlands  *Length of follow-up:*  Mean: 30 months  Range: 22 to 31 months | *Number of patients*: 20 patients  *Gender:*  Male: 16  Female: 4  *Age:*  Mean: 69 yrs  Range: 59 to 74 yrs  *Prior treatments:*  Diagnostic EMR for focal lesions  *Length of Barrett’s: n*ot reported  *Inclusion criteria:*  Ineligible for or refused surgery  *Exclusion criteria:* none notable | PDT  *Drug:* ALA  *Dose:* 40 mg/kg  *Route of administration:* oral  *Light source*: KTP/Nd:YAG laser @ 600 nm  *Light dose:* 100J/cm^2^  *Time to photoactivation:* not reported  *Treatment time:* not reported  *Number of sessions:*  Mean: 1 session / patient  Range: 1 to 2 sessions / patient  *Co-interventions:*  Ranitidine 300 mg @ night for 1st week  OM 40 mg twice daily for first week  Esomeprazole 40 mg twice daily from 2nd week on | *Outcomes:*  CR of HGD (assessed through endoscopy with 4 quadrant biopsies every 2 cm)  *Adverse events* | *Outcomes:*  CR of dysplasia at:  - at 3 months: 15 /20 patients (78%)  - at 23 months: 11/20 patients (55%)  *Adverse events:*  Hematemesis: 1/20 patients (5%)  Hypotension: 2/20 patients (10%)  Atrial fibrillation: 1/20 patients (5%)  Buried glands (at mean =30 months): 8/15 patients (53%) | 4 |
| Van Hillegersberg R, et al. (2003)[33] | BE + HGD | Case report  Single centre  Retrospective  *Countries:* Netherlands  *Length of follow-up:*  Mean: 6 months  Range: 5 to 8 months | *Number of patients:* 2  *Gender:*  Male: 1  Female: 1  *Age:*  Mean: 65 yrs  Range: 61 to 69 yrs  *Prior treatments:*  PPI, unspecified (1/2 patients)  *Length of Barrett’s*: not reported  *Inclusion criteria:* none notable  *Exclusion criteria:* none notable | PDT  *Drug:* ALA  *Dose:* 40 mg/kg  *Route of administration:* oral  *Light source*: laser @630nm  *Light dose:* 70 to 100J/cm^2^  *Time to photoactivation:* 3.3 to 5.9 hours  *Treatment time:* not reported  *Number of sessions:*  Mean: 2 sessions / patient  Range: 1 to 3 sessions  *Co-interventions:*  High dose PPI  Ranitidine 150 mg as needed. | *Outcomes:*  CR of BE (assessed through endoscopy with random biopsies)  CR of HGD (assessed through endoscopy with random biopsies)  Progression to cancer  *Adverse events* | *Outcomes:*  CR of BE at 3 months: 0/2 patients (0%)  CR of HGD at 3 months: 0/2 patients (0%)  Progression to cancer at 6 months: 2/2 patients (100%)  *Adverse events:*  Nausea and vomiting: 1/2 patients (50%) | 4 |
| **ALA 60mg/kg administered orally** | | | | | | | |
| *Comparative studies* | | | | | | | |
| Behrens A, et al. (2005)[25] | BE + HGD | Cohort study  Single centre  Prospective  PDT vs. EMR vs. PDT + EMR  *Countries:* Germany  *Length of follow-up:*  Mean: 38 months  Range: 7 to 61 months | *Number of patients:* 44  (PDT Group: 27 patients; EMR Group: 14 patients; PDT+EMR: 3 patients)  *Gender:*  Male: 38  Female: 6  *Age:*  Mean:61 yrs  Range: 33 to 79 yrs  PDT Group  *Number of patients:* 27 patients  *Gender:* not reported  *Age:* not reported  EMR Group  *Number of patients:* 14 patients  *Gender:* not reported  *Age:* not reported  PDT + EMR Group  *Number of patients:* 3 patients  *Gender:* not reported  *Age:* not reported  *Prior treatments:* none reported  *Length of Barrett’s:* not reported  *Inclusion criteria:* none notable  *Exclusion criteria:* none notable | PDT vs. EMR vs. PDT + EMR  PDT Group  Patients with microscopic / histologic HGD  *Drug:* 5-ALA  *Dose:* 60 mg/kg  *Route of administration:* oral  *Light source*: dye laser @ 630 to 635nm  *Light dose:* not reported  *Time to photoactivation:* 4 to 6 hours  *Treatment time:* not reported  *Number of sessions:*  Mean: 1 session/patient  Range: 1 to 4 sessions / patient  EMR  *Technique:* EMR with ligation, or cap and snare  *Injection:* none  *Number of treatments:* not reported  PDT + EMR Group  Details as above.  *Co-interventions:*  OM 40 mg IV twice daily or Pantoprazole 40 mg IV twice daily | *Outcomes:*  CR of HGD  Recurrence of HGD  Progression to cancer  *Adverse events:* | *Outcomes:*  CR of dysplasia …  … at 1 month (after 1 treatment session):  -All patients: 39/43 patients (91%)  -PDT Group: 26/27 patients (96%)  -EMR Group: 13/14 patients (93%)  -PDT + EMR Group: 2/3 patients (67%)  … at 38 months (mean) (after 1 to 4 sessions)  -All patients: 29/35 patients (83%)  Recurrence of HGD at 38 months (mean): 4/35 patients (11%)  Progression to cancer at 38 months (mean): 2/35 patients (6%)  *Adverse events:*  PDT Group  Vomiting, severe: 1/27 patients (4%)  Nausea: 14/27 patients (52%)  EMR Group | 4 |
| Hage M, et al. (2004)[13] | BE  BE+LGD | RCT  Prospective  PDT vs. APC  *Countries:* Netherlands  *Length of follow-up:* 24 months | Number of patients: 40  (PDT100 Group: 13 patients; PDT20+100 Group: 13 patients; APC Group: 14 patients)  PDT100 Group:  *Gender:*  Male: 10  Female: 3  *Age:*  Median: 57 yrs  Range: 52 to 72 yrs  PDT20+100 Group:  *Gender:*  Male: 10  Female: 3  *Age:*  Median: 61 yrs  Range: 57 to 69 yrs  APC Group:  *Gender:*  Male: 11  Female: 3  *Age:*  Median: 60 yrs  Range: 41 to 69 yrs  *Prior treatments:*  PPI, unspecified  *Length of Barrett’s:*  Median: 3 cm  Range: 2 to 5 cm  *Inclusion criteria:* none notable  *Exclusion criteria:*  Acute porphyria; pregnancy; intolerance to endoscopy; inter-current diseases with an adverse impact on survival | PDT100 Group:  *Drug:* 5-ALA  *Dose:* 60 mg/kg  *Route of administration:* oral  *Light source*: diode laser @ 633 nm  *Light dose:* 100 J/cm^2^  *Time to photoactivation:* 4 hours post ALA  *Treatment time:* not reported  *Number of sessions:* not reported  PDT20+100 Group:  *Drug:* 5-ALA  *Dose:* 60 mg/kg  *Route of administration:* oral  *Light source*: diode laser @ 633 nm  *Light dose:* 20 J/cm^2^ one hour post ALA + 100 J/cm^2^ 4 hours post ALA  *Time to photoactivation:* 4 hours post ALA  *Treatment time:* not reported  *Number of sessions:* not reported  APC Group:  *Gas flow:* 2L/minute  *Power:* 65 watts  *Number of sessions:* 2  2/3 of the lesion ablated in the 1st session and the rest in the second  *Co-interventions:*  OM 40mg/day | *Outcomes:*  CR of BE (assessed *endoscopically*)  CR of BE (assessed *histologically* through 4 quadrant biopsies every 2 cm)  *Adverse events* | *Outcomes:*  CR of BE by endoscopy at 6 weeks:  -PDT100 Group: 1/13 patients (8%)  -PDT20+100 Group: 5/13 patients (38%)  -APC Group: 7/14 patients (50%)  (PDT100 vs. PDT20+100: p<0.005)  (PDT20+100 vs. APC: not significant)  (PDT100 vs. APC: p<0.05)  CR of BE – histological at 6 weeks:  -PDT100 Group: 1/13 patients (8%)  -PDT20+100 Group: 4/13 patients (31%)  -APC Group: 5/14 patients (36%)  (no significant differences)  *Adverse events:*  PDT Groups  Pain during treatments: 23/26 patients (89%)  Odynophagia: 24/26 patients (92%)  Fever: 8/26 patients (31%)  Nausea/vomiting: 7/26 patients (27%)  Sudden death (presumably from cardiac arrhythmia): 1/26 patients (4%)  Strictures: 0/26 patients (0%)  Elevated liver enzymes: 20/26 patients (77%)  Buried glands: 1/26 patients (4%)  APC Group  Pain during treatments: 5/14 patients (36%)  Odynophagia: 12/14 patients (86%)  Fever: 2/14 patients (14%)  Nausea/vomiting: 0/14 patients (0%)  Sudden death (presumably from cardiac arrhythmia): 0/14 patients (0%)  Strictures: 1/14 patients (7%)  Elevated liver enzymes: 0/14 patients (0%)  Buried glands: 7/14 patients (50%) | 1 |
| Zoepf T, et al. (2003)[16] | BE + HGD  BE + LGD | RCT  Single centre  Prospective  PDT vs. APC  *Countries:* Germany  *Length of follow-up*:  PDT  Median: 27 months Range: 12 to 42 months  APC  Median: 24 months  Range: 4 to 46 months | *Number of patients:* 20  (PDT Group: 10 patients; APC Group: 10 patients)  *Gender:* not reported  *Age:*  Mean: 68 yrs  Range 44 to 77 yrs  *Prior treatments:* none reported  *Length of Barrett’s:*  PDT  Mean: 3.5 cm  Range: 3 to 12 cm  APC  Mean: 4.0 cm  Range: 3 to 7 cm  *Inclusion criteria:* none notable  *Exclusion criteria:* none notable | PDT vs. APC  PDT  *Drug:* 5-ALA  *Dose:* 60 mg/kg  *Route of administration:* oral  *Time to photoactivation:* not reported  *Light source:* diode laser @ non-reported wavelength  *Light dose:* 150J/cm^2^  *Treatment time:* not reported  *Number of sessions / patient:*  Mean: 2 sessions / patient  Range: 1 to 5 sessions / patient  APC  *Power:* 70 watts  *Gas flow:* not reported  *Treatment time:* not reported  *Number of sessions / patient:*  Mean: 4 sessions / patient  Range: 2 to 9 sessions / patient    *Co-interventions:* none reported | *Outcomes:*  Reduction in length of BE  *Adverse events* | *Outcomes:*  Reduction in length of BE “after treatment”:  PDT  Mean: 90%  Range: 0 to 100%  APC  Mean 90%  Range: 50 to 100%  *Adverse events:*  PDT  Nausea / vomiting: 10/10 patients (100%)  Dysphagia, transient 4/10 patients (40%)  Photosensitivity: 0/10 patients (0%)  Mediastinal emphysema: 0/10 patients (0%)  APC  Nausea / vomiting: 0/10 patients (0%)  Dysphagia, transient: 3/10 patients (30%)  Photosensitivity: 0/10 patients (0%)  Mediastinal emphysema: 1/10 patients (10%) | 1 |
| *Non-comparative studies* | | | | | | | |
| Barr H, et al. (1996)[34] | BE+HGD | Case series  Single-centre  *Countries:* UK  *Length of follow-up:*  Range: 26 to 44 months | *Number of patients:* 5  *Gender:*  Male: 3  Female: 2  *Age:*  Median: 74 years  Range: 56 to 81 years  *Length of Barrett’s:* not reported  *Prior treatments:* none reported  *Inclusion criteria:* none notable  *Exclusion criteria:* none notable | PDT  *Drug:* ALA  *Dose:* 60 mg/kg  *Route of administration:* oral  *Light source*: laser @ 630nm  *Light dose:* 90 to 150 J/cm^2^  *Time to photoactivation:* 4 *Treatment time:* not reported  *Number of sessions:*  1 session/patient  *Co-interventions:*  OM 40mg/day | *Outcomes:*  Partial response of dysplasia (defined as any squamous re-epithelialization) (method of assessment not reported)  *Adverse events:* | *Outcomes:*  Partial response of dysplasia post treatment: 5/5 patients (100%)  *Adverse events:*  Buried glands: 2/5 patients (40%) | 4 |
| Gossner L, et al. (1998)[35]*  * Information extracted for BE or HGD patients only | BE + HGD | Case series  Single centre  *Countries:* Germany  *Length of follow-up:*  Mean: 5.4 months  Range: 1 to 11 months | *Number of patients:* 10  *Gender:*  Male: 9  Female: 1  *Age:*  Mean: 69.6 yrs ± 7.91 yrs  *Prior treatments:* none reported  *Length of Barrett’s:*  Mean: 5.1 cm  Range: 0.5 to 10 cm  *Length of dysplasia:*  Range: 27 to 36 cm  *Inclusion criteria:*  Severe dysplasia or early EAC  Ineligible for surgery  *Exclusion criteria:* none notable | PDT  *Drug:* 5-ALA  *Dose:* 60 mg/kg  *Route of administration:* oral  *Light source*: dye laser (KTP/YAG) @ 635nm  *Light dose:* 150 J/cm^2^ @ 100mW/cm^2^  *Time to photoactivation:* 4 to 6 hours  *Treatment time:* not reported  *Number of sessions:*  Mean: 2.2 sessions / patient  *Co-interventions:*  OM 20 to 40 mg post treatment | *Outcomes:*  CR of BE (assessed through endoscopy with 4 quadrant biopsy over the “whole length” of BE)  Partial response of BE  CR of HGD  *Adverse events:* No BE or HGD specific information available. | *Outcomes:*  CR of BE at 5.4 months (mean): 0/10 patients (0%)  Partial response of BE: 10/10 patients (100%)  CR of dysplasia at 5.4 months (mean): 10/10 patients (100%) | 4 |
| Gossner L, et al. (1999)[36] | HGD | Case report  Single centre  Prospective  *Countries:* Germany  *Length of follow-up:*  Mean: 10.5 months  Range: 10 to 11 months | *Number of patients*: 2  *Gender:*  Male: 1  Female: 1  *Age:*  Range: 48 to 79 yrs  *Prior treatments:* not reported  *Length of Barrett’s:* not reported  *Inclusion criteria:*  Ineligible for or refused surgery  *Exclusion criteria:* none notable | PDT  *Drug:* 5-ALA  *Dose:* 60 mg/kg  *Route of administration:* oral  *Light source*: KTP:YAG laser @ 635nm  *Light dose:* 150J/cm^2^  *Time to photoactivation:* not reported  *Treatment time:* not reported  *Number of sessions:* not reported  *Co-interventions:* none reported | *Outcomes:*  CR of HGD  Survival  *Adverse events*: | *Outcomes:*  CR of HGD at 2 days: 2/2 patients (100%)  Survival at 10.5 months (mean): 2/2 patients (100%)  Adverse events:  Perforation: 0/2 patients (0%)  Stricture: 0/2 patients (0%) | 4 |
| Kashtan H, et al. (2002)[37] | BE + LGD (7 patients)  BE + HGD (1 patient) | Clinical trial  Single centre  Prospective  *Countries:* Israel  *Length of follow-up:* Range: 18 to 30 months | *Number of patients:* 8  *Gender:*  Male: 7  Female: 1  *Age:*  Mean: 70.6 yrs  Range: 52 to 84 yrs  *Prior treatments:* none reported  *Length of Barrett’s:* not reported  *Inclusion criteria:* none notable  *Exclusion criteria:*  Photosensitivity, impaired liver function tests; porphyria | PDT  *Drug:* 5 ALA  *Dose:* 60 mg/kg  *Route of administration:* oral  *Light source*: xenon lamp @ 580 to 720nm and 1250 to 1600nm  *Light dose:* 100J/cm^2^  *Time to photoactivation:* not reported  *Treatment time:* not recorded  *Number of sessions:* 1  *Co-interventions:* none reported | *Outcomes:*  CR of BE  CR of HGD  CR of LGD  Progression to cancer  *Adverse events* | *Outcomes:*  CR of BE at 18 to 30 months: 3/8 patients (38%)  CR of HGD at 18 to 30 months: 0/1 patients (0%)  CR of LGD at 18 to 30 months: 4/7 patients (57%)  Progression to cancer at 18 to 30 months: 0/8 patients (0%)  *Adverse events:*  Photosensitivity: 6/8 patients (75%)  Nausea and vomiting: 4/8 patients (50%) | 4 |
| Mackenzie G, et al. (2005)[22] | BE + HGD | Case series  Single centre  *Countries:* UK  *Length of follow-up:*  Mean: 15.8 months  Range: 1 to 72 months | *Number of patients:* 51*  *Gender:* not reported  *Age*: not reported  Group A  *Number of patients:* 21 patients  *Gender:* not reported  *Age:* not reported  Group B  *Number of patients:* 12 patients  *Gender:* not reported  *Age:* not reported  Group C  *Number of patients:* 16 patients  *Gender:* not reported  *Age:* not reported  *Prior treatments: none reported*  *Length of Barrett’s:* not reported  *Inclusion criteria:* none notable  *Exclusion criteria:* none notable  * 3 patients unaccounted for. | PDT  *Drug:* ALA  *Route of administration:* oral  *Light source*: not reported  *Time to photoactivation:* not reported  *Treatment time:* not reported  *Number of sessions:*  Mean: 1.74 sessions / patient  Group A  *Dose:* 60 mg/kg  *Light dose:* 1000J/ cm^2^  Other details as above  Group B  *Dose:* 60 mg/kg  *Light dose:* 500 to 750J/cm^2^  Other details as above  Group C  *Dose:* 30 mg/kg  *Light dose:* 1000J/ cm^2^  Other details as above  *Co-interventions:* none reported | *Outcomes:*  CR of HGD:  *Adverse events* | *Outcomes:*  CR of dysplasia at 15.8 months (mean):  Group A: 16/21 patients (76%)  Group B: 2/12 patients (17%)  Group C: 5/16 patient (31%)  All patients: 23/49 patients (47%)  *Adverse events:*  Strictures and photosensitivity: 0/51 patients (0%)  Hypotension: 3/51 patients (6%)  GI bleed requiring transfusion: 1/51 patients (2%) | 4 |
| Mackenzie GD, et al. (2008)[38] | BE + HGD | RCT  Single centre  Prospective  Porfimer sodium PDT vs 5-ALA PDT  *Countries:* UK  *Length of follow-up*: not reported | *Number of patients:* 32  (Porfimer sodium PDT Group: 16 patients; ALA PDT Group: 16 patients)  *Gender*: not reported  *Age:* not reported  *Prior treatments:*  HGD nodules removed by EMR  *Length of Barrett’s:* not reported  *Inclusion criteria:*  Residual HGD after EMR  *Exclusion criteria:* none notable | PDT  Porfimer sodium PDT Group  *Drug:* Porfimer sodium  *Dose:* not reported  *Route of administration:* not reported  *Light source*: not reported  *Light dose:* not reported  *Time to photoactivation:* not reported  *Treatment time:* not reported  *Number of sessions:* not reported  ALA PDT Group  *Drug:* 5-ALA  *Dose:* 60 mg/kg  *Route of administration:* oral  *Light source*: red laser  *Light dose:* 1178J/cm  *Time to photoactivation:* not reported  *Treatment time:* not reported  *Number of sessions:*  Mean:: 1.16 sessions  Range: 1 to 2 sessions  *Co-interventions:* none reported | *Outcomes:*  CR of HGD (assessed through endoscopy with 4 quadrant biopsies every 2 cm)  *Adverse events* | *Outcomes:*  CR of dysplasia at unknown follow-up:  -Porfimer sodium PDT: 9/14 patients (64%)  -5 ALA PDT: 14/14 patients (100%)  (p<0.05)  *Adverse events:*  Porfimer sodium PDT Group  Strictures 6/16 patients (38%)  Photosensitivity: 7/16 patients (44%)  5 ALA PDT Group  Strictures: 1/16 patients (6%)  Photosensitivity: 0/16 patients (0%)  (p<0.05, porfimer sodium vs. ALA) | 4 |
| Macrae FA, et al. (2004)[39] | BE + HGD | Case series  Retrospective  *Countries:* Australia  *Length of follow-up:*  Range: 5 to 98 months | *Number of patients:* 8  *Gender:* not reported  *Age:* not reported  *Prior treatments:* not reported  *Length of Barrett’s:* not reported  *Inclusion criteria:* none notable  *Exclusion criteria:* none notable | PDT  *Drug:* 5-ALA  *Dose:* 60 mg/kg in 3 divided doses  *Route of administration:* oral  *Light source*: KTP laser @ 628nm  *Light dose:* 150J/cm^2^  *Time to photoactivation:* 18 hours from 1st dose  *Treatment time:* not reported  *Number of sessions:* 1 session / patient  *Co-interventions:* none reported | *Outcomes:*  CR of HGD  Progression to cancer  Survival  *Adverse events* | *Outcomes:*  CR of dysplasia at 5 to 98 months: 3/8 patients (37.5%)  Progression to cancer at 5 to 98 months: 1/8 patients (12.5%)  Survival at 5 to 98 months: 8/8 patients (100%)  *Adverse events:*  Strictures: 1/8 patients (12.5%)  Photosensitivity: common | 4 |
| Mellidez JC, et al. (2005)[40] | BE + HGD | Case series  Single centre  Prospective  *Countries:* not reported  *Length of follow-up:* not reported | *Number of patients:* 13  (PDT Red Light: 8 patients; PDT Green light: 4 patients)  Red light group  *Gender:* not reported  *Age:* not reported  Green light group  *Gender:* not reported  *Age:* not reported  1 additional patient lost to follow-up, treatment allocation unknown.  *Prior treatments:* none reported  *Length of Barrett’s:* not reported  *Inclusion criteria:* none notable  *Exclusion criteria:* none notable | PDT  *Drug:* ALA  *Dose:* 60 mg/kg  *Route of administration:* oral  *Light source*: red light laser or green light laser  *Light dose:* not reported  *Time to photoactivation:* 4 hours  *Treatment time:* not reported  *Number of sessions:* not reported  *Co-interventions:*  Preceded by EMR | *Outcomes:*  CR of HGD  -Red light  -Green light  Reduction in area of columnar mucosa  -Red light  -Green light  *Adverse events* | *Outcomes:*  CR of dysplasia at unknown follow-up:  -Red light: 7/8 patients (87.5%)  -Green light: 2/4 patients (50%)  Difference is statistically significant  Reduction in area of columnar mucosa at unknown follow-up:  -Red light: 59%  -Green light: 7%  *Adverse events:*  GI bleed: 1/13 patients (8%) | 4 |
| **HpD 1.5mg/kg administered intravenously** | | | | | | | |
| *Comparative studies* | | | | | | | |
| None | | | | | | | |
| *Non-comparative studies* | | | | | | | |
| Laukka MA, et al. (1995)[41] | BE + LGD (4 patients)  BE + HGD (1 patient) | Case series  Single centre  *Countries:* US  *Length of follow-up:*  Range: 2 to 12 months | *Number of patients:* 5  *Gender:*  Male: 4  Female: 1  *Age:*  Median: 69 yrs  Range: 56 to 80 yrs  *Prior treatments:* none reported  *Length of Barrett’s:*  Mean: 9.8 cm  Range: 7 to 13 cm  *Inclusion criteria:* none notable  *Exclusion criteria:*  Pregnancy; lactation; allergy to OM, or contraindications to endoscopy | PDT  *Drug:* HpD  *Dose:* 1.5 mg/kg  *Route of administration:* IV  *Light source*: argon pumped dye laser @ 630nm  *Light dose:* 175J/cm^2^  *Time to photoactivation:* not reported  *Treatment time:* 6 minutes / 2cm segment  *Number of sessions:* 1 session / patient  *Co-interventions:*  OM 20 mg for 6 months | *Outcomes:*  CR of BE (assessed through endoscopy with 4 quadrant biopsies every 2 cm)  CR of HGD  Mean reduction in length of BE  *Adverse events* | *Outcomes:*  CR of BE at 2 months: 0/5 patients (0%)  CR of HGD at 2 to 12 months: 1/1 patient (100%)  Mean reduction in length of BE at 2 months: 24% (range: 10 to 50%)  *Adverse events:*  Nausea: 2/5 patients (40%)  Anorexia: 2/5 patients (40%)  Photosensitivity: 2/5 patients (40 %)  Buried glands: observed | 4 |
| Wang KK, et al. (1997)[42] | BE (23 patients)  BE + LGD (32 patients)  BE + HGD (9 patients) | RCT  Single centre  Prospective  *Countries:* US  *Length of follow-up:* Mean: 25 months ± 2 months | *Number of patients:* 75  (PDT Group: 55 patients; Control Group: 20 patients)  *Gender:*  Male: 61  Female: 14  *Age:*  Mean: 61 yrs ± 1 yr  PDT Group  *Number of patients:* 55  *Gender:* not reported  *Age:* not reported  Control Group  *Number of patients:* 20  *Gender:* not reported  *Age:* not reported  *Prior treatments:* none reported  *Length of Barrett’s:* not reported  *Inclusion criteria:* none notable  *Exclusion criteria:* none notable | PDT  PDT Group  *Drug:* HpD  *Dose:* 1.5 to 2.0 mg/kg  *Route of administration:* IV  *Light source*: argon pumped dye laser @ 630nm  *Light dose:* 175 to 200 J/cm^2^  *Time to photoactivation:* 48 hours  *Treatment time:* not reported  *Number of sessions:*  Mean:: 1 session / patient  Control Group  not reported  *Co-interventions:* none reported | *Outcomes:*  CR of BE (assessed through endoscopy with 4 quadrant biopsy every 1 cm)  Reduction in treated BE length  *Adverse events* | *Outcomes:*  CR of BE at unknown follow-up:  -PDT Group: 7/55 patients (13%)  -Control Group: 0/20 patients (0%)  (p<0.05)  Reduction in treated BE length at unknown follow-up:  -PDT Group: 7±1 cm to 4 ±1 cm  -Control Group: 6±1 cm to 6±1 cm  *Adverse events:*  Photosensitivity: common  Odynophagia: common  Strictures: 0/54 patients (0%) | 4 |
| Wang KK, et al. (1999)[43]  * Information extracted for BE or HGD patients only | BE (9 patients)  BE + LGD (30 patients)  BE + HGD (11 patients) | Clinical trial  Single centre  Prospective  *Countries:* US  *Length of follow-up:*  Mean: 24 months ± 3 months | *Number of patients:* 50  *Gender:*  not reported  *Age:* not reported  *Prior treatments:* none reported  *Length of Barrett’s:*  Mean 6cm ±1 cm  *Inclusion criteria:* none notable  *Exclusion criteria:* none notable | PDT  *Drug:* HpD  *Dose:* 1.75 to 4.0 mg/kg  *Route of administration:* IV  *Light source*: not reported  *Light dose:* 175 to 200J/cm^2^  *Time to photoactivation:* 48 hours  *Treatment time:* not reported  *Number of sessions:* 1 session / patient  *Co-interventions:*  OM 40 mg./ day for one month then 20 mg/day | *Outcomes:*  Progression to HGD from BE or LGD  Length of Barrett’s  *Adverse events:* none | *Outcomes:*  Progression to HGD from BE or LGD 24 months (mean): 4/39 patients (10%)  Length of Barrett’s:  -Pre-PDT: mean 6cm ± 1 cm  - at 3 months: mean 3 cm ± 1 cm | 4 |
| **mTHPC 0.15mg/kg administered intravenously** | | | | | | | |
| *Comparative studies* | | | | | | | |
| None | | | | | | | |
| *Non-comparative studies* | | | | | | | |
| Javaid B, et al. (2002)[44]*  * Information extracted for BE or HGD patients only | BE + HGD | Clinical trial  Single centre  Prospective  *Countries:* UK  *Length of follow-up:*  Mean: 12.8 months  Range: 4 to 27 months | *Number of patients:* 6  *Gender:* not reported  *Age:* not reported  *Prior treatments:* none reported  *Length of Barrett’s:*  Mean 6.6 cm  Range:1.2 to 13 cm  *Inclusion criteria:* none notable  *Exclusion criteria:* none notable | PDT  *Drug:* m-tetrahydroxyphenyl chlorin (mTHPC)  *Dose:* 0.15 mg/kg  *Route of administration:* IV  *Light source*: argon pumped dye laser @ 652nm (4 patients) and Xenon arc lamp @ 652±15 nm (2 patients)  *Light dose:* 8 to 20J/cm^2^  *Time to photoactivation:* 96 hours  *Treatment time:* not reported  *Number of sessions:*  Mean: 1.5 sessions / patient  Range: 1 to 3 sessions  *Co-interventions:*  PPI, unspecified | *Outcomes:*  CR of BE (assessed through endoscopy with 4 quadrant biopsies every 2 cm)  Partial response of BE (defined as any reduction in BE length <100%)  CR of HGD  Partial response of dysplasia  Progression to cancer  *Adverse events:* No BE or HGD specific information available. | *Outcomes:*  CR of BE at 4 weeks: 1/6 patients (17%)  Partial response of BE: 3/6 patients (50%)  CR of dysplasia at 4 weeks: 4/6 patients (67%)  Partial response of dysplasia at 4 weeks: 2/6 patients (33%)  Progression to cancer at 12.8 months (mean): 0/6 patients (0%) | 4 |
| Lovat LB, et al. (2005)[45]*  * Information extracted for BE or HGD patients only | BE + HGD | Case series  Single centre  *Countries:* UK  *Length of follow-up:*  Mean: 20.6 months  Range: 16 to 24 months  Red Light Group  Mean: 19.8 months  Range: 16 to 23 months  Green Light Group  Mean: 21.7 months  Range: 19 to 24 months | *Number of patients:* 7  *Gender:*  Male: 7  Female: 0  *Age:*  Range: 61 to 81 yrs  Red Light Group  *Number of patients:* 4 patients  *Gender:* not reported  *Age:* not reported  Green Light Group  *Number of patients:* 3 patients  *Gender:* not reported  *Age:* not reported  *Prior treatments:*  PDT (2 patients)  EMR (1 patient)  Laser (1 patient)  *Length of Barrett’s:*  Mean: 2 cm  Range: 1 to 4 cm  *Inclusion criteria:*  Ineligible for or refusing surgery  *Exclusion criteria:* none notable | PDT  *Drug:* m-tetrahydroxyphenyl chlorin (mTHPC)  *Dose:* 0.15 mg/kg  *Route of administration:* IV  *Time to photoactivation:* 3 days  *Treatment time:* not reported  *Number of sessions:* 1 session  Red Light Group  *Light source:* diode laser @ 652nm  *Light dose:* 75J/cm^2^  Other details as above  Green Light Group  *Light source:* copper vapour laser @ 511 nm  *Light dose:* 75J/cm^2^  Other details as above  *Co-interventions:* none reported | *Outcomes:*  CR of BE (assessed through endoscopy with 4 quadrant biopsies every 2 cm)  -Red Light Group  -Green Light Group  CR of HGD to BE  -Red Light Group  -Green Light Group  Progression to cancer  -Red Light Group  -Green Light Group  Mortality  -All cause  -EAC  *Adverse events:* No BE or HGD specific information available. | *Outcomes:*  CR of BE at 20.6 months (mean):  - Red Light Group: 0/4 patients (0%)  - Green Light Group: 0/3 patients (0%)  CR of dysplasia at 20.6 months (mean):  - Red Light Group: 3/4 patients (75%)  - Green Light Group: 0/3 patients (0%)  Progression to cancer at 20.6 months (mean):  - Red Light Group: 0/4 patients (0%)  - Green Light Group: 1/3 patients (33%)  Mortality at 20.6 months (mean):  -All cause: 2/7 patients (29%)  -EAC: 0/5 patients (0%) | 4 |
| **Porfimer sodium 2mg/kg administered intravenously** | | | | | | | |
| *Comparative studies* | | | | | | | |
| Ragunath K, et al. (2005)[15] | BE + HGD  BE + LGD | RCT  Single centre  Prospective  PDT vs. APC  *Countries*: UK  *Length of follow-up:* 12 months | Number of patients: 26  (PDT Group: 13 patients; APC Group: 13 patients)  PDT Group  *Gender:*  Male: 13  Female: 0  *Age:*  Mean: 58.1 yrs  Range 35 to 79 yrs  APC Group  *Gender:*  Male: 10  Female: 3  *Age:*  Mean: 64.9 yrs  Range: 41 to 86 yrs  *Prior treatments:* not reported  *Length of Barrett’s:*  PDT Group  Mean: 5.7 cm  Range: 3 to 9 cm  APC Group  Mean: 5.5 cm  Range: 3 to 9 cm  *Inclusion criteria:* none notable  *Exclusion criteria:*  Previous or current esophageal malignancy; previous esophagectomy; history of EMR or mucosal ablation treatment; predominantly “tongues” as opposed to circumferential BE; history of porphyria; pregnancy or lack of contraception | PDT vs. APC  PDT Group  *Drug:* porfimer sodium  *Dose*: 2 mg.kg  *Route of administration:* IV  *Time to photoactivation:* 48 hours  *Light source:* argon pump dye laser @630 nm  *Light dose:* 200 J/cm^2^  *Treatment time:* not recorded  *Number of sessions:* 1 session / patient  APC Group  *Gas flow:* 1.8L/minute  *Power:* 65 watts  *Treatment time:* not recorded  *Number of sessions:* 1 session / patient  *Co-interventions:*  Lansoprazole 60 mg/day during treatment then 30 mg/day | *Outcomes:*  CR of BE (assessed through endoscopy with 4 quadrant biopsy every 1 cm)  CR of HGD (assessed through endoscopy with 4 quadrant biopsy every 1 cm)  CR of LGD (assessed through endoscopy with 4 quadrant biopsy every 1 cm)  CR of dysplasia  Reduction in length of BE  Progression to cancer  *Adverse events* | *Outcomes:*  CR of BE:  PDT Group  - at 4 months: 2/13 patients (15%)  - at 12 months: 2/13 patients (15%)  APC Group  - at 4 months: 2/13 patients (15%)  - at 12 months: 0/9 patients (0%)  CR of HGD:  PDT Group  - at 4 months: 2/2 patients (100%)  - at 12 months: 2/2 patients (100%)  APC Group  - at 4 months: 1/1 patient (100%)  - at 12 months: 0/0 patients (0%)  CR of LGD:  PDT Group  - at 4 months: 8/11 patients (73%)  - at 12 months: 8/11 patients (73%)  APC Group  - at 4 months: 7/12 patients (58%)  - at 12 months: 6/9 patients (67%)  CR of dysplasia:  PDT Group  - at 4 months: 10/13 patients (77%)  - at 12 months: 10/13 patients (77%)  APC Group  - at 4 months: 8/13 patients (62%)  - at 12 months: 6/9 patients (67%)  (p=0.03)  Reduction in length of BE:  PDT Group  - at 4 months: 57% reduction  - at 12 months: 61% reduction  APC Group  - at 4 months: 65% reduction  - at 12 months: 56% reduction  Progression to cancer:  PDT Group  - at 4 months: 0/13 patients (0%)  - at 12 months: 1/13 patients (8%)  APC  - at 4 months: 0/13 patients (0%)  - at 12 months: 0/13 patients (0%)  *Adverse events:*  PDT Group  Strictures: 2/13 patients (15%)  Chest pain, odynophagia and fever: 0/13 patients (0%)  Photosensitivity: 2/13 patients (15%)  Buried glands: 1/13 patients (8%)  APC Group  Strictures: 3/13 patients (23%)  Chest pain, odynophagia and fever: 1/13 patients (8%)  Photosensitivity: 0/13 patients (0%)  Buried glands: 0/13 patients (0%) | 1 |
| *Non-comparative studies* | | | | | | | |
| Attila T, et al. (2005)[46] | BE +HGD | Case series  Single centre  Retrospective  *Countries:* not reported  *Length of follow-up:*  Mean:43.7 months  Range: 2 to 80 months | *Number of patients:* 19  *Gender:*  Male: 15  Female: 4  *Age:*  Mean: 66.4 yrs ± 7.5 yrs  *Prior treatments:*  None  *Length of Barrett’s:*  Mean: 5.1 cm ± 2.4 cm  *Inclusion criteria:* none notable  *Exclusion criteria:* none notable | PDT  *Drug:* Porfimer sodium  *Dose:* 2 mg/kg  *Route of administration:* IV  *Light source*: laser @630nm  *Light dose:* not reported  *Time to photoactivation:* 48 to 72 hours  *Treatment time:* not reported  *Number of sessions:* not reported  2nd PDT or APC done for residual lesions  *Co-interventions:* none reported | *Outcomes:*  CR of BE (assessed through endoscopy with 4 quadrant biopsies every 2cm)  Partial response of BE (defined as residual BE islands or tongues)    No response of BE (defined as unchanged length of Barrett’s)  Progression to cancer  *Adverse events* | *Outcomes:*  CR of BE:  - at 3 months (after 1 PDT session): 5/19 patients (26%)  - at 43.7 months (mean) (after PDT + additional therapy): 12/19 patients (63%)  Partial response of BE at 3 months (after 1 PDT session): 9/19 patients (47%)  No response of BE at 3 months (after 1 PDT session): 5/19 patients (26%)  Progression to cancer at 43.7 months (mean): 2/19 patients (10.5%)  *Adverse events:*  Strictures: 7/19 patients (36.8%) | 4 |
| Bronner M, et al. (2006)[47] | BE + HGD | RCT  Prospective  PDT with OM vs. OM  *Countries:* not reported  *Length of follow-up*: 5 yrs | *Number of patients:* 208  (PDT + OM Group: 138 patients; OM Group:70 patients)  PDT+OM Group  *Gender:* not reported  *Age:* not reported  OM Group  *Gender:* not reported  *Age:* not reported  *Prior treatments:* none reported  *Length of Barrett’s:* not reported  *Inclusion criteria:* none notable  *Exclusion criteria:* none notable | PDT  PDT + OM  *Drug:* porfimer sodium  *Dose:* 2 mg/kg  *Route of administration:* IV  *Light source*: 630nm laser  *Light dose:* not reported  *Time to photoactivation:* 40 to 50 hours  *Treatment time:* not reported  *Number of sessions:* up to 3 PDT sessions at least 90 days apart  OM 20 mg twice daily  OM  OM 20 mg twice daily  *Co-interventions:*  OM as above | *Outcomes:* none reported  *Adverse events* | *Outcomes:*  *Adverse events:*  Buried glands  PDT + OM Group:  31% of patients  1.2% of biopsies  OM Group:  33% of patients  2.2% of biopsies | 4 |
| Keeley SB, et al. (2007)[48]  * Information extracted for BE or HGD patients only | HGD | Case series  Single centre  Retrospective  *Countries:* US  *Length of follow-up:*  Mean: 28.1 months  Range: 1 to 81 months | *Number of patients:* 13  *Gender:* not reported  *Age:* not reported  *Prior treatments:* none reported  *Length of Barrett’s:* not reported  *Inclusion criteria:*  Ineligible for or refusal of surgery  *Exclusion criteria:* none notable | PDT  *Drug:* porfimer sodium  *Dose:* not reported  *Route of administration:* IV  *Light source*: red laser @630nm  *Light dose:* 300 to 400J/cm^2^  *Time to photoactivation:* 48 hours  *Treatment time:* not reported  *Number of sessions:* >1 (not reported  *Co-interventions:* none reported | *Outcomes:*  CR of HGD  Mortality  -Overall  -Disease related  Survival  -Overall  -Disease related  *Adverse events:* No BE or HGD specific information available. | *Outcomes:*  CR of HGD at 28.1 months (mean): 5/13 patients (38%)  Mortality at 28.1 months (mean):  -Overall: 4/10 patients (40%)  -Disease related: 0/6 patients (0%)  Survival at 28.1 months (mean):  -Overall: 6/10 patients (60%)  -Disease related: 6/6 patients (100%) | 4 |
| Mackenzie GD, et al. (2008)[38] | BE + HGD | RCT  Single centre  Prospective  Porfimer sodium PDT vs 5-ALA PDT  *Countries:* UK  *Length of follow-up*: not reported | *Number of patients:* 32  (Porfimer sodium PDT Group: 16 patients; ALA PDT Group: 16 patients)  *Gender*: not reported  *Age:* not reported  *Prior treatments:*  HGD nodules removed by EMR  *Length of Barrett’s:* not reported  *Inclusion criteria:*  Residual HGD after EMR  *Exclusion criteria:* none notable | PDT  Porfimer sodium PDT Group  *Drug:* Porfimer sodium  *Dose:* not reported  *Route of administration:* not reported  *Light source*: not reported  *Light dose:* not reported  *Time to photoactivation:* not reported  *Treatment time:* not reported  *Number of sessions:* not reported  ALA PDT Group  *Drug:* 5-ALA  *Dose:* 60 mg/kg  *Route of administration:* oral  *Light source*: red laser  *Light dose:* 1178J/cm  *Time to photoactivation:* not reported  *Treatment time:* not reported  *Number of sessions:*  Mean:: 1.16 sessions  Range: 1 to 2 sessions  *Co-interventions:* none reported | *Outcomes:*  CR of HGD (assessed through endoscopy with 4 quadrant biopsies every 2 cm)  *Adverse events* | *Outcomes:*  CR of dysplasia at unknown follow-up:  -Porfimer sodium PDT: 9/14 patients (64%)  -5 ALA PDT: 14/14 patients (100%)  (p<0.05)  *Adverse events:*  Porfimer sodium PDT Group  Strictures 6/16 patients (38%)  Photosensitivity: 7/16 patients (44%)  5 ALA PDT Group  Strictures: 1/16 patients (6%)  Photosensitivity: 0/16 patients (0%)  (p<0.05, porfimer sodium vs. ALA) |  |
| Overholt BF, et al.(2007)[49] | BE + HGD | RCT  Multicentre  Prospective  OM vs PDT + OM  *Countries:* US, UK, Canada  *Length of follow-up:*  PDT + OM Group  Mean 332 days  Range: 48 to 1044 days  OM Group  Mean: 357 days  Range: 63 to 1092 days | Number of patients: 208  (PDT+OM Group: 138 patients; OM Group: 70 patients)  PDT + OM Group  *Gender:*  Male: 117  Female: 21  *Age:*  Mean: 66 yrs ± 11 yrs  OM Group  *Gender:*  Male: 59  Female: 11  *Age:*  Mean: 67 yrs ± 11 yrs  *Inclusion criteria:* none notable  *Exclusion criteria:*  Cancer other than non-melanoma skin cancer within the last 5 yrs; prior PDT to esophagus; strictures unresponsive to dilation; esophageal ulcers > 1 cm; porphyria; varices; pregnancy | OM vs. PDT + OM  PDT + OM  20 mg OM twice daily  *Drug:* Porfimer sodium  *Dosage:* 2mg/kg  *Route of administration:* IV  *Time to photoactivation:* 40 to 50 hrs  *Light source:* 630 nm KTP dye laser  *Total light dose:* 130 J/cm of diffuser length  *Treatment time:* Not reported  *Number of treatments:*  Mean 2 sessions / patient  Range: 1 to 3 patients  Maximum of 3 treatments at least 3 months apart over 3 years  OM Group  20 mg OM twice daily  *Co-interventions:* none reported | *Outcomes:*  Cumulative proportion of patients ever having a CR of HGD (assessed through endoscopy with 4 quadrant biopsy every 2 cm)  Cumulative probability of maintaining CR of HGD: (K-M analysis)  Progression to cancer  *Adverse events* | *Outcomes:*  Cumulative proportion of patients ever having a CR of HGD:  PDT + OM Group  - at 6 months: 73/138 patients (53%)  - at 12 months: 78/138 patients (71%)  - at 18 months 104/138 patients (75%)  - at 24 months: 106/138 patients (77%)  OM Group  - at 6 months: 18/70 patients (26%)  - at 12 months: 21/70 patients (30%)  - at 18 months: 25/70 patients (36%)  - at 24 months: 27/70 patients (39%)  Statistically significant difference between groups (p < 0.0001)  Cumulative probability of maintaining CR of HGD:  PDT + OM Group:  - at 6 months: 0.76  - at 12 months: 0.61  - at 18 months: 0.54  - at 24 months: 0.54  - at 5 yrs: 0.48 (p<0.001 vs. OM)  OM Group:  - at 6 months: 0.35  - at 12 months: 0.16  - at 18 months: 0.16  - at 24 months: 0.13  - at 5 yrs: 0.04 (p<0.001 vs. PDT +OM)  Progression to cancer at 5 years:  -PDT+ OM Group: 18/138 patients (13%)  -OM Group: 20/70 patients (29%)  (p<0.05)  *Adverse events:*  PDT + OM Group  Photosensitivity: 69%  Strictures: 36%  Hiccups: 10%  Vomiting: 32 %  Nausea: 11%  Chest pain, non-random: 20%  Fever: 20%  Dysphagia: 19%  Constipation: 13%  Dehydration 12%  OM Group: none reported | 4 |
| Overholt BF, et al. (2003)[50] *  * Information extracted for BE or HGD patients only | BE + HGD (80 patients)  BE + LGD (14 patients) | Clinical trial  Single centre  Prospective  *Countries:* US  *Length of follow-up:* Mean: 50.7 months  Range: 2 to 122 months | *Number of patients*: 94  *Gender:*  Male: 74  Female: 10  *Age:*  Mean: 64.9 ± 10.0 yrs  *Prior treatments:* none reported  *Length of Barrett’s:* not reported  *Inclusion criteria:*  Ineligible for surgery  *Exclusion criteria:* none notable | PDT  *Drug:* Porfimer sodium  *Dose:* 2 mg/kg  *Route of administration:* IV  *Light source*: argon pumped dye laser @ 630 nm  *Light dose:* 100-300 J/cm^2^  *Time to photoactivation:* 48 hours  *Treatment time:* not recorded  *Number of sessions:*  Median: 1.4 sessions  Range: 1 to 3 sessions  *Co-interventions:*  OM 20 mg twice daily  Nd:YAG ablation of residual BE <1.3 cm offered after 3 months  Most patients received Nd:YAG treatment off protocol | *Outcomes:*  CR of BE (assessed through endoscopy with 4 quadrant biopsies every 2 cm)  - From HGD  - From LGD  CR of HGD  CR of LGD  Cumulative probability of maintaining CR given CR*  Progression to cancer at 50.7 months (mean)  - From HGD  - From LGD  Survival at 50.7 months  - From HGD  - From LGD  *Adverse events:* No BE or HGD specific information available.  *extracted from a K-M survival curve | *Outcomes:*  CR of BE at 3 months:  - All patients: 53/94 patients (56%)  - From HGD: 43/80 patients (54%)  - From LGD: 10/14 patients (71%)  CR of HGD at 3 months: 62/80 patients: (78%)  CR of LGD at 3 months: 13/14 patients (93%)  Cumulative probability of maintaining CR given CR at 50.7 months (mean):  - HGD: approximately 75%  - LGD: approximately 43%  Progression to cancer at 50.7 months (mean):  - From HGD: 2/80 patients (2.5%)  - From LGD: 0/14 patients (0%)  Survival at 50.7 months (mean): 86/94 patients (91%)  - From HGD: 73/80 patients (91%)  - From LGD: 13/14 patients (93%) | 4 |
| Overholt BF (1997)[51] | BE + HGD | Case series  Single centre  *Countries:* US  *Length of follow-up:* not reported | *Number of patients:* 11  *Gender:*  Male: 9  Female: 2  *Age:*  Mean: 61.9 yrs  Range: 42 to 79 yrs  *Prior treatments:* not reported  *Length of Barrett’s:* not reported  *Inclusion criteria:* none notable  *Exclusion criteria:* none notable | PDT  *Drug:* Porfimer sodium  *Dose:* 2 mg/kg  *Route of administration:* IV  *Light source*: argon pumped dye laser @ 630nm  *Light dose:* 250 J/cm^2^  *Time to photoactivation:* 48 hours  *Treatment time:* not reported  *Number of sessions:* not reported  *Co-interventions:*  OM 20 mg twice daily | *Outcomes:* not reported  *Adverse events* | *Outcomes:*  *Adverse events:*  Atrial fibrillation, transient: 0/11 patients (0%)  Pleural effusion, small with no symptoms: 10/14 patients (71%) | 4 |
| Weiss AA, et al. (2006)[52] *  * Information extracted for BE or HGD patients only | BE + HGD | Case series  Single centre  *Countries:* Canada  *Length of follow-up:*  Mean: 21 months  Range: 3 to 55 months | *Number of patients:* 13  *Gender:*  Male: 12  Female: 1  *Age:*  Mean: 71.6 yrs ± 10.2 yrs  *Prior treatments:* none reported  *Length of Barrett’s:*  Mean: 5.7 cm  4/13 patients < 3 cm  9/12 patients ≥ 3 cm  *Inclusion criteria:*  Biopsy proven BE + HGD  Ineligible for or refusing surgery  *Exclusion criteria:* none notable | PDT  *Drug:* Porfimer sodium  *Dose:* 2 mg/kg  *Route of administration:* IV  *Light source*: KTP dye laser @ 630 nm  *Light dose:* 130J/cm^2^  *Time to photoactivation:* 48 hours  *Treatment time:* not reported  *Number of sessions:* not reported  *Co-interventions:*  PPI, unspecified | *Outcomes:*  CR of BE  CR of HGD  Partial response of BE  Progression to cancer  *Adverse events:* No BE or HGD specific information available. | *Outcomes:*  CR of BE at 21 months (mean): 4/13 patients (31%)  CR of dysplasia at 21 months (mean): 4/13 patients (31%)  Partial response of BE: 8/13 patients (62%)  Progression to cancer at 21 months (mean): 4/13 patients (31%) | 4 |
| Wolfsen HC, et al. (2004)[53] *  * Information extracted for BE or HGD patients only | BE + HGD | Case series  Single centre  Retrospective  *Countries:* US  *Length of follow-up:* 2 years | *Number of patients:* 69  *Gender:*  Male: 54  Female: 15  *Age:*  Median: 72  *Prior treatments:*  Long standing BE surveillance (55 patients)  *Length of Barrett’s:*  Median: 5 cm  *Inclusion criteria:* none notable  *Exclusion criteria:* none notable | PDT  *Drug:* Porfimer sodium  *Dose:* 2mg/kg  *Route of administration:* IV  *Light source*: diode laser @ unreported wavelength  *Light dose:* 150 to 225J/cm^2^  *Time to photoactivation:* 48 hours  *Treatment time:* not reported  *Number of sessions:* not reported  *Co-interventions:*  OM or esomeprazole, 80 to 120 mg/day | *Outcomes:*  CR of BE from HGD (assessed through endoscopy with 4 quadrant biopsies every 1 cm)  *Adverse events:* No BE or HGD specific information available. | *Outcomes:*  CR of BE from HGD at 6 weeks: 36/69 patients (52%) | 4 |
| Yachimski P, et al. (2008)[54] *  * Information extracted for BE or HGD patients only | BE + HGD | Case series  Single centre  Retrospective  *Countries:* US  *Length of follow-up:* not reported | *Number of patients:* 59  *Gender:* not reported  *Age:* not reported  *Prior treatments:* none reported  *Length of Barrett’s:* (among 116 patients):  Mean: 6.0 cm ± 3.3 cm  *Inclusion criteria:* none notable  *Exclusion criteria:* none notable | PDT  *Drug:* porfimer sodium  *Dose:* 2 mg/kg  *Route of administration:* IV  *Light source*: laser @ 630nm  *Light dose:* 150 J/cm^2^  *Time to photoactivation:* 48 hours  *Treatment time:* not reported  *Number of sessions:* not reported  *Co-interventions:*  OM 80mg/day | *Outcomes:*  None reported  *Adverse events* | *Outcomes:*  *Adverse events:*  Strictures: 8/59 patients (14%) | 4 |

### *Note:* ALA (aminolevulinic acid), APC (argon plasma coagulation), BE (Barrett’s esophagus), CR (complete response), EAC (esophageal adenocarcinoma), EMR (endoscopic mucosal resection), GI (gastrointestinal), HGD (high grade dysplasia), HpD (hematoporphyrin derivative), IV (intravenous), KTP (potassium titanyl phosphate laser), LGD (low grade dysplasia), mTHPC (meta-tetrahydroxyphenylchlorin), Nd:YAG (neodymium doped yttrium aluminum garnet laser), OM (omeprazole), PDT (photodynamic therapy), PPI (proton pump inhibitor), RCT (randomized controlled trial)
