# Supplementary material for: Endoscopic treatments for Barrett's esophagus: a systematic review of safety and effectiveness compared to esophagectomy
Source: BMC Gastroenterol. 2010 Sep 27;10:111. doi: 10.1186/1471-230X-10-111 (PMC2955687; doi:10.1186/1471-230X-10-111)
Supplement: Additional file 2 — Studies of argon plasma coagulation (APC) for Barrett's esophagus with/without dysplasia. Details of study and patient characteristics, outcomes and study quality of the included studies of APC for BE with/without dysplasia are presented in Additional file 2. [file 1471-230X-10-111-S2.DOC]

| **Study authors (year published)**  Additional file 2. Studies of argon plasma coagulation (APC) for Barrett's esophagus with/without dysplasia | | **Cancer / Cell Type** | **Study Design** | **Patients** | **Intervention** | | **Outcome Measures** | | | **Findings** | **Study quality** |
| --- | --- | --- | --- | --- | --- | --- | --- | --- | --- | --- | --- |
| *Comparative studies* | | | | | | | | | | | |
| Dulai GS, et al. (2005)[17] | | BE | RCT  Prospective  APC vs. MPEC  *Countries:* US  *Length of follow-up:* 1 to 1.5 months (after last session) | *Number of patients:* 52  (APC Group: 26 patients; MPEC Group: 26 patients)  APC Group  *Gender*  Male: 21  Female: 5  *Age:*  Mean: 58 yrs ± 11 yrs  MPEC Group  *Gender:*  Male: 23  Female: 3  *Age:*  Mean: 56 yrs ± 11 yrs  *Prior treatments:* none reported  *Length of Barrett’s:*  APC Group  Mean: 4.0 cm ± 1.5 cm  MPEC Group  Mean: 3.1cm ± 1.7 cm  *Inclusion criteria:* none notable  *Exclusion criteria:*  Severe active comorbid disease  Diagnosis of HGD or cancer  Prior antireflux surgery  Inability to discontinue NSAID therapy  Pregnancy, lactation or non-use of birth control measures  Allergy to PPI  Uncontrolled coagulopathy | | APC vs. MPEC  APC Group  *Gas flow*: 2L/minute  *Power:* 60 watts  *Treatment time*: not reported  *Number of sessions:*  Mean: 3.8 sessions ± 1.7 sessions  MPEC Group  *Probe:* not reported  *Power:* 16 watts  *Treatment time:* not reported  *Number of sessions:*  Mean: 2.9 sessions ± 1.5 sessions  *Co-interventions:*  Pantoprazole, dosing unspecified. | *Outcomes:*  CR of BE (assessed through endoscopy with 4 quadrant biopsies every 2 cm)  *Adverse events:* | | | *Outcomes:*  CR of BE at 1 to 1.5 months:  -APC Group: 21/26 patients (81%)  -MPEC Group: 23/26 patients (88%)  (p=0.68)  *Adverse events:*  APC Group  Chest pain, severe: 1/26 patients (4%)  MPEC Group  none | 1 |
| Hage M, et al. (2004)[13] | | BE  BE+LGD | RCT  Prospective  APC vs PDT  *Countries:* Netherlands  *Length of follow-up:* 24 months | Number of patients: 40  (APC Group: 14 patients; PDT100 Group: 13 patients; PDT20+100 Group: 13 patients)  APC Group:  *Gender:*  Male: 11  Female: 3  *Age:*  Median: 60 yrs  Range: 41 to 69 yrs  PDT100 Group:  *Gender:*  Male: 10  Female: 3  *Age:*  Median: 57 yrs  Range: 52 to 72 yrs  PDT20+100 Group:  *Gender:*  Male: 10  Female: 3  *Age:*  Median: 61 yrs  Range: 57 to 69 yrs  *Prior treatments:*  PPI, unspecified  *Length of Barrett’s:*  Median: 3 cm  Range: 2 to 5 cm  *Inclusion criteria:* none notable  *Exclusion criteria:*  Acute porphyria; pregnancy; intolerance to endoscopy; inter-current diseases with an adverse impact on survival | | APC Group:  *Gas flow:* 2L/minute  *Power:* 65 watts  *Number of sessions:* 2  2/3 of the lesion ablated in the 1st session and the rest in the second  PDT100 Group:  *Drug:* 5-ALA  *Dose:* 60 mg/kg  *Route of administration:* oral  *Light source*: diode laser @ 633 nm  *Light dose:* 100 J/cm^2^  *Time to photoactivation:* 4 hours post ALA  *Treatment time:* not reported  *Number of sessions:* not reported  PDT20+100 Group:  *Drug:* 5-ALA  *Dose:* 60 mg/kg  *Route of administration:* oral  *Light source*: diode laser @ 633 nm  *Light dose:* 20 J/cm^2^ one hour post ALA + 100 J/cm^2^ 4 hours post ALA  *Time to photoactivation:* 4 hours post ALA  *Treatment time:* not reported  *Number of sessions:* not reported  *Co-interventions:*  OM 40mg/day | *Outcomes:*  CR of BE (assessed *endoscopically*)  CR of BE (assessed *histologically* through 4 quadrant biopsies every 2 cm)  *Adverse events* | | | *Outcomes:*  CR of BE by endoscopy at 6 weeks:  -APC Group: 7/14 patients (50%)  (PDT100 vs. PDT20+100: p<0.005)  (PDT20+100 vs. APC: not significant)  -PDT100 Group: 1/13 patients (8%)  -PDT20+100 Group: 5/13 patients (38%)  (PDT100 vs. APC: = p<0.05)  CR of BE – histological at 6 weeks:  -PDT100 Group: 1/13 patients (8%)  -PDT20+100 Group: 4/13 patients (31%)  -APC Group: 5/14 patients (36%)  (no significant differences)  *Adverse events:*  APC Group  Pain during treatments: 5/14 patients (36%)  Odynophagia: 12/14 patients (86%)  Fever: 2/14 patients (14%)  Nausea/vomiting: 0/14 patients (0%)  Sudden death (presumably from cardiac arrhythmia): 0/14 patients (0%)  Strictures: 1/14 patients (7%)  Elevated liver enzymes: 0/14 patients (0%)  Buried glands: 7/14 patients (50%)  PDT Groups  Pain during treatments: 23/26 patients (89%)  Odynophagia: 24/26 patients (92%)  Fever: 8/26 patients (31%)  Nausea/vomiting: 7/26 patients (27%)  Sudden death (presumably from cardiac arrhythmia): 1/26 patients (4%)  Strictures: 0/26 patients (0%)  Elevated liver enzymes: 20/26 patients (77%)  Buried glands: 1/26 patients (4%) | 1 |
| Kelty CJ, et al. (2004)[14] | | BE | RCT  Single centre  Prospective  APC vs. PDT  *Countries:* UK  *Length of follow-up*: 24 months | *Number of patients:* 72  (APC Group: 37 patients; PDT Group:35 patients)  APC Group  *Gender:*  Male: 30  Female: 7  *Age:*  Median: 59 yrs  Range: 28 to 79 yrs  PDT Group  *Gender:*  Male: 28  Female: 7  *Age:*  Median: 61 yrs  Range: 33 to 83 yrs  *Prior treatments:* none reported  *Length of Barrett’s:*  APC Group  Median: 4 cm  Range: 2 to 8 cm  PDT Group  Median: 4 cm  Range: 2 to 15 cm  *Inclustion criteria:* none notable  *Exclusion criteria:* none notable | | APC vs PDT  APC Group  *Gas flow:*  2L/minute  *Power:* 65 watts  *Number of sessions:*  Median: 3 sessions  Range: 1 to 5 sessions  Max allowed: 5 sessions  PDT Group  *Drug:* 5-ALA  *Dose:* 30 mg/kg  *Route of administration:* oral  *Light source*: diode laser @ 633 nm  *Light dose:* 85 J/cm^2^  *Time to photoactivation:* 4 to 6 hours post ALA  *Treatment time:* not reported  *Number of sessions:*  Median: 5 sessions  Range: 1 to 5 sessions  Max allowed: 5 sessions  *Co-interventions:* none reported | *Outcomes:*  CR of BE (assessed through endoscopy and 4 quadrant biopsy every 2 cm)  Partial response of BE  Number of treatments to achieve CR of BE  *Adverse events* | | | *Outcomes:*  CR of BE at 4 weeks:  -APC Group: 33/34 patients (97%)  -PDT Group: 17/34 patients (50%)  Partial response of BE:  -APC Group: 1/34 patients (3%)  -PDT Group: 17/34 patients (50%)  Number of treatments to achieve CR of BE:  APC Group  Median: 3 treatments  Range: 1 to 5 treatments  PDT Group  Median: 2 treatments  Range: 1 to 4 treatments  *Adverse events:*  APC Group  Nausea / vomiting; photosensitivity; hypotension; chest pain; elevated liver enzymes, mild: 0/34 patients (0%)  Odynophagia: 32/34 patients (94%)  Dysphagia secondary to strictures: 1/34 patients (3%)  Buried glands (4 week follow-up): 7/33 patients (21%)PDT Group  Nausea / vomiting: 11/34 patients (32%)  Photosensitivity: 5/34 patients (15 %)  Hypotension: 2/34 patients (6%)  Chest pain: 1/34 patients (3%)  Odynophagia: 1/34 patients (3%)  Dysphagia secondary to strictures: 0/34 patients (0%)  Elevated liver enzymes, mild: 4/34 patients (12%)  Buried glands (4 week follow-up): 4/17 patients (24%) | 1 |
| Ragunath K, et al. (2005)[15] | | BE + HGD  BE + LGD | RCT  Single centre  Prospective  APC vs. PDT  *Countries*: UK  *Length of follow-up:* 12 months | Number of patients: 26  (APC Group: 13 patients; PDT Group: 13 patients)  APC Group  *Gender:*  Male: 10  Female: 3  *Age:*  Mean: 64.9 yrs  Range: 41 to 86 yrs  PDT Group  *Gender:*  Male: 13  Female: 0  *Age:*  Mean: 58.1 yrs  Range 35 to 79 yrs  *Prior treatments:* not reported  *Length of Barrett’s:*  APC Group  Mean: 5.5 cm  Range: 3 to 9 cm  PDT Group  Mean: 5.7 cm  Range: 3 to 9 cm  *Inclusion criteria:* none notable  *Exclusion criteria:*  Previous or current esophageal malignancy; previous esophagectomy; history of EMR or mucosal ablation treatment; predominantly “tongues” as opposed to circumferential BE; history of porphyria; pregnancy or lack of contraception | | APC vs. PDT  APC Group  *Gas flow:* 1.8L/minute  *Power:* 65 watts  *Treatment time:* not recorded  *Number of sessions:* 1 session / patient  PDT Group  *Drug:* porfimer sodium  *Dose*: 2 mg.kg  *Route of administration:* IV  *Time to photoactivation:* 48 hours  *Light source:* argon pump dye laser @630 nm  *Light dose:* 200 J/cm^2^  *Treatment time:* not recorded  *Number of sessions:* 1 session / patient  *Co-interventions:*  Lansoprazole 60 mg/day during treatment then 30 mg/day | *Outcomes:*  CR of BE (assessed through endoscopy with 4 quadrant biopsy every 1 cm)  CR of HGD (assessed through endoscopy with 4 quadrant biopsy every 1 cm)  CR of LGD (assessed through endoscopy with 4 quadrant biopsy every 1 cm)  CR of dysplasia  Reduction in length of BE  Progression to cancer  *Adverse events* | | | *Outcomes:*  CR of BE:  APC Group  - at 4 months: 2/13 patients (15%)  - at 12 months: 0/9 patients (0%)  PDT Group  - at 4 months: 2/13 patients (15%)  - at 12 months: 2/13 patients (15%)  CR of HGD:  APC Group  - at 4 months: 1/1 patient (100%)  - at 12 months: 0/0 patients (0%)  PDT Group  - at 4 months: 2/2 patients (100%)  - at 12 months: 2/2 patients (100%)  CR of LGD:  APC Group  - at 4 months: 7/12 patients (58%)  - at 12 months: 6/9 patients (67%)  PDT Group  - at 4 months: 8/11 patients (73%)  - at 12 months: 8/11 patients (73%)  CR of dysplasia:  APC Group  - at 4 months: 8/13 patients (62%)  - at 12 months: 6/9 patients (67%)  PDT Group  - at 4 months: 10/13 patients (77%)  - at 12 months: 10/13 patients (77%)  (p=0.03)  Reduction in length of BE:  APC Group  - at 4 months: 65% reduction  - at 12 months: 56% reduction  PDT Group  - at 4 months: 57% reduction  - at 12 months: 61% reduction  Progression to cancer:  APC  - at 4 months: 0/13 patients (0%)  - at 12 months: 0/13 patients (0%)  PDT Group  - at 4 months: 0/13 patients (0%)  - at 12 months: 1/13 patients (8%)  *Adverse events:*  APC Group  Strictures: 3/13 patients (23%)  Chest pain, odynophagia and fever: 1/13 patients (8%)  Photosensitivity: 0/13 patients (0%)  Buried glands: 0/13 patients (0%)  PDT Group  Strictures: 2/13 patients (15%)  Chest pain, odynophagia and fever: 0/13 patients (0%)  Photosensitivity: 2/13 patients (15%)  Buried glands: 1/13 patients (8%) | 1 |
| Sharma P, et al. (2006)[18] | | BE  BE + LGD | RCT  Multi-centre  Prospective  APC vs. MPEC  *Countries:* US  *Length of follow-up:* 2 yrs | *Number of patients*: 35  (MPEC Group: 16 patients; APC Group: 19 patients)  *Gender:*  Male: 34  Female: 1  APC Group  *Age*  Mean: 65 yrs  Range: 32 to 84 yrs  MPEC Group  *Age*  Mean: 60 yrs  Range: 42 to 68 yrs  *Prior treatments:* none reported  *Length of Barrett’s:*  APC Group  Mean: 4 cm  Range: 2 to 6 cm  MPEC Group  Mean: 3 cm  Range: 2 to 6 cm  *Inclusion criteria:* none notable  *Exclusion criteria:*  History of esophageal surgery; HGD with EAC; strictures or varices; allergy to PPI; coagulopathy; significant uncontrolled co-morbidities | | APC vs. MPEC  APC Group  *Gas flow:* 1.4 to 1.8 L/minute  *Power:* 60 watts  *Number of sessions:*  Mean: 3.4 sessions/patient  MPEC Group  *Probe*: 10F gold  *Power:* 20 watts  *Number of sessions:* not reported  *Co-interventions:*  Rabeprazole 40mg/day (median) | *Outcomes:*  CR of BE (assessed through endoscopy with 4 quadrant biopsies every 2 cm)  Number of sessions to achieve CR of BE  Progression to cancer  Progression to HGD  *Adverse events* | | | *Outcomes:*  CR of BE at 2 years:  -APC Group: 12/19 patients (63%)  -MPEC Group: 12/16 patients (75%)  Number of sessions to achieve CR of BE:  -APC Group:  Mean: 3.4 sessions / patient  -MPEC Group:  Mean: 3.8 sessions / patient  (p=0.48)  Progression to cancer at 2 years:  -APC Group: 0/19 patients (0%)  -MPEC Group: 0/16 patients (0%)  Progression to HGD at 2 years:  -APC Group: 0/19 patients (0%)  -MPEC Group: 0/16 patients (0%)  *Adverse events:*  APC Group :  Sore throat: 9/19 patients (47%)  Dysphagia: 2/19 patients (11%)  Chest pain: 4/19 patients (21%)  Epigastric pain: 2/19 patients (11%)  Fever, low grade: 1/19 patients (5%)  Stricture: 1/19 patients (5%)  Perforation: 0/19 patients (0%)  Bleeding: 0/19 patients (0%)MPEC Group  Sore throat: 9/16 patients (56%)  Dysphagia: 5/16 patients (31%)  Chest pain: 6/16 patients (38%)  Epigastric pain: 0 /16 patients (0%)  Fever, low grade: 0 /16 patients (0%)  Stricture: 0 /16 patients (0%)  Perforation: 0 /16 patients (0%)  Bleeding: 0 /16 patients (0%) | 1 |
| Thomas T, et al. (2005)[55] *  * Information extracted for BE or HGD patients only | | BE + HGD | Cohort study  Multi-centre  Retrospective  APC vs Esophagectomy vs Non-Intervention vs Surveillance  *Countries:* UK  *Length of follow-up:*  APC and Non-Intervention Groups  Not reported Surveillance Group  Mean: 15 months  Range: 4 to 39 months  Esophagectomy Group  Mean: 21 months  Range: 6 to 36 months | *Number of patients:* 27  (APC: 5 patients; Esophagectomy Group: 8 patients; Non-Intervention Group: 7 patients; Surveillance Group: 7 patients)  APC Group  *Gender:*  Male: 5  *Age:*  Mean: 70 yrs  Range: 54 to 76 yrs  Esophagectomy Group  *Gender:*  Male: 7  Female: 1  *Age:*  Mean: 58 yrs  Range: 46 to 76 yrs  Non-Intervention Group  *Gender:* not reported  *Age:*  Mean: 80 yrs  Range: 74 to 95 yrs  Surveillance Group  *Gender:*  Male: 6  Female: 1  *Age:*  Mean: 65.4 yrs  Range: 55 to 86 yrs  *Prior treatments:* PPI, unspecified  *Length of Barrett’s:*  Mean: 6 cm  Range: 3 to 14 cm  APC Group  Mean: 6 cm  Range: 3 to 9 cm  Surveillance Group  Mean: 5 cm  Range: 2 to 10 cm  *Inclusion criteria:* none notable  *Exclusion criteria:* none notable | | Surveillance vs Esophagectomy vs APC vs Non-Intervention  APC Group  *Gas flow:* not reported  *Power:* not reported  *Treatment time:* not recorded  *Number of sessions:*  Mean: 4 sessions / patient  Range: 1 to 14 sessions / patient  Esophagectomy Group  No details reported  Surveillance Group  *Time between endoscopies:*  Mean: 4.6 months  *Number of treatments:*  Mean 2.9 treatments / patient  Range: 1 to 5 treatments / patient  4 quadrant biopsy every 2 cm in 45% of biopsies  *Co-interventions:*  OM 20-40 mg daily: 17 patients  Lansoprazole 30 mg daily: 14 patients  Pantoprazole 40 mg daily: 1 patient  Rabeprazole 40 mg daily: 2 patients  Ranitidine 150 mg twice daily: 3 patients | *Outcomes:*  Overall survival  Disease specific survival  CR of HGDa  Progression to cancer  *Adverse events:* No BE or HGD specific information available | | | *Outcomes:*  Overall survival:  -APC Group: not reported  -Esophagectomy Group at 21 months (mean): 5/8 patients (62.5%)  -Non-Intervention Group, at unknown follow-up: 2/7 patients (28.6%)  -Surveillance Group: not reported  Disease-specific survival  -APC Group: not reported  -Esophagectomy Group at 21 months (mean): 7/8 patients (88%)*  -Non-Intervention Group, at unknown follow-up: 5/7 patients (71%)**  -Surveillance Group: not reported  CR of dysplasia:  -APC Group at unknown follow-up: 2/5 patients (40%)  -Esophagectomy Group: not reported  -Non-intervention Group: not reported  -Surveillance Group at 15 months (mean): 4/7 patients (57%)  Progression to cancer:  -APC Group at unknown follow-up: 2/5 patients (40%)  -Esophagectomy Group at 21 months (mean): 2/8 patients (25%)  -Non-Intervention Group at unknown follow-up: 2/4 patients (50%)  -Surveillance Group at 15 months: 2/6 patients (33%) | 4 |
| Zoepf T, et al. (2003)[16] | | BE + HGD  BE + LGD | RCT  Single centre  Prospective  APC vs. PDT  *Countries:* Germany  *Length of follow-up*:  APC  Median: 24 months  Range: 4 to 46 months PDT  Median: 27 months Range: 12 to 42 months | *Number of patients:* 20  (APC Group: 10 patients; PDT Group: 10 patients)  *Gender:* not reported  *Age:*  Mean: 68 yrs  Range 44 to 77 yrs  *Prior treatments:* none reported  *Length of Barrett’s:*  PDT  Mean: 3.5 cm  Range: 3 to 12 cm  APC  Mean: 4.0 cm  Range: 3 to 7 cm  *Inclusion criteria:* none notable  *Exclusion criteria:* none notable | | APC vs. PDT  APC  *Power:* 70 watts  *Gas flow:* not reported  *Treatment time:* not reported  *Number of sessions / patient:*  Mean: 4 sessions / patient  Range: 2 to 9 sessions / patient    PDT  *Drug:* 5-ALA  *Dose:* 60 mg/kg  *Route of administration:* oral  *Time to photoactivation:* not reported  *Light source:* diode laser @ non-reported wavelength  *Light dose:* 150J/cm^2^  *Treatment time:* not reported  *Number of sessions / patient:*  Mean: 2 sessions / patient  Range: 1 to 5 sessions / patient  *Co-interventions:* none reported | *Outcomes:*  Reduction in length of BE  *Adverse events* | | | *Outcomes:*  Reduction in length of BE “after treatment”:  APC  Mean 90%  Range: 50 to 100%  PDT  Mean: 90%  Range: 0 to 100%  *Adverse events:*  APC  Nausea / vomiting: 0/10 patients (0%)  Dysphagia, transient: 3/10 patients (30%)  Photosensitivity: 0/10 patients (0%)  Mediastinal emphysema: 1/10 patients (10%)PDT  Nausea / vomiting: 10/10 patients (100%)  Dysphagia, transient 4/10 patients (40%)  Photosensitivity: 0/10 patients (0%)  Mediastinal emphysema: 0/10 patients (0%) | 1 |
| *Non-comparative studies* | | | | | | | | | | | |
| Attwood SE, et al. (2003)[56] | | BE+HGD | Case series  Single centre  Prospective  *Countries:* UK  *Length of follow-up:*  Mean: 37 months  Range: 7 to 78 months | *Number of patients: 29*  *Gender:*  Male: 22  Female: 7  *Age:*  Median: 65 yrs  Range: 43 to 85 yrs  *Prior treatments:* none reported  *Length of Barrett’s:*  Mean: 6 cm  Range: 1 to 12 cm  *Inclusion criteria:*  Unfit for resection (25 patients)  *Exclusion criteria:* none notable | APC under sedation  *Power:* 70 watts  *Gas flow:* 2L/minute  *Treatment time:* not reported  *Number of sessions:*  Median: 2 sessions / patient  Range: 1 to 13 treatments  *Co-interventions:*  PPI, unspecified. | | *Outcomes:*  CR of BE (assessed through endoscopy with 4 quadrant biopsies)  Progression to cancer  Rate of progression to cancer  *Adverse events* | | | *Outcomes:*  CR of BE at 4 to 8 weeks: 22/29 patients (76%)  Progression to cancer at 37 months (mean)*:* 4/29 patients (14%)  Rate of progression to cancer: 3.7 cases/ 1000 patient months  *Adverse events:*  Esophageal perforation 1/29 patients (3%) | 4 |
| Basu KK (2006)[57] | | BE | Case series  Single centre  Prospective  *Countries:* UK  *Length of follow-up:* approximately 4 weeks | *Number of patients:* 33  *Gender:*  Male: 28  Female: 5  *Age:*  Mean: 63.4 yrs  Range: 39 to 79 yrs  *Prior treatments:* none reported  *Length of Barrett’s:*  Mean: 6.5 cm  Range: 4 to 19 cm  *Inclusion criteria:* none notable  *Exclusion criteria:* none notable. | APC  *Power:* 30 watts  *Gas flow*: not reported  *Treatment time:* not reported  *Number of sessions:*  Mean: 4 sessions  Range: 1 to 8 sessions  One third circumference of the esophagus treated / session  *Co-interventions:*  PPI:  -OM 20 mg twice daily (22 patients)  -Lansoprazole 30 mg daily (9 patients)  -Pantoprozole 40 mg daily (2 patients) | | *Outcomes:*  CR of BE  Number of sessions to achieve CR of BE  *Adverse events:* none | | | *Outcomes:*  CR of BE at 4 weeks: 28/33 patients (85%)  Number of sessions to achieve CR of BE:  Mean: 4 sessions  Range: 1 to 8 sessions | 4 |
| Brand B, et al. (2000)[58] | | BE | Case series  *Countries:* Germany  *Length of follow-up:*  Median 12 months  Range: 3 to 25 months | *Number of patients:* 12  *Gender:*  Male: 8  Female: 4  *Age:*  Mean:57 yrs  Range: 42 to 69 yrs  *Prior treatments:* none reported  *Length of Barrett’s:*  Mean: 4 cm  Range: 2 to 11 cm  *Inclusion criteria:* none notable  *Exclusion criteria:* none notable | APC  *Power:* not reported  *Gas flow:* not reported  *Treatment time:* not reported  *Number of treatment sessions:*  Mean: 5 sessions  Range: 4 to 11 sessions  Repeated at intervals of 2 to 3 weeks  *Co-interventions:* none reported | | *Outcomes:*  CR of BE (assessed through 4 quadrant biopsies every 2 cm)  Number of sessions to achieve CR of BE  Recurrence of BE  *Adverse events* | | | *Outcomes:*  CR of BE at one month: 11/12 patients (92%)  Number of sessions to achieve CR of BE:  Mean: 5 sessions  Range: 4 to 11 sessions  Recurrence of BE at 12 months (median): 2/12 patients (16.7%)  *Adverse events:*  Chest pain and odynophagia: 11/12 patients (92%) | 4 |
| Bright T, et al. (2007)[59] | BE  BE + LGD | | RCT  Single centre  Prospective  APC vs Surveillance  *Countries:* Australia  *Length of follow-up:* 1 year | *Number of patients:*40  (APC Group: 20 patients; Surveillance Group: 20 patients)  APC Group  *Gender:*  Male: 15  Female: 5  *Age:*  Median: 47 yrs  Range: 36 to 69 yrs  Surveillance Group  *Gender:*  Male: 17  Female: 3  *Age:*  Mean:51 yrs  Range: 31 to 73 yrs  *Prior treatments:*  Laparoscopic fundoplication for GERD  *Length of Barrett’s:*  Median: 4 cm  Range 2 to 19 cm  *Inclusion criteria:* not reported  *Exclusion criteria:*  HGD or ulcerative esophagitis | APC vs. Surveillance  APC Group  *Gas flow:* 2L/minute  *Power*: 60 watts  *Treatment time:* not reported  *Number of sessions:*  Median: 3 sessions  Range: 2 to 6 sessions  Ablation done in linear lengthwise strips  APC repeated up to 6 times as needed and again one year later  Surveillance Group  Endoscopy one year later  *Co-interventions:* none reported | | *Outcomes:*  CR of BE (assessed through 4 quadrant biopsy)  CR of LGD (assessed through 4 quadrant biopsy)*  Partial response of BE at 1 year  Recurrence of BE given previous response at 1 year  Survival at 1 year  *Adverse events:*  ***follow-up time: 1 year | | *Outcomes:*  CR of BE:  APC Group  -at 4 weeks: 12/20 patients (60%)  -at 1 year: 11/19 patients (58%)  -at 5 years: 8/19 patients (40%)  Surveillance Group  -at 4 weeks: 3/20 patients (15%)  -at 1 year: 2/20 patients (10%)  -at 5 years: 4/20 patients (20%)  CR of LGD*:  -APC Group: 19/19 patients (100%)  -Surveillance Group: 20/20 patients (100%)  Partial response of BE at 1 year:  -APC Group: not reported  -Surveillance Group: 11/20 patients (55%)  Recurrence of BE given previous response at 1 year:  -APC Group: 1/12 patients (8.3%)  Survival at 1 year:  -APC Group: 19/20 patients (95%)  -Surveillance Group: 20/20 patients (100%)  *Adverse events:*  APC Group:  Chest pain and odynophagia observed among “some” patients  Strictures, late after treatment (18 months and 5 years): 2/20 (10%)  Buried glands: 2/20 (10%)  Surveillance Group:  none  *follow-up time: 1 year | | 4 |
| Dumoulin FL, et al.(1997)[60] | BE | | Case report  Single centre  *Countries:* Germany  *Length of follow-up:* not reported | *Number of patients*: 2  *Gender:*  Male: 2  *Age:* 29 and 34 yrs  *Prior treatments:*  OM 20 mg twice daily  Fundoplication  *Length of Barrett’s:*  8cm and 10 cm  *Inclusion criteria:* none notable  *Exclusion criteria:* none notable | APC  *Power:* 50 watts  *Gas flow:* 2L/minute  *Treatment time:* not reported  *Number of sessions:* not reported  *Co-interventions:*  OM 20 mg twice daily  Cisapride 10 mg 3 times daily | | *Outcomes:*  CR of BE  Reduction in size of BE  *Adverse events* | *Outcomes:*  CR of BE post-APC: 0/2  Reduction in the size of BE:  8 cm to 5 cm  10 cm to 4 cm  *Adverse events:*  Chest pain, mild, transient; and dysphagia for one day: 2/2 patients (100%) | | | 4 |
| Familiari L (2003)[61] | | BE (13 patients)  BE + LGD (19 patients) | Case series  Single centre  Prospective  *Countries:* not reported  *Length of follow-up:*  Median: 49.5 months  Range: 24 to 60 months | *Number of patients:* 32  *Gender:*  Male: 26  Female: 6  *Age:*  Median: 58.3 yrs  Range: 29 to 78 yrs  *Prior treatments:* none reported  *Length of Barrett’s:*  BE<3cm: (18 patients)  BE≥3cm: (14 patients)  *Inclusion criteria:* none notable  *Exclusion criteria:* none notable | APC  *Power:* 60 watts  *Gas Flow:* 2L / minute  *Treatment time:* not reported  *Number of sessions:*  Mean: 2.0 sessions / patient  Range: 1 to 3 sessions / patient  *Co-interventions:*  OM 40 mg/day during treatment then 20 mg/ day for 6 months | | *Outcomes:*  CR of BE (assessed through endoscopy with 4 quadrant biopsy every 2 cm)  Number of sessions to achieve CR of BE  *Adverse events* | *Outcomes:*  CR of BE:  -post APC: 32/32 patients (100%)  -6 months: 31/32 patients (97%)  -1 year: 30/32 patients (94%)  -2 years: 29/32 patients (91%)  Number of sessions to achieve CR of BE:  Mean: 2.0 sessions  Range: 1 to 3 sessions  *Adverse events:*  Chest pain, mild: 7/32 patients (22%) | | | 4 |
| Ferraris R, et al. (2007)[62] | | BE | Cohort Study  Multi-centre (5)  Retrospective  *Countries:* Italy  *Length of follow-up:* Mean: 36 months  Range: 18 to 98 months | *Number of patients:* 96  *Gender:*  Male: 70  Female: 26  *Age:*  Mean: 57.1 yrs  Range: 21 to 79 yrs  APC + OM Group  *Number of patients:* 50 patients  *Gender:* not reported  *Age:* not reported  APC + ARS Group  *Number of patients:* 46 patients  *Gender:* not reported  *Age:* not reported  *Prior treatments:* none reported  *Length of Barrett’s:*  Median: 4 cm  Range: 2.5 to 11 cm  *Inclusion criteria:* none notable  *Exclusion criteria:*  Serious disease present | APC  *Power*: 40 watts  *Gas flow:* not reported  *Treatment time:* 10 to 20 minutes  *Number of sessions:*  Mean: 3.2 sessions  Range: 1 to 8 sessions  APC + OM Group  APC as above  OM 40mg/day  APC + ARS Group  APC as above  Laparoscopic fundoplication  *Co-interventions:*  As above | | *Outcomes:*  CR of BE (assessed though 4 quadrant biopsy every 2 cm):  All patients  APC + OM vs. APC + ARS  Number of sessions to achieve CR of BE  *Adverse events:* none | *Outcomes:*  CR of BE:  All patients: 94/96 patients (97.9%)  APC + OM Group vs. APC + ARS:  -1 yr: 97.9% vs. 100%  -2 yr: 94.9% vs. 95.1%  -3 yr: 80.3% vs. 95.1%  -4 yr: 70.5% vs. 87.3%  -5 yr: 43.8% vs. 76.4%  (p<0.05)  Number of sessions to achieve CR of BE:  Mean: 3.2 sessions  Range: 1 to 8 sessions | | | 4 |
| Formentini A (2007)[63] | | BE | Case series  Retrospective  *Countries:* Germany  *Length of follow-up:*  Mean: 17.5 months post ARS  Range: 1 to 54 months | *Number of patients:* 21  *Gender:*  Male: 15  Female: 6  *Age:*  Mean: 45 yrs  Range: 32 to 66 yrs  *Prior treatments:* none reported  *Length of Barrett’s:* <3cm  *Inclusion criteria:* none notable  *Exclusion criteria:* none notable | APC  *Power:* 75 watts  *Gas Flow:* 2L / minute  *Treatment time:* not reported  *Number of sessions:*  Mean: 3.6 sessions / patient  Range: 1 to 12 sessions / patient  *Co-interventions:*  - ARS  360^o^ Nissen fundoplication  Laparoscopic in 17/21 patients or open in 4/21 patients  *Length of stay:*  Mean: 7.9 days  Range: 6 to 13 days  - PPI, unspecified | | *Outcomes:*  CR of BE  *Adverse events* | *Outcomes:*  CR of BE:  - 1 to 1.5 months post APC: 17/17 patients (100%)  - 17.5 months (mean) post ARS: 11/17 patients (65%)  *Adverse events:*  Strictures requiring dilation: 1/21 patients (5%)  Chest pain, transient: 2/21 patients (10%)  Dysphagia and nausea: 1/21 patients (5%) | | | 4 |
| Grade AJ, et al. (1999)[64] | | BE | Clinical trial  Single centre  Prospective  *Countries:* US  *Length of follow-up:*  4 to 6 weeks | *Number of patients:* 9  *Gender:*  Male: 9  Female: 0  *Age:*  Mean: 51.1 yrs  Range: 41 to 61 yrs  *Prior treatments:* none reported  *Length of Barrett’s:*  Mean: 3.6 cm  Range: 2 to 5 cm  *Inclusion criteria:*  None notable  *Exclusion criteria:*  Cardiac disease; lung disease requiring supplemental oxygen; contraindications for endoscopy and extensive biopsy | APC  *Power:* 60 watts  *Gas flow:* 1.6L/minute  *APC application time:*  Mean: 8.0 minutes  Range: 2 to 18 minutes  *Number of sessions:*  Mean: 1.7 sessions / patient  Range: 1 to 3 sessions / patient  Half the circumference of esophagus treated per session  *Co-interventions:*  Lansoprazole 70 mg ± 5 mg/day for the week prior to treatment | | *Outcomes:*  CR of BE (assessed though biopsy every 1 cm)  Partial response of BE  *Adverse events* | *Outcomes:*  CR of BE at 4 to 6 weeks: 7/9 patients (78%)  Partial response of BE: 2/9 patients (22%)  *Adverse events:*  Chest pain, transient, mild: 4/9 patients (44%)  Odynophagia, transient: 1/9 patients (11%) | | | 4 |
| Madisch A, et al. (2005)[65] | | BE | Clinical trial  Prospective  *Countries:* Germany  *Length of follow-up:*  Median: 51 months  Range: 9 to 85 months | *Number of patients:* 73  *Gender:*  Male: 45  Female: 28  *Age:*  Mean: 55 yrs  Range: 28 to 77 yrs  *Prior treatments:* none reported  *Length of Barrett’s:*  Mean: 4 cm  Range: 1 to 12 cm  >2cm: 56/73 patients (76.7%)  *Inclusion criteria:* none notable.  *Exclusion criteria:*  Serious gatrointestinal or extraintestinal disease | APC  *Power:* not reported  *Gas flow:* not reported  *Treatment time:* not reported  *Number of sessions:*  Median: 2 sessions / patient  Range: 1 to 6 sessions / patient  *Co-interventions:*  OM 120mg daily | | *Outcomes:*  CR of BE  Relapse to BE (assessed *histologically* through 4 quadrant biopsies every 2 cm)  Relapse to BE (assessed *endoscopically*)  Number of sessions to achieve CR of BE  *Adverse events* | *Outcomes:*  CR of BE at 3 weeks: 69/70 patients (98.6%)  Relapse to BE at 51 months  (median): 8/66 patients (12.1%)  Annual relapse rate: approximately 3%  Relapse to BE (endoscopy): 13/66 patients (19.7%)  Number of sessions to achieve CR of BE:  Median 2 sessions  Range 1 to 6 sessions  Adverse events:  Bleeding: 0/73  Perforation: 0/73  Strictures: 3/73 | | | 4 |
| Manner H, et al. (2007)[66] *  * Information extracted for BE or HGD patients only | | BE | Case series  Single centre  Prospective  *Countries:* Germany  *Length of follow-up:* not reported | *Number of patients:*104  *Gender:*  not reported  *Age:* not reported  *Prior treatments:*  Previous dysplasia or EAC removed by ER  *Length of Barrett’s:* not reported  *Inclusion criteria:*  Ineligible for or refused surgery  *Exclusion criteria:* none notable | APC  *Power:* 60 watts  *Gas Flow:* 1L / minute  *Treatment time:* not reported  *Number of sessions:*  Mean: 1.1 sessions / patient  Range: 1 to 5 sessions / patient  *Co-interventions:*  PPI, unspecified | | *Outcomes:*  Number of sessions required to achieve CR of BE  *Adverse events* | *Outcomes:*  Number of sessions required to achieve CR of BE:  Mean: 1.1 sessions / patient  Range: 1 to 5 sessions / patient  *Adverse events:*  Pain; cough; dysphagia; arrhythmia; emphysema; gas accumulation in the GI wall; neuromuscular irritation: 10/104 patients (10%)  Strictures: 1/104 patients (1%) | | | 4 |
| Manner H, et al. (2006)[67] *  * Information extracted for BE or HGD patients only | | BE | Case series  Single centre  Retrospective  *Countries:* Germany  *Length of follow-up:* not reported | *Number of patients:* 41  *Gender:* not reported  *Age:* not reported  *Prior treatments:*  Dysplasia or early EAC treated successfully by EMR  *Length of Barrett’s:* not reported  *Inclusion criteria:*  Patients previously received EMR.  *Exclusion criteria:* none notable | APC  *Power:*  Mean: 59 watts  Range: 50 to 60 watts  *Gas Flow:* 2L / minute  *Treatment time:* not reported  *Number of sessions:*  Mean: 1.1 sessions / patient  Range: 1 to 2 sessions / patient  APC done as additive adjunct to ER or ARS  *Co-interventions:* none reported | | *Outcomes:*  Number of sessions to achieve CR of BE  *Adverse events* | *Outcomes:*  Number of sessions to achieve CR of BE:  Mean: 1.1 sessions/ patient ± 0.4 sessions  Range: 1 to 2 sessions / patient  *Adverse events:*  Chest pain: 4/41 patients (10%)  Fever: 4/41 patients (10%)  Strictures: 1/41 patients (2%)  Perforation or bleeding, major: 0/41 patients (0%) | | | 4 |
| Manner H, et al. (2006)[23] | | BE | Case series  Multi-centre  Prospective  *Countries:* Germany  *Length of follow-up:*  Mean: 14 months  Range: 12 to 32 months | *Number of patients:*51  *Gender:*  Male: 41  Female: 10  *Age:*  Mean: 57 yrs  Range: 27 to 77 yrs  *Prior treatments:* none reported  *Length of Barrett’s:*  Mean: 3.6 cm  Range: 1 to 8 cm  *Inclusion criteria:* none notable.  *Exclusion criteria:*  Coagulation disturbances: Quick’s value <50% or platelet count <50/nL  Previous esophageal surgery or endoscopic treatment  Varicies | APC  *Power:* 90 watts  *Gas Flow:* 2L / minute  *Treatment time:* not reported  *Number of sessions:*  Mean: 2.7 sessions / patient  Range: 1 to 8 sessions / patient  *Co-interventions:*  Esomeprazole:  40 mg twice daily during and for 2 weeks post APC, then 40 mg/day until 3 weeks post APC, then 20 mg/day as needed | | *Outcomes:*  CR of BE (assessed through endoscopy with 4 quadrant biopsy every 2 cm)  Partial response of BE (defined as a reduction in BE >50% but <100%)  Number of sessions to achieve CR of BE  *Adverse events* | *Outcomes:*  CR of BE at 14 months (mean): 37/48 patients (77%)  Partial response of BE: 11/48 patients (23%)  Number of sessions to achieve CR of BE:  Mean 2.6 sessions  Range: 1 to 5 sessions  *Adverse events:*  Chest pain: 8/51 patients (16%)  Odynophagia: 2/51 patients (4%)  Fever: 1/51 patients (2%)  Esophageal bleeding requiring transfusion: 2/51 patients (4%)  Strictures: 2/51 patients (4%)  Esophageal perforation: 1/51 patients (2%)  Buried glands: 4/48 (8%) | | | 4 |
| Pedrazzani C, et al. (2005)[68] | | BE+LGD | Clinical trial  Single centre  *Countries*: Italy  *Length of follow-up:*  Mean: 26.3 months  Range: 9 to 45 months | *Number of patients:* 25  *Gender:*  Male: 18  Female: 7  *Age:*  Mean: 61.7 yrs  Range: 34 to 74 yrs  *Prior treatments:* none reported  *Length of Barrett’s:*  Mean: 3.4 cm  Range: 1 to 13 cm  *Inclusion criteria:* none notable  *Exclusion criteria:*  Previous malignancies or intercurrent diseases affecting prognosis | APC  *Power:*90 watts  *Gas flow*: 2L/minute  *Treatment time:* not reported  *Number of sessions:*  Mean: 1.6 sessions  Range: 1 to 4 sessions  *Co-interventions:*  Pantoprazole or esomeprazole 40 mg twice daily one week before and throughout treatment | | *Outcomes:*  CR of BE (assessed through 4 quadrant biopsy)  Number of APC sessions to CR of BE  *Adverse events* | *Outcomes:*  CR of BE  -at 1 month: 24/25 patients (96%)  -at 26.3 months (mean): 23/25 patients (92%)  Number of APC sessions to CR of BE:  1 treatment: 15/25 patients (60%)  2 treatments: 6/25 patients (24%)  ≥3 treatments: 4/25 patients (16%)  *Adverse events:*  Chest pain: 11/40 sessions (23%)  Fever: 7/40 sessions (18%)  Dysphagia: 2/40 sessions (5%)  Ulcer formation: 2/40 sessions (5%)  Bleeding, severe: 1/40 sessions (5%) | | | 4 |
| Pereira-Lima JC, et al. (2000)[69] | | BE (18 patient)  BE + LGD (14 patients)  BE + HGD (1 patient) | Case series  Single centre  *Countries:* Brazil  *Length of follow-up:*  Mean: 10.6 months  Range: 6 to 18 months | *Number of patients:* 33  *Gender:*  Male: 21  Female: 12  *Age:*  Mean: 55.2 yrs  Range: 21 to 84 yrs  *Prior treatments:*  ARS (9 patients)  PPI, unspecified (24 patients)  *Length of Barrett’s:*  Mean: 4.05 cm  Range: 0.5 to 7 cm  *Inclusion criteria:* none notable  *Exclusion criteria:* none notable | APC  *Power:* 65 to 70 watts  *Gas Flow:* 2L / minute  *Treatment time:* not reported  *Number of sessions:*  Mean: 1.96 sessions / patient  Range: 1 to 4 sessions / patient  Maximum of 4 cm length circumferentially ablated / session  *Co-interventions:*  OM 60 mg/day until BE ablation; then OM 30 mg/day or ARS recommended | | *Outcomes:*  CR of BE (assessed through endoscopy with 6 biopsies every 1 cm)  Recurrence of BE at a mean of 10.6 months  *Adverse events* | *Outcomes:*  CR of BE at 1 to 2 months: 32/33 patients (97%)  Recurrence of BE at 10.6 months (mean): 1/33 patients (3%)  *Adverse events:*  Chest pain, moderate to severe; and odynophagia: 18/33 patients (55%)  Pleural effusion and high fever (39^o^C): 5/33 patients (15%)  Strictures: 3/33 patients (9%)  Chest pain; pneumomediastinum; subcutaneous emphysema 1 hour post APC: 1/33 patients (3%)  Buried glands: 0/33 patients (0%) | | | 4 |
| Pinotti AC, et al. (2004)[70] | | BE | Case series  Single centre  Prospective  *Countries:* Brazil  *Length of follow-up:*  Mean: 17 months  Range: 6 to 27 months | *Number of patients:* 19  *Gender:*  Male: 11  Female: 8  *Age:*  Mean: 52.5 yrs  Range: 32 to 72 yrs  *Prior treatments:* none reported  *Length of Barrett’s:*  Mean: 3.55 cm  Range: 1 to 9 cm  *Inclusion criteria:* none notable  *Exclusion criteria:* none notable | APC +ARS  *Power:* 50 watts  *Gas Flow:* 2L / minute  *Treatment time:*  *Number of sessions:*  Mean: 2 sessions / patient  Range: 1 to 6 sessions / patient  Half circumference treated in patients with long BE segments  *Co-interventions:*  ARS (Laparoscopic Nissen fundoplication) preceded APC in all patients | | *Outcomes:*  CR of BE  Recurrence of BE  *Adverse events* | *Outcomes:*  CR of BE at 2 months: 18/19 patients (95%)  Recurrence of BE at 17 months (mean):  1/19 patients (5%)  *Adverse events:*  Strictures or perforation: 0/19 patients (0%)  Dysphagia, transient; and odynophagia: 4/19 patients (21%)  Chest pain, transient: 17/19 patients (89%)  - duration 3 days: 11/19 patients (58%)  - duration 7 days: 4/19 patients (21%)  - duration >7 days: 2/19 patients (11%) | | | 4 |
| Tigges H, et al. (2001)[71] | | BE | Case series  Single centre  *Countries:* Germany  *Length of follow-up:* 1 yr | *Number of patients:* 30  *Gender:*  Male: 23  Female: 7  *Age:*  Mean: 53.5 yrs  Range: 31 to 77 yrs  *Prior treatments:*  PPI, unspecified, >6 months  *Length of Barrett’s:*  Median: 3 cm  Range: 1 to 10 cm  *Inclusion criteria:* none notable  *Exclusion criteria:*  Severe co-morbidity  Life expectancy <5 yrs  History of upper GI surgery including ARS | APC + ARS  *Power:* up to 150 watts  *Gas Flow:* 0.1 to 0.9L / minute  *Treatment time:*  Median: 35 minutes  Range: 15 to 50 minutes  *Number of sessions:* not reported  Half circumference treated at first session  Precedent to ARS  *Co-interventions:*  OM 40 / day  ARS: laparoscopic Nissen fundiplication or 240^o^ Toupet fundiplication (26/30 patients) | | *Outcomes:*  CR of BE (assessed through endoscopy with 4 quadrant biopsy every 1 cm)  Progression to cancer  *Adverse events* | *Outcomes:*  CR of BE:  - 1.5 to 2 months (post-APC): 22/22 patients (100%)  - 1 yr (post-ARS): 20/22 patients (91%)  Progression to cancer at 1 year: 0/22 patients (0%)  *Adverse events:*  Post APC  Dysphasia, transient or odynophagia: 2/30 patients (7%)  Strictures: 1/30 patients (3%)  Persistent dysphagia, perforation or bleeding: 0/30 patients (0%)  Post-ARS  Pneumothroax: 2/22 patients (9%)  Skin emphysema secondary to pneumoperitoneum: 1/22 patients (4.5%) | | | 4 |
| Van Laethem JL, et al. (2001)[72] *  * Information extracted for BE or HGD patients only | | BE + HGD | Case series  Single centre  Prospective  *Countries:* Belgium  *Length of follow-up:*  Mean: 25.5 months  Range: 12 to 36 months | *Number of patients:* 7  *Gender:*  Male: 5  Female: 2  *Age:*  Mean: 72.6 yrs  Range: 64 to 85 yrs  *Prior treatments:* none reported  *Length of Barrett’s:*  Mean: 4.6 cm  Range: 3 to 7 cm  *Inclusion criteria:*  Ineligible for or refused surgery  *Exclusion criteria:* none notable | APC  *Power:* 90 watts  *Gas Flow:* not reported  *Treatment time:* not reported  *Number of sessions:*  Mean: 2.83 sessions / patient  Range: 1 to 6 sessions / patient  *Co-interventions:*  OM 40 mg/day | | *Outcomes:*  Survival  CR of BE (assessed through endoscopy with 4 quadrant biopsy every 1 to 2 cm)  CR of HGD  Non-response of dysplasia  Progression to cancer  *Adverse events:* No BE or HGD specific information available. | *Outcomes:*  Survival at 25.5 months (mean): 6/7 patients (86%)  CR of BE  -at 1 month: 4/7 patients (57 %)  -at a mean of 25.5 months: 4/7 patients (57 %)  CR of HGD  -at 1 month: 6/7 patients (85.7%)  -at 25.5 months (mean): 5/7 patients (74%)  Non-response of BE/dysplasia  -at 1 month: 1/7 patients (14%)  -at 25.5 months (mean): 1/7 patients (14%)  Progression to cancer at 25.5 months (mean): 1/7 patients (14%)  *Adverse events:* | | | 4 |
| Van Laethem JL, et al. (1998)[73] | | BE  BE + LGD | Case series  Single centre  *Countries:* Belgium  *Length of follow-up:* 12 months | *Number of patients:* 31  *Gender:*  Male: 25  Female: 6  *Age:*  Mean: 64 yrs  Range: 46 to 76 yrs  *Prior treatments:*  OM 20mg/day  *Length of Barrett’s:*  Mean: 4.5 cm  Range: 3 to 11 cm  *Inclusion criteria:* none notable  *Exclusion criteria:*  Intolerant to upper GI endoscopy; life expectancy <5yrs; inter-current disease affecting prognosis; previous history of gastro-esophageal surgery; use of NSAIDs | APC  *Power:* not reported  *Gas Flow:* not reported  *Treatment time:* not reported  *Number of sessions:*  Mean: 2.4 sessions / patient  Range: 1 to 4 sessions / patient  *Co-interventions:*  OM 40 mg/day or 10mg/day (randomly assigned) for < 3 months | | *Outcomes:*  CR of BE (assessed through endoscopy with 4 quadrant biopsy every 2 cm)  *Adverse events* | *Outcomes:*  CR of BE:  -1 month: 19/31 patients (61%)  -3 months: 15/31 patients (48%)  -12 months: 9/17 patients (53%)  *Adverse events:*  Buried glands: 6/31 patients (19%)  Odynophagia, transient or dysphagia, transient: most patients  Chest pain, persistent, odynophagia persistent, dysphagia, persistent: 2/31 patients (6%)  Strictures: 2/31 patients (6%)  Esophageal bleeding requiring transfusion: 1/31 patients (3%)  Reflux esophagitis (at 3 months): 8/31 patients (25%) | | | 4 |

***Note:*** ALA (aminolevulinic acid), APC (argon plasma coagulation), ARS (anti-reflux surgery), BE (Barrett’s esophagus), CR (complete response), EAC (esophageal adenocarcinoma), EMR (endoscopic mucosal resection), GERD (gastroesophageal reflux disease), GI (gastrointestinal), HGD (high grade dysplasia), IV (intravenous), LGD (low grade dysplasia), MPEC (multipolar electrocoagulation), OM (omeprazole), PDT (photodynamic therapy), PPI (proton pump inhibitor), RCT (randomized controlled trial)
