# Supplementary material for: Endoscopic treatments for Barrett's esophagus: a systematic review of safety and effectiveness compared to esophagectomy
Source: BMC Gastroenterol. 2010 Sep 27;10:111. doi: 10.1186/1471-230X-10-111 (PMC2955687; doi:10.1186/1471-230X-10-111)
Supplement: Additional file 3 — Studies of cryoablation, combined endoscopic mucosal resection (EMR) and photodynamic therapy (PDT), and thermocoagulation for Barrett's esophagus with/without dysplasia. Details of study and patient characteristics, outcomes and study quality of the included studies of cryoablation, combined EMR and PDT, and thermocoagulation for BE with/without dysplasia are presented in Additional file 3. [file 1471-230X-10-111-S3.DOC]

| **Study authors (year published)**  Additional file 3. Studies of cryoablation, combined EMR and PDT, and thermocoagulation for Barrett's esophagus with/without dysplasia | **Cancer / Cell Type** | **Study Design** | **Patients** | **Intervention** | **Outcome Measures** | **Findings** | **Study quality** |
| --- | --- | --- | --- | --- | --- | --- | --- |
| Cryoablation | | | | | | | |
| *Comparative studies* | | | | | | | |
| None | | | | | | | |
| *Non-comparative studies* | | | | | | | |
| Dumot JA, et al. (2008)[74]*  * Information extracted for BE or HGD patients only | BE+HGD (20 patients) | Clinical trial  Single centre  *Countries:* US  *Length of follow-up:* Not reported | *Number of patients*: 20  *Gender*:  Not reported  *Age*:  Not reported  *Prior treatments:* none reported  *Length of Barrett’s:* not reported  *Inclusion criteria:* none notable  *Exclusion criteria:* none notable | Cryoablation  *Device:* not reported  *Drug:* liquid nitrogen spray  *Method:* low pressure spray  *Treatment time:* not reported  *Number of sessions:*  Mean: 4 sessions  IQR: 2 to 6 sessions  *Co-interventions:* none reported | *Outcomes:*  CR of HGD  *Adverse events:* No BE or HGD specific information available. | *Outcomes:*  CR of HGD at unreported follow-up: 16/18 patients (89%) | 4 |
| Johnston MH (2005)[75] | BE (3 patients)  BE + LGD (5 patients)  BE + HGD (1 patient)  BE + indefinite for dysplasia (2 patients) | Clinical trial  Single centre  Prospective  *Countries:* US  *Length of follow-up:*  Mean: 12 months  Range: 6 to 20 months | *Number of patients*: 11  *Gender*:  Male:11  *Age*:  Mean: 59 yrs  Range: 50 to 74 yrs  *Prior treatments:*  PPI, unspecified.  *Length of Barrett’s:*  Mean: 4.6cm  Range: 1 to 8 cm  *Inclusion criteria:*  Patients with BE in an established registry  Multiple previous endoscopies  Use of PPI  *Exclusion criteria:* none notable | Cryoablation  *Device:* 9F cryogenic catheter  *Drug:* liquid nitrogen spray  *Method:* low pressure spray hemi-circumferentially to 4 cm long segments/ session  *Treatment time:* Not reported  *Number of sessions:*  Mean: 4.8 sessions  Range: 1 to 8 sessions  Tissue frozen for 20 seconds, permitted to thaw, then re-frozen for 20 seconds  *Co-interventions:*  Rabeprazole 40 mg 3 times a day during treatment period | *Outcomes:*  CR of BE (assessed through endoscopy with 4 quadrant biopsies every 2 cm)  CR of HGD  Number of sessions to achieve CR of BE  *Adverse events* | *Outcomes*:  CR of BE  - at 1 month: 9/11 patients (81.8%)  - at a mean of 12 months: 7/11 patients (64%)  CR of HGD at 1 month: 1/1 patients (100%)  Number of sessions to achieve CR of BE:  Mean 3.6 sessions  Adverse events:  Chest pain: 2/11 (22.2%)  Dysphagia: 1/11 (11.1%)  Bleeding: 0/11 (0%)  Perforation: 0/11(0%) | 4 |
| Combined EMR+PDT | | | | | | | |
| *Comparative studies* | | | | | | | |
| Behrens A, et al. (2005)[25] | BE + HGD | Cohort study  Single centre  Prospective  PDT + EMR vs. PDT vs. EMR  *Countries:* Germany  *Length of follow-up:*  Mean: 38 months  Range: 7 to 61 months | *Number of patients:* 44  (PDT+EMR: 3 patients; PDT Group: 27 patients; EMR Group: 14 patients)  *Gender:*  Male: 38  Female: 6  *Age:*  Mean:61 yrs  Range: 33 to 79 yrs  PDT + EMR Group  *Number of patients:* 3 patients  *Gender:* not reported  *Age:* not reported  EMR Group  *Number of patients:* 14 patients  *Gender:* not reported  *Age:* not reported  PDT Group  *Number of patients:* 27 patients  *Gender:* not reported  *Age:* not reported  *Prior treatments:* none reported  *Length of Barrett’s:* not reported  *Inclusion criteria:* none notable  *Exclusion criteria:* none notable | PDT + EMR vs. EMR vs. PDT  PDT Group  Patients with microscopic / histologic HGD  *Drug:* 5-ALA  *Dose:* 60 mg/kg  *Route of administration:* oral  *Light source*: dye laser @ 630 to 635nm  *Light dose:* not reported  *Time to photoactivation:* 4 to 6 hours  *Treatment time:* not reported  *Number of sessions:*  Mean: 1 session/patient  Range: 1 to 4 sessions / patient  EMR  *Technique:* EMR with ligation, or cap and snare  *Injection:* none  *Number of treatments:* not reported  PDT + EMR Group  Details as above.  *Co-interventions:*  OM 40 mg IV twice daily or Pantoprazole 40 mg IV twice daily | *Outcomes:*  CR of dysplasia  Recurrence of HGD  Progression to cancer  *Adverse events:* | *Outcomes:*  CR of dysplasia  - at 1 month (after 1 treatment session):  -All patients: 39/43 patients (91%)  -PDT + EMR Group: 2/3 patients (67%)  -EMR Group: 13/14 patients (93%)  -PDT Group: 26/27 patients (96%)  … at 38 months (mean) (after 1 to 4 sessions)  -All patients: 29/35 patients (83%)  Recurrence of HGD at 38 months (mean): 4/35 patients (11%)  Progression to cancer at 38 months (mean): 2/35 patients (6%)  *Adverse events:*  PDT Group  Vomiting, severe: 1/27 patients (4%)  Nausea: 14/27 patients (52%)  EMR Group  Bleeding, minor: 4/17 patients (24%) | 4 |
| *Non-comparative studies* | | | | | | | |
| Wolfsen HC, et al. (2004)[76] | BE + HGD | Case series  Single centre  *Countries:* US  *Length of follow-up:*  Median: 13 months  Range: 6 to 46 months | *Number of patients:*3  *Gender:*  Male: 3  *Age:*  Mean: 68.67 yrs  Range: 68 to 69 yrs  *Prior treatments:* none reported  *Length of Barrett’s:*  Mean: 3.67 cm  Range: 3 to 4 cm  *Inclusion criteria:*  Ineligible for or refused surgery  *Exclusion criteria:* none notable | PDT + EMR  PDT  *Drug:* porfimer sodium  *Dose:* 2 mg/kg  *Route of administration:* IV  *Light source*: diode laser @ 630nm  *Light dose:* 175 to 250 J/cm^2^  *Time to photoactivation:* 48 hours  *Treatment time:* not reported  *Number of sessions:* 1 session / patient (assumed)  Provided 4 to 6 weeks post EMR  EMR  *Technique:* inject and cut  *Devices:* not reported  *Circumferential vs. focal:* focal  *Injection:* yes  *Solution:* saline ± epinephrine (1:10,000)  *Number of treatments:* not reported  Provided for focal lesions / mucosal irregularities before PDT  *Co-interventions*:  PPI, unspecified | *Outcomes:*  Survival  CR of BE  CR of dysplasia  *Adverse event* | *Outcomes:*  Survival at 13 months (median): 3/3 patients (100%)  CR of BE at 13 months (median): 3/3 patients (100%)  CR of dysplasia at 13 months (median): 3/3 patients (100%)  *Adverse events:*  Strictures: 0/3 patients (0%)  Chest pain, mild: common | 4 |
| Thermocoagulation | | | | | | | |
| *Comparative studies* | | | | | | | |
| None | | | | | | | |
| *Non-comparative studies* | | | | | | | |
| Michopoulos S, et al. (1999)[77] | BE | Case series  *Countries:* Greece  *Length of follow-up:*  Median: 14.5 months  Range: 6 to 35 months | *Number of patients:* 13  *Gender:*  Male: 8  Famle: 5  *Age:*  Mean: 54.6 yrs  Range: 32 to 71 yrs  *Prior treatments:* no previous antireflux or gastric surgery  *Length of Barrett’s:*  Range: 2 to 6 cm  *Inclusion criteria:*  Not reported.  *Exclusion criteria:* HGD, EAC, esophageal ulcer or stricture, pregnancy or lactation, known allergy to OM or poor general medical condition and specialized columnar epithelium identified on biopsies taken from an endoscopically normal gastroesophageal junction. | Thermocoagulation  *Technique:* Heat probe ablation  *Devices:* 2.4 mm Olympus heat probe  *Number of treatment sessions:* mean (SD): 2.77 (1.69)  Range: 1 to 5  *Co-interventions*:  OM 20 mg, sphincter relaxing drugs, nonsteroidal anti-inflammatory drugs, etidroonate. | *Outcomes:*  CR of BE (assessed through endoscopy)  *Adverse event* | *Outcomes:*  1 patient lost in the follow-up  CR of BE at postablation: 100% (13/13) patients  CR of BE at 6 months: 91.7% (11/12) patients  *Adverse events:*  No symptoms or only mild retrosternal discomfort at 24 hours after the ablation: 92.3% (12/13) patients  Acute retrosternal pain: 7.7% (1/13) (Endoscopy revealed an esophageal ulcer in the following day) | 4 |

***Note:*** ALA (aminolevulinic acid), BE (Barrett’s esophagus), CR (complete response), EAC (esophageal adenocarcinoma), EMR (endoscopic mucosal resection), HGD (high grade dysplasia), IV (intravenous), LGD (low grade dysplasia), OM (omeprazole), PDT (photodynamic therapy), PPI (proton pump inhibitor)
