# Supplementary material for: Endoscopic treatments for Barrett's esophagus: a systematic review of safety and effectiveness compared to esophagectomy
Source: BMC Gastroenterol. 2010 Sep 27;10:111. doi: 10.1186/1471-230X-10-111 (PMC2955687; doi:10.1186/1471-230X-10-111)
Supplement: Additional file 4 — Studies of endoscopic mucosal resection (EMR) for Barrett's esophagus with/without dysplasia. Details of study and patient characteristics, outcomes and study quality of the included studies of EMR for BE with/without dysplasia are presented in Additional file 4. [file 1471-230X-10-111-S4.DOC]

| **Study authors (year published)**  Additional file 4. Studies of endoscopic mucosal resection (EMR) for Barrett's esophagus with/without dysplasia | **Cancer / Cell Type** | **Study Design** | **Patients** | **Intervention** | **Outcome Measures** | **Findings** | **Study quality** |
| --- | --- | --- | --- | --- | --- | --- | --- |
| *Comparative studies* | | | | | | | |
| Behrens A, et al. (2005)[25] | BE + HGD | Cohort study  Single centre  Prospective  EMR vs. PDT vs. PDT + EMR  *Countries:* Germany  *Length of follow-up:*  Mean: 38 months  Range: 7 to 61 months | *Number of patients:* 44  (EMR Group: 14 patients; PDT Group: 27 patients; PDT+EMR: 3 patients)  *Gender:*  Male: 38  Female: 6  *Age:*  Mean:61 yrs  Range: 33 to 79 yrs  EMR Group  *Number of patients:* 14 patients  *Gender:* not reported  *Age:* not reported  PDT Group  *Number of patients:* 27 patients  *Gender:* not reported  *Age:* not reported  PDT + EMR Group  *Number of patients:* 3 patients  *Gender:* not reported  *Age:* not reported  *Prior treatments:* none reported  *Length of Barrett’s:* not reported  *Inclusion criteria:* none notable  *Exclusion criteria:* none notable | PDT vs. EMR vs. PDT + EMR  EMR  *Technique:* EMR with ligation, or cap and snare  *Injection:* none  *Number of treatments:* not reported  PDT Group  Patients with microscopic / histologic HGD  *Drug:* 5-ALA  *Dose:* 60 mg/kg  *Route of administration:* oral  *Light source*: dye laser @ 630 to 635nm  *Light dose:* not reported  *Time to photoactivation:* 4 to 6 hours  *Treatment time:* not reported  *Number of sessions:*  Mean: 1 session/patient  Range: 1 to 4 sessions / patient  PDT + EMR Group  Details as above.  *Co-interventions:*  OM 40 mg IV twice daily or Pantoprazole 40 mg IV twice daily | *Outcomes:*  CR of HGD  Recurrence of HGD  Progression to cancer  *Adverse events:* | *Outcomes:*  CR of dysplasia …  … at 1 month (after 1 treatment session):  -All patients: 39/43 patients (91%)  -EMR Group: 13/14 patients (93%)  -PDT Group: 26/27 patients (96%)  -PDT + EMR Group: 2/3 patients (67%)  … at 38 months (mean) (after 1 to 4 sessions)  -All patients: 29/35 patients (83%)  Recurrence of HGD at 38 months (mean): 4/35 patients (11%)  Progression to cancer at 38 months (mean): 2/35 patients (6%)  *Adverse events:*  PDT Group  Vomiting, severe: 1/27 patients (4%)  Nausea: 14/27 patients (52%)  EMR Group  None reported | 4 |
| Reed MF, et al. (2005)[20] | BE + HGD | Cohort study  Single centre  Retrospective  Esophagectomy vs Endoscopic Therapy vs Observation  *Countries:* not reported  *Length of follow-up*: 10 yrs | *Number of patients: 115*  (Endoscopic Therapy Group: 47 patients – 5 EMR, 42 PDT; Esophagectomy Group: 49 patients; Observations Group: 19 patients)  *Age:*  Mean 65 yrs  Range 30 to 87 yrs  Gender:  Male: 95  Female: 20  Endoscopic Group  *PDT: 42 patients*  *EMR 5 patients*  *Age:*  Mean 70 yrs  Range 30 to 89 yrs  *Gender:*  Male: 38  Female: 9  Esophagectomy Group  *Age:*  Mean 59 yrs  Range 32 to 79 yrs  *Gender:*  Male: 40  Female: 9  Observation Group:  *Age:* not reported  *Gender:* not reported  *Prior treatments:* none reported  *Length of Barrett’s:* not reported  *Inclusion criteria*: none notable  *Exclusion criteria*: none notable | Endoscopic Group  EMR or PDT  No details reported  Esophagectomy Group:  Surgical resection done within 60 days of diagnosis  *Type of surgery:*  -TTE: 20 patients (41%)  -Ivor Lewis: 18 patients (37%)  -THE: 7 patients (17%)  -various or mixed techniques: 4 patients(8%)  Observation Group:  No details reported  *Co-interventions:* none reported | *Outcomes:*  Disease specific survival  Overall survival  CR of HGD  Progression to cancer  *Adverse event:* | *Outcomes:*  Disease specific survival at 5 years:  -Endoscopic Group: not reported  -Esophagectomy Group: 94%  -Observation Group: not reported  Overall survival:  Endoscopic Group: not reported  Esophagectomy Group  - at 5 yrs: 83%  - at 10 yrs: 64%  Observation Group: not reported  CR of HGD, follow-up unknown:  Endoscopic Group  PDT: 37/42 patients (88%)  EMR 3/5 patients (60%)  Esophagectomy Group  not reported  Observation Group  0/13 patients (0%)  Progression to cancer  -Endoscopic Group: 6/47 patients  -Esophagectomy Group: not reported  -Observation 7/13 patients  *Adverse events:*  Esophagectomy Group  Post op anastomotic leak: 2/49 patients (4%)  Death secondary to large cerebrovascular accident post-op: 1/49 patients (2%) | 4 |
| *Non-comparative studies* | | | | | | | |
| Giovannini M, et al. (2004)[78] | BE + HGD | Case series  Single centre  *Countries:* France  *Length of follow-up*  Mean: 18 months  Range: 6 to 34 months | *Number of patients: 12*  *Gender:*  Male: 5  Female: 7  *Age:*  *M*ean: 61 yrs  Range 42 to 71 yrs  *Prior treatments:* none reported  *Length of Barrett’s:* not reported  *Inclusion criteria:* none notable  *Exclusion criteria:* none notable | EMR  *Technique:* inject and cut  *Injection:* yes (not reported)  *Number of treatments:*  Median: 2 sessions/patient  Hemicircumferential excision every session  *Co-interventions*:  PPI (details not reported) | *Outcomes:*  CR of HGD  Recurrence of HGD  *Adverse events:* | *Outcomes:*  CR of HGD  - at 1 month: 12/12 patients (100%)  - at 12 months: 10/12 patients (83%)  - at 18 months: 10/12 patients (83%)  Recurrence of HGD at 12 months: 2/12 (18%)  *Adverse events:*  Bleeding, managed endoscopically: 3/12 patients (25%)  Perforation: 0/12 patients (0%)  Stricture: 0/12 patients (0%) | 4 |
| Mino-Kenudson M, et al. (2005)[79]*  * Information extracted for BE or HGD patients only | BE + HGD | Case series  Single centre  *Countries:* US  *Length of follow-up*  Mean: 23.3 months  Range: 7 to 41 months | *Number of patients:*3  *Gender:* not reported  *Age:* not reported  *Prior treatments:* none reported  *Length of Barrett’s:*  Mean: 6.7 cm  Range: 2 to 15 cm  *Inclusion criteria:*  Ineligible for or refused surgery  *Exclusion criteria:* none notable | EMR  *Technique:* inject and cut  *Injection:* yes (epinephrine in saline 1:100,000)  *Number of treatments:* not reported  Circumferential  *Co-interventions*:  PPI (details not reported) (1 patient) | *Outcomes:*  CR of BE  *Adverse events:* none | *Outcomes:*  CR of BE at 23.3 months (mean): 2/3 patients: (67%) | 4 |
| Seewald S, et al. (2003)[80]  * Information extracted for BE or HGD patients only | BE + HGD | Case series  Single centre  *Countries:* Germany  *Length of follow-up:*  Mean: 14 months  Range: 5 to 24 months | *Number of patients: 3*  *Gender:*  Male: 2  Female: 1  *Age:*  *M*ean: 53.3 yrs  Range 43 to 59 yrs  *Prior treatments:* none reported  *Length of Barrett’s:*  Mean: 2 cm  Range: 2 to 2 cm  *Inclusion criteria:* none notable  *Exclusion criteria:* none notable | EMR  *Technique:* simple snare resection  *Injection:* none  *Number of treatments:*  Mean: 1.66 sessions/patient  Range:1 to 3 cm  Circumferential  *Co-interventions:*  PPI, unspecified | *Outcomes:*  CR of BE (assessed through endoscopy with biopsy)  CR of HGD  Progression to cancer  *Adverse events:* No BE or HGD specific information available. | *Outcomes:*  CR of BE at 14 months (mean): 0/3 patients (0%)  CR of HGD at 14 months (mean): 1/3 patients (33%)  Progression to cancer at 14 months (mean): 0/3 patients (0%) | 4 |
| Tang SJ, et al. (2008)[81] | BE + LGD + HGD | Case report  Single centre  *Countries:* US  *Length of follow-up:* 3 months | *Number of patients:* 1  *Gender:* Male  *Age:*  58  *Prior treatments:*  PPI, unspecified  *Length of Barrett’s:* 14 cm  *Inclusion criteria*:  Refused surgery  *Exclusion criteria*: none notable | EMR  *Technique:* EMR with ligation  *Injection:* none  *Number of treatments:* 2 sessions  Circumferential  *Co-interventions:*  PPI (drug and dose not reported) | *Outcomes:*  CR of BE (assessed through endoscopy and biopsy)  CR of HGD  *Adverse events*: | *Outcomes:*  CR of BE at 3 months: 1/1 patients (100%)  CR of dysplasia at 3 months: 1/1 patients (100%)  *Adverse events*:  Pneumonia: 1/1 patient (100%)  DVT secondary to IV line: 1/1 patient (100%)  Chest and epigastric pain, mild, duration ≤ 7days: 1/1 patient (100%) | 4 |

***Note:*** ALA (aminolevulinic acid), BE (Barrett’s esophagus), CR (complete response), DVT (deep vein thrombosis), EMR (endoscopic mucosal resection), HGD (high grade dysplasia), IV (intravenous), LGD (low grade dysplasia), OM (omeprazole), PDT (photodynamic therapy), PPI (proton pump inhibitor)
