# Supplementary material for: Endoscopic treatments for Barrett's esophagus: a systematic review of safety and effectiveness compared to esophagectomy
Source: BMC Gastroenterol. 2010 Sep 27;10:111. doi: 10.1186/1471-230X-10-111 (PMC2955687; doi:10.1186/1471-230X-10-111)
Supplement: Additional file 5 — Studies of laser ablation for Barrett's esophagus with/without dysplasia. Details of study and patient characteristics, outcomes and study quality of the included studies of laser ablation for BE with/without dysplasia are presented in Additional file 5. [file 1471-230X-10-111-S5.DOC]

| **Study authors (year published)**  Additional file 5. Studies of laser ablation for Barrett's esophagus with/without dysplasia | **Cancer / Cell Type** | **Study Design** | **Patients** | **Intervention** | **Outcome Measures** | **Findings** | **Study quality** |
| --- | --- | --- | --- | --- | --- | --- | --- |
| *Comparative studies* | | | | | | | |
| None | | | | | | | |
| *Non-comparative studies* | | | | | | | |
| Barham CP, et al. (1997)[82] | BE | Case series  Single centre  Countries: UK  Laser ablation vs. OM  *Length of follow-up:* 6 weeks | *Number of patients:* 16  *Gender:*  Male: 12  Female: 4  *Age:*  Mean: 58.7 yrs  Range: 36 to 76 yrs  *Prior treatments:*  OM, unspecified  *Length of Barrett’s:* not reported  *Inclusion criteria:* none notable  *Exclusion criteria:* none notable | Laser ablation  *Type:*  KTP @512 nm  *Power*: 20 watts  *Dose:* not reported  *Treatment time:* not reported  *Number of sessions:*  Mean: 3.4 sessions / patient  Range: 1 to 6 sessions  Approximately 30% circumference ablated / session  *Co-interventions:*  OM 40 mg / day | *Outcomes:*  CR of BE  Number of sessions to achieve CR of BE  *Adverse events* | *Outcomes:*  CR of BE at 6 weeks: 13/16 patients (81%)  Number of sessions to achieve CR of BE:  Mean: 3 sessions  Range: 1 to 6 sessions  *Adverse events:*  Chest pain, mild, duration <48 hours: common  Buried glands: 11/16 patients (69%) | 4 |
| Bonavina L, et al. (1999)[83] | BE | Case series  Single centre  Prospective  *Countries:* Italy  *Length of follow-up:*  Mean: 14 months  Range: 4 to 32 months | *Number of patients:* 18  *Gender:*  Male: 14  Female: 4  *Age:*  Mean: 55 yrs  Range: 32 to 70 yrs  *Prior treatments:* none reported  *Length of Barrett’s:*  Mean: 4.3 cm  *Inclusion criteria:* none notable  *Exclusion criteria:*  >70 yrs | Laser ablation  *Type:* Nd:YAG @ 1064nm  *Power:*60 watts  *Dose:*  Mean: 2800 J  Range: 600 to 4800 J  *Treatment time:* not reported  *Number of sessions:*  Mean: 3 sessions / patient  Range: 1 to 5 sessions  Half circumference treated / session  *Co-interventions:*  OM 40 mg daily  ARS (12 patients) | *Outcomes:*  CR of BE (assessed through endoscopy and biopsy)  Partial response of BE  No response to BE  Progression to cancer  *Adverse events* | *Outcomes:*  CR of BE  - at 4 weeks: 11/18 patients (61%)  - at 14 months (mean): 9/18 patients (50%)  Partial response of BE at 4 weeks: 5/18 patients (28%)  No response to BE at 4 weeks: 2/18 patients (11%)  Progression to cancer at 14 months (mean): 1/18 patients (5.6%)  *Adverse events:*  Strictures: 2/16 patients (12.5%) | 4 |
| Bowers SP, et al. (2003)[84] | BE | Cohort study  Single centre  *Countries:* US  *Length of follow-up:* approx. 5 years | *Number of patients:* 30  (Laser Ablation: 9 patients; Surveillance: 21 patients)  Laser ablation  *Number of patients:* 9  *Gender:*  Male: 7  Female: 2  *Age:*  Median: 49 yrs  Range: 33 to 62 yrs  *Number of patients with BE <3cm:* 3/9 (33.3%)  Surveillance  *Number of patients:* 21  *Gender:*  Male: 13  Female: 8  *Age:*  Median: 49 years  Range: 31 to 73 years  *Number of patients with BE <3cm:* 8/21 (38.1%)  *Prior treatments:* None reported  *Inclusion criteria:* none notable    *Exclusion criteria:* none notable | Laser ablation  *Type:* KTP laser @ 532nm  *Pulse time:* not reported  *Power:* 5W  *Dose:* not reported  *Treatment time:* not reported  *Number of sessions:*  Median: 2 sessions / patient  Range: 1 to 5 sessions  *Co-interventions:*  ARS | *Outcomes:*  CR of BE (defined as no endoscopic or histological signs of BE, assessed through endoscopy with 4 quadrant biopsies)  CR of BE (defined as no histological signs of BE, despite columnar-appearing epithelium)  *Adverse events:* none | *Outcomes*  CR of BE:  Laser ablation  -at 3 months: 2/9 patients (22.2%)  -at 61.2 months (mean): 1/9 patients (11.1%)  Surveillance  Not reported  CR of BE:  Laser ablation  -at 3 months: 5/9 patients (55.5%)  -at 61.2 months (mean): 8/9 patients (88.8%)  Surveillance  - at 67.2 months: 7/21 patients (33.3%) | 4 |
| Ertan A, et al. (1995)[85] | BE + HGD | Case report  Single centre  *Countries:* US  *Length of follow-up:* 2 months | *Number of patients:* 1  *Gender:*  Male: 1  *Age:* 80 yrs  *Prior treatments:*  H­­_2_ blockers, unspecified  *Length of Barrett’s:* 14 cm  *Inclusion criteria:* none notable  *Exclusion criteria:* none notable | Laser ablation  *Type:* Nd: YAG  *Power:* not reported  *Dose:*  Mean: 5063.75 J/session  Range: 2761 to 5558 J / session  *Treatment time:* not reported  *Number of sessions:* 8 sessions  Circumferential treatment, for focal HGD only  *Co-interventions:*  OM 40 mg/day | *Outcomes:*  Progression to cancer  *Adverse events:* none | *Outcomes:*  Progression to cancer at 2 months: 1/1 patient (100%) | 4 |
| Fisher RS, et al. (2003)[24] | BE (6 patients)  BE + HGD (3 patients)  BE + LGD (12 patients) *  * 10 additional patients unavailable for analysis | Patient cohort  Single centre  Prospective  *Countries:* US  *Length of follow-up:*  Mean: 19.1 months ± 5.4 months | *Number of patients:* 21  *Gender:*  Male: 21  Female: 10  *Age:*  Mean: 53.6 yrs  Range: 35 to 70 yrs  *Prior treatments:* none reported  *Length of Barrett’s:*  Mean: 4.6 cm ± 0.7 cm  *Inclusion criteria:* none notable  *Exclusion criteria:* none notable | Laser ablation  *Type:* Nd:YAG  *Power:*  Mean: 28.7 ± 3.6 watts  *Dose:*  Mean: 1105 ± 110 J  *Treatment time:* not reported  *Number of sessions:*  Mean: 6.5 sessions / patient ± 1.2 sessions  *Co-interventions:*  PPI, unspecified | *Outcomes:*  CR of BE (assessed through 4 quadrant biopsies)  Recurrence of BE  *Adverse events* | *Outcomes:*  CR of BE  - at <1 month: 21/21 patients (100%)  - at 19.1 months (mean): 13/21 patients (62%)  Recurrence of BE at 19.1 months (mean): 8/21 patients (38%)  *Adverse events:*  Bleeding, requiring transfusions: 1/21 patients (5%)  Perforation, managed conservatively: 1/21 patients (5%)  Strictures, requiring dilation: 1/21 patients (5%) | 4 |
| Norberto L, et al. (2004)[86] | BE (11 patients)  BE + LGD (2 patients)  BE + HGD (2 patients | Patient cohort  Single centre  *Countries:* Italy  *Length of follow-up:*  Mean: 28 months  Range: 7 to 61 months | *Number of patients:* 15  *Gender:*  Male: 13  Female: 2  *Age:*  Mean: 56 yrs  Range: 32 to 73 yrs  *Prior treatments:*  ARS Nissen fundoplication (6 patients)  Gastric resection (1 patient)  Gastric-esophageal resection for previous EAC (1 patient)  *Length of Barrett’s:*  Mean: 4 cm  Range: 1 to 12 cm  *Inclusion criteria:* none notable    *Exclusion criteria:* none notable | Laser ablation  *Type:* Nd: YAG @ 940nm  *Pulse time:* not reported  *Power:* not reported  *Dose:*  Mean: 1705 J/session  Range: 270 to 6135 J/session  *Treatment time:* not reported  *Number of sessions:*  Mean: 6.5 sessions / patient  Range: 2 to 19 sessions  *Cost*: ~$723 USD/session or ~$4692 / person  Half circumference treated / session  *Co-interventions:*  OM 40 mg/day | *Outcomes:*  CR of BE (assessed through endoscopy with jumbo biopsy)  CR of HGD  CR of LGD  Mean % of BE area reduction  *Adverse events* | *Outcomes:*  CR of BE at 28 months (mean): 6/15 patients (40%)  CR of HGD at 28 months (mean): 2/2 patients (100%)  CR of LGD at 28 months (mean): 2/2 patients (100%)  Mean % of BE area reduction: 77%  *Adverse events:*  Strictures: 0/15 patients (0%)  Chest pain, mild: some | 4 |
| Salo JA, et al. (1998)[87] | BE | Cohort study  Single centre  Prospective  Laser ablation + ARS vs. ARS alone  *Countries:* US  *Length of follow-up:*  Laser ablation + ARS Group  Mean: 26 months  Range: 6 to 52 months  ARS Group  Mean: 21 months  Range: 12.5 to 38 months | *Number of patients:* 17  (Laser Ablation + ARS: 11patients; ARS Only: 6 patients)  *Age:*  Mean: 56.6 yrs  Range: 41 to 74 yrs  Laser Ablation + ARS  *Gender:*  Male: 10  Female: 1  *Age:* not reported  *Length of Barrett’s:*  Mean: 4 cm  ARS Only  *Gender*:  Male: 5  Female: 1  *Age:* not reported  *Length of Barrett’s:*  Mean: 8 cm  *Prior treatments:*  PPI  Fundoplication (16 patients)  Roux-en-Y duodenal diversion with partial gastrectomy and gastric vagotomy (1 patient)  *Length of Barrett’s:*  Range: 1 to 11 cm  *Inclusion criteria:* none notable  *Exclusion criteria:* none notable | Laser ablation + ARS  *Type:* Nd:YAG  *Power:*30 watts  *Dose:*  Range: 300 to 4000 J / session  *Treatment time:* maximum 40 minutes  *Number of sessions:*  Mean: 4 sessions / patient  Range: 1 to 8 sessions  Preceded by ARS (fundoplication)  ARS  Fundoplication, various techniques  *Co-interventions:* none reported | *Outcomes:*  CR of BE (assessed through endoscopy with 4 quadrant biopsies every 1 cm)  Number of laser sessions to achieve CR of BE  *Adverse events:* none | *Outcomes:*  CR of BE:  Laser ablation + ARS Group  - at 3 months: 11/11 patients (100%)  - at 26 months (mean): 11/11 patients (100%)  ARS Group  - at 3 months: 0/6 patients (0%)  - at 21 months (mean): 0/6 patients (0%)  Number of laser sessions to achieve CR of BE:  Mean: 4 sessions  Range: 1 to 8 sessions | 4 |

***Note:*** ARS (anti-reflux surgery), BE (Barrett’s esophagus), CR (complete response), EAC (esophageal adenocarcinoma), HGD (high grade dysplasia), KTP (potassium titanyl phosphate laser), LGD (low grade dysplasia), Nd:YAG (neodymium doped yttrium aluminum garnet laser), OM (omeprazole), PPI (proton pump inhibitor)
