# Supplementary material for: Endoscopic treatments for Barrett's esophagus: a systematic review of safety and effectiveness compared to esophagectomy
Source: BMC Gastroenterol. 2010 Sep 27;10:111. doi: 10.1186/1471-230X-10-111 (PMC2955687; doi:10.1186/1471-230X-10-111)
Supplement: Additional file 6 — Studies of multipolar electrocoaguation (MPEC) for Barrett's esophagus with/without dysplasia. Details of study and patient characteristics, outcomes and study quality of the included studies of MPEC for BE with/without dysplasia are presented in Additional file 6. [file 1471-230X-10-111-S6.DOC]

| **Study authors (year published)**  Additional file 6. Studies of multipolar electrocoagulation (MPEC) for Barrett's esophagus with/without dysplasia | **Cancer / Cell Type** | **Study Design** | **Patients** | **Intervention** | **Outcome Measures** | **Findings** | **Study quality** |
| --- | --- | --- | --- | --- | --- | --- | --- |
| *Comparative studies* | | | | | | | |
| Dulai GS, et al. (2005)[17] | BE | RCT  Prospective  MPEC vs. APC  *Countries:* US  *Length of follow-up:* 1 to 1.5 months (after last session) | *Number of patients:* 52  (APC Group: 26 patients; MPEC Group: 26 patients)  MPEC Group  *Gender:*  Male: 23  Female: 3  *Age:*  Mean: 56 yrs ± 11 yrs  APC Group  *Gender*  Male: 21  Female: 5  *Age:*  Mean: 58 yrs ± 11 yrs  *Prior treatments:* none reported  *Length of Barrett’s:*  MPEC Group  Mean: 3.1cm ± 1.7 cm  APC Group  Mean: 4.0 cm ± 1.5 cm  *Inclusion criteria:* none notable  *Exclusion criteria:*  Severe active comorbid disease  Diagnosis of HGD or cancer  Prior antireflux surgery  Inability to discontinue NSAID therapy  Pregnancy, lactation or non-use of birth control measures  Allergy to PPI  Uncontrolled coagulopathy | MPEC vs. APC  MPEC Group  *Probe:* not reported  *Power:* 16 watts  *Treatment time:* not reported  *Number of sessions:*  Mean: 2.9 sessions ± 1.5 sessions  APC Group  *Gas flow*: 2L/minute  *Power:* 60 watts  *Treatment time*: not reported  *Number of sessions:*  Mean: 3.8 sessions ± 1.7 sessions  *Co-interventions:*  Pantoprazole, dosing unspecified. | *Outcomes:*  CR of BE (assessed through endoscopy with 4 quadrant biopsies every 2 cm)  *Adverse events:* | *Outcomes:*  CR of BE at 1 to 1.5 months:  -MPEC Group: 23/26 patients (88%)  -APC Group: 21/26 patients (81%)  (p=0.68)  *Adverse events:*  APC Group  Chest pain, severe: 1/26 patients (4%)  MPEC Group  none | 1 |
| Sharma P, et al. (2006)[18] | BE  BE + LGD | RCT  Multi-centre  Prospective  MPEC vs APC  *Countries:* US  *Length of follow-up:* 2 yrs | *Number of patients*: 35  (MPEC Group: 16 patients; APC Group: 19 patients)  *Gender:*  Male: 34  Female: 1  MPEC Group  *Age*  Mean: 60 yrs  Range: 42 to 68 yrs  APC Group  *Age*  Mean: 65 yrs  Range: 32 to 84 yrs  *Prior treatments:* none reported  *Length of Barrett’s:*  MPEC Group  Mean: 3 cm  Range: 2 to 6 cm  APC Group  Mean: 4 cm  Range: 2 to 6 cm  *Inclusion criteria:* none notable  *Exclusion criteria:*  History of esophageal surgery; HGD with EAC; strictures or varices; allergy to PPI; coagulopathy; significant uncontrolled co-morbidities | MPEC vs. APC  MPEC Group  *Probe*: 10F gold  *Power:* 20 watts  *Number of sessions:* 3.8 sessions / patient  APC Group  *Gas flow:* 1.4 to 1.8 L/minute  *Power:* 60 watts  *Number of sessions:*  Mean: 3.4 sessions/patient  *Co-interventions:*  Rabeprazole 40mg/day (median) | *Outcomes:*  CR of BE (assessed through endoscopy with 4 quadrant biopsies every 2 cm)  Number of sessions to achieve CR of BE  Progression to cancer  Progression to HGD  *Adverse events* | *Outcomes:*  CR of BE at 2 years:  -MPEC Group: 12/16 patients (75%)  -APC Group: 12/19 patients (63%)  Number of sessions to achieve CR of BE:  -MPEC Group:  Mean: 3.8 sessions / patient  -APC Group:  Mean: 3.4 sessions / patient  (p=0.48)  Progression to cancer at 2 years:  -MPEC Group: 0/16 patients (0%)  -APC Group: 0/19 patients (0%)  Progression to HGD at 2 years:  -MPEC Group: 0/16 patients (0%)  -APC Group: 0/19 patients (0%)  *Adverse events:*  MPEC Group  Sore throat: 9/16 patients (56%)  Dysphagia: 5/16 patients (31%)  Chest pain: 6/16 patients (38%)  Epigastric pain: 0 /16 patients (0%)  Fever, low grade: 0 /16 patients (0%)  Stricture: 0 /16 patients (0%)  Perforation: 0 /16 patients (0%)  Bleeding: 0 /16 patients (0%)  APC Group :  Sore throat: 9/19 patients (47%)  Dysphagia: 2/19 patients (11%)  Chest pain: 4/19 patients (21%)  Epigastric pain: 2/19 patients (11%)  Fever, low grade: 1/19 patients (5%)  Stricture: 1/19 patients (5%)  Perforation: 0/19 patients (0%)  Bleeding: 0/19 patients (0%) | 1 |
| *Non-comparative studies* | | | | | | | |
| Faigel DO, et al. (2002)[88] | BE | Clinical trial*  Prospective  Multicentre  *Countries:* US  *Length of follow-up*: 6 months  *trial featured half-esophagus controls but patients went off split esophagus protocol at 9 weeks and split esophagus outcomes are not clear | *Number of patients: 25*  (29 patients enrolled; 4 dropouts not extracted)  *Gender:*  Male: 24  Female: 1  *Age:*  Mean: 58.5 yrs ± 13.5 yrs  *Prior treatmetns:* none reported  Length of Barrett’s:  Mean: 3.1 cm ± 1.8 cm  Range: 2-6 cm  *Inclusion criteria:* none notable  *Exclusion criteria:*  Erosive or ulcerative esophagitis | MPEC  *Probe*: 10F catheter probe  *Power:* 20 to 25 Watts  *Treatment time:* not reported  *Number of treatments*:  Mean: 3 sessions  Range: 2 to 6 sessions  *Co-interventions:*  OM 40 mg twice daily for 1 week prior and throughout study | *Outcomes:*  CR of BE (assessed through 4 quadrant biopsy every 1-2 cm)  *Adverse events:* none | *Outcomes:*  CR of BE at 6 months: 23/25 patients (92%) | 4 |
| Kovacs BJ, (1999)[89] | BE | Clinical trial  Prospective  Multicentre  *Countries:* US  *Length of follow-up:* 18 weeks | *Number of patients:* 27  *Gender:*  Male: 21  Female: 6  *Age:*  *Range:* 33-81 yrs  *Prior treatments:*  Nissen fundoplication (1 patient)  *Length of Barrett’s:*  Mean: 3.4 cm  Range: 2-10 cm  *Inclusion criteria:* none notable  *Exclusion criteria:*  Esophageal varices; esophageal strictures requiring dilation; esophageal ulceration >9mm diameter | MPEC  *Probe:* 7F Gold probe  *Power:* 12-15 Watts  *Treatment time:* not reported  *Number of sessions:*  Mean: 2.5 sessions / patient  Half circumference treated with MPEC, 2-3 cm length/ session  *Co-interventions:*  Lansoprazole 30 mg twice a day 24 hours prior and throughout the study | *Outcomes:*  CR of BE (assessed through endoscopy with biopsies every 2 cm)    CR of BE (assessed *histologically* through biopsy only)  *Adverse events* | *Outcomes:*  CR of BE at 18 weeks: 15/27 patients (56%)  CR of BE at 18 weeks: 22/27 patients (81%)  *Adverse events:*  Dysphagia, transient, odynophagia, chest pain, heart burn: 11/27 patients (41%)  Strictures 1/27 patients (4%) | 4 |
| Montes CG, et al. (1999)[90] | BE | Case series  Single centre  *Countries:* Brazil  *Length of follow-up:* Mean: 21.6 months Range: 18 to 30 months | *Number of patients:*14  *Gender:*  Male: 11  Female: 3  *Age:*  Mean: 45.7 yrs  Range: 13 to 65 yrs  *Prior treatments:*  ARS (Laparoscopic gastric fundoplication)  Ranitidine 300mg/day  Cisapride 0.2mg/kg before meals  *Length of Barrett’s:*  Mean: 4.8 cm +/- 1.39  Range: 3 to 8 cm  *Inclusion criteria:* none notable  *Exclusion criteria:* none notable | MPEC  *Probe:* 7F bipolar  *Power:* 20 Watts  *Treatment time:* not reported  *Number of treatments:*  Mean: 3.7 sessions / patient ± 1.1 sessions  Range: 3 to7 sessions  Half circumference of esophagus treated with MPEC, 2-3 cm length/ session  *Co-interventions:* none reported | *Outcomes:*  CR of BE (assessed through endoscopy with 4 quadrant biopsies every 2 cm)  Number of sessions to achieve CR of BE  *Adverse events* | *Outcomes:*  CR of BE at 21.6 months (mean): 14/14 patients (100%)    Number of sessions to achieve CR of BE:  Mean: 3.7 sessions / patient ± 1.1 sessions  Range: 3 to 7 sessions / patient  *Adverse events:*  Odynophagia, transient: 2/14 patients (14%)  Dysphagia, transient: 1/14 patients (7%) | 4 |
| Sampliner RE (1999)[92] | BE (7 patients)  BE + LGD (4 patients) | Case series  Single centre  *Countries:* USA  *Length of follow-up:* Mean: 36 months | *Number of patients:* 11  *Gender*: not reported  *Age:* not reported  *Prior treatments:* none reported  *Length of Barrett’s:* not reported  *Inclusion criteria:* none notable  *Exclusion criteria:* none notable | MPEC  *Probe:* not reported  *Power:* not reported  *Treatment time:* not reported  *Number of sessions:* not reported  *Co-interventions:* none reported | *Outcomes*  CR of BE (assessed through endoscopy with 4 quadrant jumbo biopsy)    *Adverse events* | *Outcomes:*  CR of BE at 36 months (mean): 8/11 patients (73%)  *Adverse events*  Heartburn, transient, dysphagia, chest pain, mild: 7/11 patients (64%)  Strictures or perforations: 0/11 patients (0%) | 4 |
| Sampliner RE, et al. (1996)[91] | BE | Clinical trial*  Single centre  *Countries:* US  OM vs. MPEC + OM  *Length of follow-up:*  Mean: 12 months  Range: 10 to 18 months  *trial featured half esophagus controls but control region outcomes are not clear | *Number of patients:* 10  *Gender:*  Male: 8  Female: 2  *Age:*  Mean: 61 years  Range: 45 to 76 yrs  *Prior treatments:* None reported  *Length of Barrett’s*:  Mean: 4.7 cm  Range: 2-9 cm  *Inclusion criteria:* none notable  *Exclusion criteria:* none notable | MPEC  *Probe:* 10F gold  *Power:* 50 Watts @ setting 3  *Treatment time:* not reported  *Number of sessions:*  Mean: 2.5 sessions / patient  Range: 2 to 4 sessions  Half circumference treated with MPEC, 2-3 cm length/ session  *Co-interventions:*  20 mg OM twice daily 1 week prior to treatment  Mean: 56 mg/day OM  Range: 40 to 80 mg OM /day | *Outcomes:*  CR of BE (assessed through endoscopy with 4 quadrant biopsy every 2 cm)  Number of sessions to achieve CR of BE  *Adverse events* | *Outcomes:*  CR of BE at 12 months: 10/10 patients (100%)  Number of sessions to achieve CR of BE:  Mean: 2.5 sessions  Range: 2 to 4 sessions  *Adverse events*  5 events / 75 MPEC sessions (7%)  Odynophagia, transient: 2/10 patients (20%)  Dysphagia, transient: 1/10 patients (10%)  Buried glands, transient: 2/10 patients (20%)  Chest pain: 1/10 patients (10%)  Upper GI bleed 2 weeks post MPEC therapy: 1/10 patients (10%) | 4 |

***Note:*** APC (argon plasma coagulation), ARS (anti-reflux surgery), BE (Barrett’s esophagus), CR (complete response), EAC (esophageal adenocarcinoma), HGD (high grade dysplasia), MPEC (multipolar electrocoagulation), OM (omeprazole), PPI (proton pump inhibitor), RCT (randomized controlled trial)
