# Supplementary material for: Endoscopic treatments for Barrett's esophagus: a systematic review of safety and effectiveness compared to esophagectomy
Source: BMC Gastroenterol. 2010 Sep 27;10:111. doi: 10.1186/1471-230X-10-111 (PMC2955687; doi:10.1186/1471-230X-10-111)
Supplement: Additional file 7 — Studies of radiofrequency ablation (RFA) for Barrett's esophagus with/without dysplasia. Details of study and patient characteristics, outcomes and study quality of the included studies of RFA for BE with/without dysplasia are presented in Additional file 7. [file 1471-230X-10-111-S7.DOC]

| **Study authors (year published)**  Additional file 7. Studies of radiofrequency ablation (RFA) for Barrett's esophagus with/without dysplasia | **Cancer / Cell Type** | **Study Design** | **Patients** | **Intervention** | **Outcome Measures** | **Findings** | **Study quality** |
| --- | --- | --- | --- | --- | --- | --- | --- |
| *Comparative studies* | | | | | | | |
| Bumgarner JM, et al. (2008)[93] | BE (patients with dysplasia included, but number of patients with dysplasia not reported) | Cohort study  Multi-centre  Retrospective  RFA vs. PDT  *Countries*: not stated  *Length of follow-up:* not reported | *Number of patients:*  (RFA Group: 103 patients; PDT Group: 122 patients)  *Gender:* not reported  *Age:* not reported  *Prior treatments:* PPI, unspecified  *Length of Barrett’s:* not reported  *Inclusion criteria:* none notable  *Exclusion criteria:* none notable | RFA vs. PDT  RFA  *Device:* not reported  *Power:* 300W  *Dose: n*ot reported  *Treatment time:* Not reported  *Number of sessions:*  Not reported  PDT  *Drug:* not reported  *Dose:* not reported  *Route of administration:* not reported  *Light source*: not reported  *Light dose:* not reported  *Time to photoactivation:* not reported  *Treatment time:* not reported  *Number of sessions:* not reported  *Co-interventions:* PPI, unspecified | *Outcomes:*  Risk ratio  Percentage of BE remaining after initial ablation  *Adverse events* | *Outcomes:*  Relative risk of cure of dysplasia for RFA vs PDT (follow-up unknown): 0.69, (95% CI (0.26, 1.65)  Percentage of BE remaining after initial ablation:  -RFA: 15%  -PDT 30%  *Adverse events:*  Strictures: 18 strictures in 145 patients undergoing repeat endoscopy, Relative risk of strictures for RFA vs PDT: 0.29, 95% CI (0.09, 0.95) | 4 |
| Shaheen NJ, et al. (2009 )[19] | BE+LGD (64 patients)  BE+HGD (63 patients) | RCT  Multi-centre  Prospective  RFA vs. sham procedure  *Countries*: US  *Length of follow-up:* 12 months | *Number of patients:* RFA group: 84 patients (HGD: 42, LGD: 42); control group (sham procedure group): 43 patients (HGD: 21, LGD: 22)    LGD group  *Gender:*  Male: 33 (RFA), 19 (control)  Female: 9 (RFA), 3 (control)  *Age:* Mean±SD: 66.3±1.4 yrs (RFA), 64.6±1.9 yrs (control)  Range: 41-79 yrs (RFA), 45-78 yrs (control)  *Length of  Barrett’s:* Mean±SD: 4.6±0.4 cm (RFA), 4.6±0.5 cm (control)  Range: 0.5 to 8.0 cm (RFA), 0.5 to 8.0 cm (control)  HGD group  *Gender:*  Male: 37 (RFA), 21 (control)  Female: 5 (RFA), 0 (control)  *Age:*  Mean±SD: 65.9±1.4 yrs (RFA), 67.3±1.8 yrs (control)  Range: 49-80 yrs (RFA), 54-80 yrs (control)  *Length of  Barrett’s:* Mean±SD: 5.3±0.3 cm (RFA), 5.3±0.5 cm (control)  Range: 1.0 to 8.0 cm (RFA), 1.0 to 8.0 cm (control)  *Prior treatments:* 7 patients in the ablation group and 4 patients in the control group received endoscopic mucosal resection  *Inclusion criteria:* 18 to 80 yrs old+non-nodular & length of dysplastic BE ≤ 8 cm; aditional requirement for HGD patients: negative results on endoscopic ultrasonography for lymphadenopathy and esophageal-wall abnormalities within 12 months  *Exclusion criteria:* Pregnancy; active esophagitis or stricture precluding endoscope passage; history of esophageal cancer; eophageal varices;  uncontrolled coagulopathy; life expectancy < 2 yrs | RFA vs. sham procedure  RFA  *Device:* circumferential ablation device (HALO^360^, BARRX Medical)  *Power:* 40 W  *Dose:* 12 J/cm^2^  *Treatment time:*  - Circumferential radiofrequency ablation:  Median: 36 minutes  IQR: 29-45  - focal radiofrequency ablation:  Median: 26 minutes  IQR: 19-40  *Number of sessions:*  Mean: 3.5 treatments per patient  Maximum: 4 sessions  sham procedure  Undergoing upper endoscopy and esophageal intubation with a study catheter without ablation  *Co-interventions:*  Esomeprazole: 40 mg twice daily | *Outcomes:*  CR of BE (histologically)  CR of dysplasia (histologically)  Progression (progression of LGD to HGD or esophageal cancer, progression of HGD to esophageal cancer)  *Adverse events* | *Outcomes at 12 months follow up:*  CR of BE  LGD+HGD group: 65/84 patients (77.4%, RFA), 1/43 patients (2.3%, control), p<0.001  LGD group: 34/42 patients (81.0%, RFA), 1/22 patients (4.5%, control), p<0.001  HGD group: 31/42 (73.8%, RFA), 0/21 patients (0, control), p<0.001  CR of dysplasia  LGD+HGD group: 72/84 patients (85.7%, RFA), 9/43 (20.9%, control), p<0.001  LGD group: 38/42 patients (90.5%, RFA), 5/22 patients (22.7%, control), p<0.001;  HGD group: 34/42 patients (81.0%, RFA), 4/21 patients (19.0%, control), p<0.001  Progression to esophageal cancer  HGD group: 1/42 patients (2.4% , RFA), 4/21 patients (19.0%, control), p=0.04  LGD+HGD group: 1/84 patients (1.2%, RFA), 4/43 patients (9.3%, control), p=0.045  *Adverse events for RFA:*  Strictures: 5/84 patients (6.0%)  Possibly or probably related to RFA: 1/84 patients (1.2%) upper gastrointestinal hemorrhage (this patient receiving antiplatelet therapy for heart disease), 1/84 patients (1.2%) chest pain, 1/84 patients (1.2%) chest discomfort and nausea  Perforation: 0  Procedure-related deaths: 0  *Adverse events for control group:* None | 1 |
| *Non-comparative studies* | | | | | | | |
| Eldaif SM, et al. (2009)[94] | BE (25 patients)  BE + LGD (2 patients) | Case series Single centre  Retrospective  *Countries:* US  *Length of follow-up:* 8 weeks | *Number of patients:* 27  *Gender:*  Male: 16  Female: 11  *Age:*  Mean: 53.6 yrs ± 12.5 yrs  *Prior treatments:*  ARS (5 patients)  PPI, unspecified  *Length of Barrett’s:*  Mean: 4.6 cm ± 4.7 cm  BE≤ 3cm (13 patients)  BE 4-6 cm (9 patients)  BE >6 cm (5 patients)  *Inclusion criteria:*  Chronic reflux symptoms  *Exclusion criteria:* none notable | RFA  *Power:* 300W  *Dose:* 12 J/cm^2^  *Treatment time:* not reported  *Number of sessions:* 1 session / patient  Circumferential ablation  *Co-interventions:*  OM 40 mg twice daily for 30 days then 40 mg/kg daily | *Outcomes:*  CR of BE  *Adverse events* | *Outcomes:*  CR of BE at 8 weeks: 25/27 patients (93%)  *Adverse events:*  Dysphagia or strictures: 0/27 patients (0%) | 4 |
| Fleischer DE, et al. (2008)[95] *  * This study is an extension of the study of Sharma VK, et al.,[100] allowing subsequent focal ablation in patients studied in the efficacy phase (62/70 patients enrolled in the extension study). | BE | Clinical trial  Multi-centre  Prospective  *Countries:* US  *Length of follow-up:* 30 months | *Number of patients:* 70 *Gender:*  Male:52  Female: 18  *Age:*  Mean: 55.7 yrs  Range: 26 to 79 yrs  *Prior treatments:* none reported  *Length of Barrett’s:*  Mean: 3.2 cm  Range: 2 to 6 cm  *Inclusion criteria:* none notable  *Exclusion criteria:*  Strictures; esophagitis; esophageal varices; previous radiation, ablation or resection of the esophagus; implantable electrical devices | RFA  *Power:* 40 W  *Dose:*  10J/cm^2^ (circumferential ablation), 12J/cm^2^ (focal ablation)  *Treatment time:*  Mean: 27.7 minutes  Range 23 to 37 minutes  *Number of sessions:*  -Circumferential ablations  Mean: 1.51 sessions / patient  Range: 1 to 2 sessions / patient  -Focal ablations  Mean: 1.87 sessions / patient  -Any ablations  Mean: 3.39 sessions / patient  Circumferential ablations, followed up until 12 months, then focal ablation, followed up until 20 months  *Co-interventions:*  Esomeprazole 40 mg twice daily for 1 month then 40 mg/day for months 2 to 12 | *Outcomes:*  CR of BE (assessed through endoscopy with 4 quadrant biopsies every 1-2 cm)  *Adverse events* | *Outcomes:*  CR of BE  - at 12 months* (after 1.5 sessions / patient): 48/69 patients (70%)  - at 30 months** (after 3.4 sessions/ patient): 60/61 patients (98%)  *Adverse events:*  After 1.5 sessions/ patient*:  Fever: 2/70 patients (3%)  Chest / throat pain: 9/70 patients (13%)  Mucosal scarring, transient: 1/70 patients (1%)  Laceration, superficial: 1/70 patients (1%)  Bleeding, mild: 1/70 patients (1%)  Nausea, transient: 8/70 patients (11%)  Sedation related hypotension: 1/70 patients (1%)  Sedation related airway obstruction: 1/70 patients (1%)  After 1.9 additional sessions/ patient** (extension study)  Chest or throat pain: 1/62 patients (2%)  Nausea and vomiting: 2/62 patients (3%)  Sedation related hypotension: 1/62 patients (2%)  Fever; laceration; bleeding; mucosal scarring; sedation related airway obstruction: 0/62 patients (0%)  * circumferential ablations  ** focal ablations | 4 |
| Ganz RA, et al. (2008)[96] | BE + HGD | Case series  Multi-centre  Retrospective  *Countries:* US  Length of follow-up:  Median: 12 months | *Number of patients:* 142  *Gender:*  Male: 125  Female:17  Age:  Median: 67 yrs  Range 59 to 75  *Prior treatments:*  EMR: 24 patients  *Length of Barrett’s:*  Median 6 cm  Range 3 to 8 cm  *Inclusion criteria:* none notable  *Exclusion criteria:*  Varices; prior esophageal radiation or surgery other than fundoplication | RFA  *Power:* 300W  *Dose:* 24 J/cm^2^  *Treatment time:* not reported  *Number of treatments:*  Median: 1 session / patient  Inter-Quartile Range: 1 to 2 sessions / patient  Circumferential ablation  *Co-interventions:*  PPI, unspecified | *Outcomes:*  CR of dysplasia (assessed through endoscopy with 4 quadrant biopsy every 1 to 2 cm)  *Adverse events* | *Outcomes:*  CR of dysplasia at 3 months: 83/92 patients (90%)  *Adverse events:*  Strictures 1/142 patients (0.7%) | 4 |
| Hernandez JC, et al. (2008)[97] | BE +/- LGD (7 patients)  BE + HGD (3 patients) | Clinical trial  Single centre  Prospective  *Countries:* US  *Length of follow-up:* 12 months | *Number of patients:* 10  *Gender:*  Male: 8  Female: 2  *Age:*  Mean: 62 yrs  Range: 19 to 73 yrs  *Prior treatments*: none reported  *Length of Barrett’s:*  Mean: 4.9 cm  Range: 1 to 11 cm  *Inclusion criteria:* none notable  *Exclusion criteria*  Esphoageal strictures, active esophagitis, esophageal varices, esophageal malignancy, prior esophageal surgery, prior ablation or radiation therapy of the esophagus, comorbid condition affecting compliance | RFA  *Power:* 300W  *Dose:*  Nondysplasia patients: 20 J/cm^2^  Dysplasia patients:36 J/cm^2^  *Treatment time:* Not reported  *Number of sessions:*  Mean: 2.5 sessions / patient  Range: 1 to 3 sessions  Circumferential, then focal ablations to treat residual BE (<2cm)  *Co-interventions:*  PPI, unspecified | *Outcomes:*  CR of BE (assessed through endoscopy with 4 quadrant biopsy every 1 cm)  Partial response of BE (50 to <100% of biopsies negative for BE)  Number of sessions to achieve CR of BE  *Adverse events* | *Outcomes:*  CR of BE at 12 months:  7/10 patients (70%)  Partial response of BE at 12 months:  3/10 patients (30%)  Number of sessions to achieve CR of BE:  Mean: 1.4 sessions/ patient  *Adverse events:*  Throat and chest pain, mild: common | 4 |
| Hubbard N & Velanovich V (2007)[98] | BE | Case series  Single centre  Prospective  *Countries*: US  *Length of follow-up:* 3 months | *Number of patients:* 7  *Gender:*  Male: 5  Female: 2  *Age:*  Mean: 60.57 yrs  Range: 41 to 78 yrs  *Prior treatments:*  Fundoplication  *Length of Barrett’s:*  Mean: 4.43 cm  Range: 1 to 12 cm  *Inclusion criteria:*  Previous fundoplication  *Exclusion criteria:* none notable | RFA  *Power:* 300W  *Dose:* not reported  *Treatment time:* not reported  *Number of sessions:* not reported  Circumferential ablation  *Co-interventions:* none reported | *Outcomes:*  CR of BE (assessed by endoscopy)  *Adverse events*: none | *Outcomes:*  CR of BE at 3 months: 6/7 patients (86%) | 4 |
| Pouw RE, et al. (2008)[99] | BE (2 patients)  LGD (10 patients)  HGD (32 patients) | Clinical trial  Multi-centre    *Countries:* Netherlands, other non-reported European countries  *Length of follow-up*:  Mean: 21 months  Range: 10 to 27 months | *Number of patients:* 44  *Gender:*  Male: 35  Female: 9  *Age:*  Mean: 68 yrs  Range: 57 to 75 yrs  *Prior treatments:*  Focal EAC or HGD by EMR (39 patients)  *Length of Barrett’s:*  Median: 7cm  Range: 4 to 9 cm  *Inclusion criteria:*  *Exclusion criteria:*  Esophageal stenosis | RFA  *Source:* Balloon-based radiofrequency electrode  *Power:* 40 watts  *Dose:* 12 J/cm^2^  *Treatment time:* not reported  *Number of sessions:*  Mean: 3 sessions (1 circumferential + 2 focal ablations)  *Co-interventions:*  Esomeprazole 40 mg twice daily  Ranitidine 300 mg at bedtime  Sucralfate 2 mL @ 200 mg/mL 4 times a day | *Outcomes:*  CR of BE at 2 months post treatment (assessed though endoscopy with 4 quadrant biopsies every 1-2 cm)  Progression to cancer    *Adverse events:* | *Outcomes:*  CR of BE at 2 months post treatment: 43/44 patients (98%)  Progression to cancer after a mean of 21 months follow-up:  1/44 patients (2%)  *Adverse events:*  Laceration, superficial, at sites of previous EMR scars: 3/44 patients (7%)  Dysphagia: 4/44 patients (9%)  Fever: 1/44 patients (2%)  Chest pain: 2/44 patients (4%) | 4 |
| Roorda AK, et al. (2007)[100] | BE (6 patients)  BE + HGD (3 patients)  BE + LGD (4 patients) | Case series  Single centre  *Countries:* US  *Length of follow-up:*  Mean: 12 months  Range: 6 to 19 months | *Number of patients:* 13  *Gender:*  Male: 12  Female: 1  *Age:*  Mean: 57 yrs  Range: 31 to 75 yrs  *Prior treatments:*  Fundoplication (2 patients)  PPI  EMR (1 patient)  *Length of Barrett’s:*  >3cm: 10 patients  <3 cm: 3 patients  *Inclusion criteria:*  Patients with GERD  *Exclusion criteria:* none notable | RFA  *Power:* 300 watts  *Dose:*  -BE: 20 J/cm^2^ (6 patients)  -BE + dysplasia: 24 J/cm^2^ (7 patients)  *Treatment time:* not reported  *Number of sessions:*  Mean: 1.4 sessions  Range: 1 to 2 sessions  Circumferential ablation  *Co-interventions:*  PPI, unspecified | *Outcomes:*  CR of BE (assessed through endoscopy with 4 quadrant biopsies every 1-2 cm)  CR of dysplasia  *Adverse events* | *Outcomes:*  CR of BE at 12 months: 6/13 patients (46%)  CR of dysplasia at 12 months: 5/7 patients (71%)  *Adverse events:*  Fever, low grade: 1/13 patients (8%)  Dysphagia, mild; and odynophagia: 3/13 patients (23%)  Strictures or buried glands: 0/13 patients (0%) | 4 |
| Sharma VK, et al. (2007)[101]  Data of the dosimetry phase were reported in Additional files 9 and 10, but data of the effectiveness phase were not included, because patients in the effectiveness phase were the same as those in the study of Fleischer DE, et al.[95] | BE | Clinical trial  Multi-centre  Prospective  *Countries:* US  *Length of follow-up:* 12 months | Dosimetry phase  *Number of patients:* 32  *Gender:*  Male: 29  Female: 3  *Age:*  Mean: 56.8 yrs  Range: 35 to 75 yrs  *Prior treatments:* none reported  *Length of Barrett’s:*  Mean: 2.3 cm  Range: 1 to 4 cm  Effectiveness phase  *Number of patients:* 70  *Gender:*  Male: 52  Female: 18  *Age:*  Mean: 55.7 yrs  Range: 26 to 79 yrs  *Prior treatments:* none reported  *Length of Barrett’s:*  Mean: 3.2 cm  Range: 2 to 6 cm  *Inclusion criteria:* none notable  *Exclusion criteria:*  Strictures; esophagitis; esophageal varices; previous radiation, ablation or resection of the esophagus; implantable electrical devices | Dosimetry phase  RFA  *Device:* HALO^360^ system (BARRX Medical, Inc., Sunnyvale, CA)  *Power:* 300W  *Dose:*  6, 8, 10, or 12 J/cm^2^  *Treatment time:*  Median: 24 minutes  IQR: 20 to 35 minutes  *Number of sessions:*  Range: 1 to 2 sessions  Effectiveness phase  RFA  *Device:* HALO^360^ system (BARRX Medical, Inc., Sunnyvale, CA)  *Power:* 300W  *Dose:*  10 J/cm^2^ (2×)  *Treatment time:*  Median: 28 minutes  IQR: 24 to 33 minutes  *Number of sessions:*  Range: 1 to 2 sessions  Circumferential ablation  *Co-interventions:*  Esomeprazole: 40 mg twice a day for 1 month post ablation; 40 mg every second day for follow-up months 2-12 | *Outcomes:*  CR of BE (assessed through endoscopy with 4 quadrant biopsies every 1-2 cm, CR defined as all biopsy fragments negative for BE)  Partial response of BE (50 to 99% of biopsy fragments negative for BE)  *Adverse events* | Dosimetry phase  *Outcomes:*  CR of BE:  - at 3 months: 7/32 patients (22%)  - at 12 months: 19/32 patients (59%)  Partial response of BE:  - at 3 months: 19/32 patients (59%)  - at 12 months: 8/32 patients (25%)  *Adverse events:*  Chest pain: 3/32 patients (9%)  Mucosal scarring, transient: 1/32 patients (3%)  Lacerations, superficial: 1/32 patients (3%)  Effectiveness phase  *Outcomes:*  CR of BE:  - at 12 months: 48/70 patients (69%)  Partial response of BE:  - at 12 months: 17/70 patients (24%)  *Adverse events:*  Fever: 2/70 patients (3%)  Chest pain: 9/70 patients (13%)  Lacerations, superficial: 1/70 patients (1%)  Mild bleeding during ablation: 1/70 patients (1%)  Mucosal scarring, transient: 1/70 patients (1%)  Sedation-related transient airway obstruction: 1/70 patients (1%)  Sedation-related hypotension: 1/70 patients (1%)  Transient nausea: 8/70 patients (11%) | 4 |
| Smith CD, et al. (2007)[102] | HGD | Clinical trial  Multi-centre  Prospective  Countries: US  Length of follow-up: immediate pathologic outcomes only | *Number of patients: 5*  *Gender:*  Male: 5  Female: 0  *Age:*  Mean: 57 yrs  Range: 45 to 71 yrs  *Prior treatments*  PPI, unspecified  *Length of Barrett’s*:  Mean: 7 cm  Range: 4 to 10 cm  *Inclusion criteria:*  Consent to esophagectomy post RFA  *Exclusion criteria:*  Esophageal strictures  Previous ablative therapy | RFA  *Power:* 300W  *Dose:* 20 to 56 J/cm^2^  *Treatment time:*  Mean: 31 minutes  Range: 11 to 65 minutes  *Number of sessions:*  1 session / patient  Circumferential ablation  *Co-interventions:*  All sessions followed by esophagectomy | *Outcomes:*  CR of BE (assessed by pathological assessment of esophagectomy specimens post-RFA)  *Adverse events:* none | *Outcomes:*  CR of BE at immediate follow-up: 9/10 ablation regions (90%) | 4 |
| Sharma VK, et al. (2009)[103] | BE+LGD (39 patients)  BE+HGD (24 patients) | Clinical trial  Single centre  Prospective  Countries: US  *Length of follow-up:*  Median: 21 months  Range: 3 to 46 | *Number of patients:* 63  *Gender:*  Male: 57  Female: 6  *Age:*  Median: 71 yrs  Range: 43 to 83  *Previous treatment:*  none reported  *Length of Barrett’s*:  Median: 5 cm  Range: 1 to 13 cm  *Inclusion criteria:* none notable  *Exclusion criteria:*  Prior ablative therapy for BE | RFA  *Device:*  Circumferential ablation:HALO^360^ system (BARRX Medical, Inc., Sunnyvale, CA)  Focal ablation: HALO^90^ system  *Power:* 40 W  *Dose:* 12 J/cm^2^  *Treatment time:* none reported  *Number of sessions:*  Median: 1 circumferential ablation , 1 focal ablation  Range: 1 to 4 (circumferential ablation for LGD), 0 to 4 (circumferential ablation for HGD), 0 to 2 (focal ablation for LGD), 0 to 3 (focal ablation for HGD)  circumferential ablation first, focal ablation used in the last year  *Co-interventions:*  High-dose PPI (i.e., esomeprazole 40mg bid) until eradication of BE and dysplasia, then baseline PPI | *Outcomes:*  CR of BE (all biopsy fragments negative for BE at the last endoscopy)  CR of dysplasia (all biopsy fragments negative for dysplasia at the last endoscopy)  *Adverse events* | *Outcomes:*  CR of BE:  - at 24 months (median): 33/39 patients with LGD (85%)  - at 23 months (median): 16/24 patients with HGD (67%)  CR of dysplasia:  CR of LGD:  - at 24 months (median): 38/39 patients with LGD (97%)  CR of HGD:  - at 23 months (median): 24/24 patients with HGD (100%)  *Adverse events:*  Minor bleeding: 1/63 patients (1.6%)  Stricture: 1/63 patients (1.6%) | 4 |
| Vassiliou MC, et al. (2009)[104] | Intramucosal carcinoma (3 patients)  BE+HGD (15 patients)  BE+LGD (6 patients)  BE (1 patient) | Case series  Single centre Retrospective  *Countries:* Canada  *Length of follow-up:*  Median: 20.3 months  IQR: 10.4-29.2 months | *Number of patients:* 25  *Gender:*  Male:22  Female: 3  *Age:*  Median: 66 yrs  IQR: 57 to 74 yrs  *Prior treatments:* 3 patients with intramucosal carcinoma  received cap-based EMR prior to ablation  *Length of Barrett’s:*  Median: 10 cm  IQR: 8 to 12 cm  *Inclusion criteria:*  Length of Barrett’s ≥ 8 cm at the time of first ablation  *Exclusion criteria:*  None reported | RFA  *Device:* Halo 360 or Halo 90, or both (BARRX Medical, Inc., Sunnyvale, CA)  *Power:* none reported  *Dose:*  10 J/cm^2^ (BE patients), 12 J/cm^2^ (LGD and HGD patients)  *Treatment time:*  none reported  *Number of sessions:*  Median: 2.5 ablations for 14 patients with CR of BE  IQR: 2-3 ablations for 14 patients with CR of BE  *Co-interventions:*  PPI twice daily | *Outcomes:*  CR of BE (CR defined as eradication of all intestinal metaplasia in all biopsies)  CR of dysplasia (CR defined as eradication of all intestinal metaplasia in all biopsies)  *Adverse events* | *Outcomes:*  CR of BE  - at 20.3 month: 11/14 patients (78.6%)  CR of HGD  -- at 20.3 month: 8/10 patients (80.0%)  *Adverse events*  (59 ablation procedures in 25 patients)  Hemorrhage (mild self-limited): 1/59 ablations (1.7%)  Stricture: 2/59 ablations (3.4%)  Nausea and vomiting: 2/59 ablations (3.4%) | 4 |
| Velanovich V (2009)[105] | BE (54 patients, LGD could be included)  BE+HGD (12 patients) | Case series  Single centre Prospective  *Countries:* US  *Length of follow-up:*  Maximum: 32 months | *Number of patients:* 66  *Gender:*  Male:53  Female: 13  *Age:*  Mean: 62 yrs  *Prior treatments:* none reported  *Length of Barrett’s:*  Median: 3 cm  Range: 1 to 14 cm  *Inclusion criteria:*  Biopsy confirmed Barrett’s metaplasia without evidence of adenocarcinoma  *Exclusion criteria:*  Patients with invasive adenocarcinoma or carcinoma in situ | RFA  *Device:* BARRX system  (BARRX Medical, Inc., Sunnyvale, CA)  *Power:* none reported  *Dose:*  10 J/cm^2^  *Treatment time:*  none reported  *Number of sessions:*  none reported  (Residual Barrett’s reablated)  *Co-interventions:*  PPI twice daily, sucralfate slurry four times daily, and acetaminophen with codeine elixir for pain | *Outcomes:*  CR of BE  CR of dysplasia  *Adverse events* | *Outcomes:*  CR of BE  - at 3 months:  CR: 29/49 patients (59.2%)  - at 12 months (median):  CR: 25/27 patients (92.6%)  CR of HGD:  - at 19 months (median):  CR: 7/12 patients (58.3%)  *Adverse events*  Within 2 weeks procedure-related complications: 0/66 patients, except strictures: 4/66 patients (6.1%) | 4 |
| Gondrie JJ, et al. (2008)[106] | BE+LGD (2 patients)  BE+HGD (9 patients) | Clinical trial  Single centre  Prospective  Countries: Netherlands  *Length of follow-up:*  Median: 19 months | *Number of patients:* 11  *Gender:*  Male: 8  Female: 3  *Age:*  Median: 60 yrs  Range: 57 to 67  *Previous treatment:*  6/11 patients underwent endoscopic resection of visible lesions  *Length of Barrett’s*:  Median: 5 cm  IQR: 4 to 7 cm  *Inclusion criteria:* none notable  Length of BE between 2 and 10 cm  HGD or IMC  Patients with visible lesions undergoing focal endoscopic resection prior to ablation and documented presence of residual LGD or HGD after endoscopic resection  Age between 18 and 85 yrs  Written informed consent  *Exclusion criteria:*  Vertical resection margin positive for cancer, submucosal invading cancer, poorly differentiated cancer, or presence of lymphatic/ vascular invasion after endoscopic resection  Significant esophageal stenosis  Visible lesions or invasive cancer or absence of residual dysplasia after endoscopic resection but prior to ablation | RFA  *Device:*  Circumferential ablation:HALO^360^ system (BARRX Medical, Inc., Sunnyvale, CA)  Focal ablation: HALO^90^ system  *Power:* 40 W  *Dose:* 12 J/cm^2^ (circumferential ablation), 12 to 15J/cm^2^ (focal ablation)  *Treatment time:* none reported  *Number of sessions:*  2 circumferential ablations (11 patients), 1 focal ablation (2 patients), 2 focal ablations (5 patients), 3 focal ablations (4 patients)  Notes: circumferential ablation first, then focal ablation  *Co-interventions:*  Esomeprazole 40 mg bid throughout the entire study period, and ranitidine 300 mg at bedtime+ 5ml sucralfate suspension 200 mg/ml qid for 2 weeks after each treatment endoscopy | *Outcomes:*  CR of BE (complete endoscopic and histological eradication of BE)  CR of dysplasia (complete histological clearance of dysplasia )  *Adverse events* | *Outcomes:*  CR of BE:  - at 4 months: 2/11 patients (18.2%)  - at 10 months: 7/11 patients (63.6%)  - at 16 months: 10/11 patients (90.9%)  - at 19 months: 11/11 patients (100%)  CR of dysplasia:  - at 4 months: 10/11 patients (90.9%)  - at 10 months: 10/11 patients (90.9%)  - at 16 months: 11/11 patients (100%)  - at 19 months: 11/11 patients (100%)  *Adverse events:*  Fever: 1/11 patients (9.1%)  Chest pain: 1/11 patients (9.1%)  Retrosternal pain: 1/11 patients (9.1%) | 4 |
| Gondrie JJ, et al. (2008)[106] | BE+LGD (1 patients)  BE+HGD (11 patients) | Clinical trial  Single centre  Prospective  Countries: Netherlands  *Length of follow-up:*  Median: 14 months | *Number of patients:* 12  *Gender:*  Male: 9  Female: 3  *Age:*  Median: 70 yrs  Range: 53 to 76  *Previous treatment:*  7/12 patients underwent endoscopic resection of visible lesions  *Length of Barrett’s*:  Median: 7 cm  IQR: 6.5 to 8 cm  *Inclusion criteria:*  Length of BE between 2 and 10 cm  HGD or IMC  Patients with visible lesions undergoing focal endoscopic resection prior to ablation and documented presence of residual LGD or HGD after endoscopic resection  Age between 18 and 85 yrs  Written informed consent  *Exclusion criteria:*  Vertical resection margin positive for cancer, submucosal invading cancer, poorly differentiated cancer, or presence of lymphatic/ vascular invasion after endoscopic resection  Significant esophageal stenosis  Visible lesions or invasive cancer or absence of residual dysplasia after endoscopic resection but prior to ablation | RFA  *Device:*  Circumferential ablation:HALO^360^ system (BARRX Medical, Inc., Sunnyvale, CA)  Focal ablation: HALO^90^ system  *Power:* 40 W  *Dose:* 12 J/cm^2^ (circumferential ablation), 12 to 15J/cm^2^ (focal ablation)  *Treatment time:* none reported  *Number of sessions:*  1 circumferential ablation (11 patients), 2 circumferential ablation s (1 patient), 2 focal ablations (8 patients), 3 focal ablations (4 patients)  Notes: circumferential ablation first, then focal ablation  *Co-interventions:*  Esomeprazole 40 mg bid and ranitidine 300 mg at bedtime+ 5ml sucralfate suspension 200 mg/ml qid for 2 weeks after each treatment endoscopy | *Outcomes:*  CR of BE (complete endoscopic and histological eradication of BE)  CR of dysplasia (complete histological clearance of dysplasia )  *Adverse events* | *Outcomes:*  CR of BE:  - at 2 months: 8/12 patients (66.7%)  - >6 months and <14 months: 12/12 patients (100%)  - at 14 months: 12/12 patients (100%)  CR of dysplasia:  - at 2 months: 11/12 patients (91.7%)  - >6 months and <14 months: 12/12 patients (100%)  - at 14 months: 12/12 patients (100%)  Notes:  CR of HGD:  - at 2 months: 10/11 patients (90.9%)  - >6 months and <14 months: 11/11 patients (100%)  CR of LGD:  - at 2 months: 1/1 patients (100%)  *Adverse events:*  Dysphagia (due to endoscopic resection and ablation): 1/12 patients (8.3%) | 4 |

***Note:*** ARS (anti-reflux surgery), BE (Barrett’s esophagus), CR (complete response), EAC (esophageal adenocarcinoma), EMR (endoscopic mucosal resection), GERD (gastroesophageal reflux disease), HGD (high grade dysplasia), IMC (intramucosal cancer), IQR (interquartile range), LGD (low grade dysplasia), OM (omeprazole), PDT (photodynamic therapy), PPI (proton pump inhibitor), RFA (radiofrequency ablation)
