# Supplementary material for: Endoscopic treatments for Barrett's esophagus: a systematic review of safety and effectiveness compared to esophagectomy
Source: BMC Gastroenterol. 2010 Sep 27;10:111. doi: 10.1186/1471-230X-10-111 (PMC2955687; doi:10.1186/1471-230X-10-111)
Supplement: Additional file 8 — Studies of esophagectomy for Barrett's esophagus with/without dysplasia. Details of study and patient characteristics, outcomes and study quality of the included studies of esophagectomy for BE with/without dysplasia are presented in Additional file 8. [file 1471-230X-10-111-S8.DOC]

| **Study authors (year published)**  Additional file 8. Studies of esophagectomy for Barrett's esophagus with/without dysplasia | **Cancer / Cell Type** | **Study Design** | **Patients** | **Intervention** | **Outcome Measures** | | **Findings** | **Study quality** |
| --- | --- | --- | --- | --- | --- | --- | --- | --- |
| *Comparative studies* | | | | | | | | |
| Prasad GA, et al. (2007)[107] | BE + HGD | Cohort Study  Single centre  Retrospective    Esophagectomy vs PDT  *Countries:* United States  *Length of follow-up:* 5 yrs | *Number of patients:* 199  (Esophagectomy Group: 70 patients; PDT Group: 129 patients)  Esophagectomy Group  *Gender:*  Male: 61  Female: 9  *Age:*  Mean: 60.5 yrs ± 10.8 yrs  PDT Group  *Gender:*  Male: 121  Female: 8  *Age:* 64.5 yrs ± 10.2 yrs  *Prior treatments:* none reported  *Length of Barrett’s:*  PDT Group  Median: 5 cm  Range 3 to 8.5 cm  Esophagectomy Group  Median: 5 cm  Range: 5 to 10.5 cm  *Inclusion criteria:* none notable  *Exclusion criteria:* none notable | Esophagectomy vs. PDT  Esophagectomy Group  TTE or THE  PDT Group  *Drug and dose::*  HpD 4 mg/kg – 26 patients  Porfimer sodium 2 mg/kg – 103 patients  *Route of administration:* IV  *Time to photoactivation:* 48 hours  *Light source:* laser (type not reported )at 630nm  *Light dose:* 200J/cm^2^  *Treatment time:* not reported  *Number of sessions / patient:*  Mean: 1.26 sessions / patient  Range: 1 to 2 sessions / patient  *Co-interventions:*  PPI, unspecified.  EMR for focally visible lesions on endoscopy | *Outcomes:*  CR of dysplasia  Mortality:  -All cause  -Cancer  Progression to cancer  Mortality, hazard ratio*, PDT vs. esophagectomy  -Overall  -Cancer free  *Adverse events*  * Cox proportional hazards model | *Outcomes:*  CR of dysplasia:  Esophagectomy Group  Not recorded  PDT Group  - at 1 year: 88%  - at 3 years: 86%  Mortality at 5 years:  Esophagectomy Group  -All cause: 6/70 patients (8.5%)  -Cancer: 0/70 patients (0%)  PDT Group  -All cause: 11/129 patients (9%)  -Cancer: 0/129 patients (0%)  Progression to cancer:  Esophagectomy Group  Not recorded  PDT Group  - at 1 year: 6/129 patients (5%)  - at 3 years: 8/129 patients (6%)  Mortality, hazard ratio, at 5 years:  -Overall: 1.31 (95% CI [0.4, 4.17])  -Cancer free: 2.45 (95% CI [0.85, 7.12])  *Adverse events:*  Esophagectomy Group  Stricture: 9/70 patients (13%)  Photosensitivity: 0/70 patients (0%)  Post-op mortality: 1/70 patients (1%)  Total post-op “morbidity”: 27/70 patients (39%)  PDT Group *  Stricture: 35/131 patients (27%)  Photosensitivity: 77/131 patients (59%)  Post-op mortality: 0/131 patients (0%)  Total post-op “morbidity”: none  * 3 extra patients of unknown origin reported. | | 2 |
| Reed MF, et al. (2005)[20] | BE + HGD | Cohort study  Single centre  Retrospective  Esophagectomy vs Endoscopic Therapy vs Observation  *Countries:* not reported  *Length of follow-up*: 10 yrs | *Number of patients: 115*  (Esophagectomy Group: 49 patients; Endoscopic Therapy Group: 47 patients; Observations Group: 19 patients)  *Age:*  Mean 65 yrs  Range 30 to 87 yrs  Gender:  Male: 95  Female: 20  Esophagectomy Group  *Age:*  Mean 59 yrs  Range 32 to 79 yrs  *Gender:*  Male: 40  Female: 9  Endoscopic Group  *PDT: 42 patients*  *EMR 5 patients*  *Age:*  Mean 70 yrs  Range 30 to 89 yrs  *Gender:*  Male: 38  Female: 9  Observation Group:  *Age:* not reported  *Gender:* not reported  *Prior treatments:* none reported  *Length of Barrett’s:* not reported  *Inclusion criteria*: none notable  *Exclusion criteria*: none notable | Esophagectomy Group:  Surgical resection done within 60 days of diagnosis  *Type of surgery:*  -TTE: 20 patients (41%)  -Ivor Lewis: 18 patients (37%)  -THE: 7 patients (17%)  -various or mixed techniques: 4 patients(8%)  Endoscopic Group  EMR or PDT)  No details reported  Observation Group:  No details reported  *Co-interventions:* none reported | *Outcomes:*  Disease specific survival  Overall survival  CR of HGD  Progression to cancer  *Adverse event:* | *Outcomes:*  Disease specific survival at 5 years:  -Esophagectomy Group: 94%  -Endoscopic Group: not reported  -Observation Group: not reported  Overall survival:  Esophagectomy Group  - at 5 yrs: 83%  - at 10 yrs: 64%  Endoscopic Group: not reported  Observation Group: not reported  CR of HGD, follow-up unknown:  Esophagectomy Group  not reported  Endoscopic Group  PDT: 37/42 patients (88%)  EMR 3/5 patients (60%)  Observation Group  0/13 patients (0%)  Progression to cancer  -Esophagectomy Group: not reported  -Endoscopic Group: 6/47 patients  -Observation 7/13 patients  *Adverse events:*  Esophagectomy Group  Post op anastomotic leak: 2/49 patients (4%)  Death secondary to large cerebrovascular accident post-op: 1/49 patients (2%) | | 4 |
| Thomas T, et al. (2005)[55] *  * Information extracted for BE or HGD patients only | BE + HGD | Cohort study  Multi-centre  Retrospective  Esophagectomy vs APC vs Non-Intervention Surveillance vs  *Countries:* UK  *Length of follow-up:*  Esophagectomy Group  Mean: 21 months  Range: 6 to 36 months  APC and Non-Intervention Groups  Not reported Surveillance Group  Mean: 15 months  Range: 4 to 39 months | *Number of patients:* 27  (Esophagectomy Group: 8 patients; APC: 5 patients; Non-Intervention Group: 7 patients; Surveillance Group: 7 patients)  Esophagectomy Group  *Gender:*  Male: 7  Female: 1  *Age:*  Mean: 58 yrs  Range: 46 to 76 yrs  APC Group  *Gender:*  Male: 5  *Age:*  Mean: 70 yrs  Range: 54 to 76 yrs  Non-Intervention Group  *Gender:* not reported  *Age:*  Mean: 80 yrs  Range: 74 to 95 yrs  Surveillance Group  *Gender:*  Male: 6  Female: 1  *Age:*  Mean: 65.4 yrs  Range: 55 to 86 yrs  *Prior treatments:* PPI, unspecified  *Length of Barrett’s:*  Mean: 6 cm  Range: 3 to 14 cm  APC Group  Mean: 6 cm  Range: 3 to 9 cm  Surveillance Group  Mean: 5 cm  Range: 2 to 10 cm  *Inclusion criteria:* none notable  *Exclusion criteria:* none notable | Surveillance vs Esophagectomy vs APC vs Non-Intervention  Esophagectomy Group  No details reported  APC Group  *Gas flow:* not reported  *Power:* not reported  *Treatment time:* not recorded  *Number of sessions:*  Mean: 4 sessions / patient  Range: 1 to 14 sessions / patient  Surveillance Group  *Time between endoscopies:*  Mean: 4.6 months  *Number of treatments:*  Mean 2.9 treatments / patient  Range: 1 to 5 treatments / patient  4 quadrant biopsy every 2 cm in 45% of biopsies  *Co-interventions:*  OM 20-40 mg daily: 17 patients  Lansoprazole 30 mg daily: 14 patients  Pantoprazole 40 mg daily: 1 patient  Rabeprazole 40 mg daily: 2 patients  Ranitidine 150 mg twice daily: 3 patients | *Outcomes:*  Overall survival  Disease specific survival  CR of dysplasia  Progression to cancer  *Adverse events:* No HGD or BE specific information availble | *Outcomes:*  Overall survival:  -Esophagectomy Group at 21 months (mean): 5/8 patients (62.5%)  -APC Group: not reported  -Non-Intervention Group, at unknown follow-up: 2/7 patients (28.6%)  -Surveillance Group: not reported  Disease-specific survival  -Esophagectomy Group at 21 months (mean): 7/8 patients (88%)*  -APC Group: not reported  -Non-Intervention Group, at unknown follow-up: 5/7 patients (71%)**  -Surveillance Group: not reported  CR of dysplasia:  -Esophagectomy Group: not reported  -APC Group at unknown follow-up: 2/5 patients (40%)  -Non-intervention Group: not reported  -Surveillance Group at 15 months (mean): 4/7 patients (57%)  Progression to cancer:  Progression to cancer:  -Esophagectomy Group at 21 months (mean): 2/8 patients (25%)  -APC Group at unknown follow-up: 2/5 patients (40%)  -Non-Intervention Group at unknown follow-up: 2/4 patients (50%)  -Surveillance Group at 15 months: 2/6 patients (33%) | | 4 |
| *Non-comparative studies* | | | | | | | | |
| Ferguson MK, et al. (1997)[108] | BE + HGD | Case series  Multicentre  Retrospective  *Countries:* US  *Length of follow-up:* Mean: 41 months ± 9 months | *Number of patients:* 15  *Gender:*  Male: 13  Female: 2  *Age:*  Mean 63 yrs  Range: 35 to 76 yrs  *Prior treatments:* none reported  *Length of Barrett’s:* not reported  *Inclusion criteria*: none notable  *Exclusion criteria:* none notable | Esophagectomy  *Type of surgery:*  THE (9 patients)  TTE with chest anastomosis (3 patients)  TTE with cervical anastomosis (2 patients)  Modified Ivor Lewis esophagectomy (1 patient)  *Operative time:* not reported  *Co-interventions:* none reported | *Outcomes:*  Progression to cancer  Survival  Length of stay (LOS)  *Adverse events* | | *Outcomes:*  Progression to cancer at 41 months (mean): 0/15 patients (0%)  Survival at 41 months (mean): 15/15 patients (100%)  LOS:  Mean: 18.5 ± 3.0 days  Median: 16 days  *Adverse events*:  Anastomotic leaks 11/15 patients (73%)  Pulmonary complications: 4/15 patients (27%)  Cardiovascular complications: 3/15 patients (20%)  Infection: 5/15 patients (33%)  Other complications 5/15 patients (33%) | 4 |
| Nguyen NT, et al. (2000)[109] | BE + HGD | Case series  Single centre  Countries: US  *Length of follow-up:*  Mean: 12.6 months | *Number of patients:* 12  *Gender:*  Male:7  Female: 5  *Age:*  Mean 64 yrs  Range 40-78 yrs  *Prior treatments:*  Abdominal surgery (5 patients)  *Length of Barrett’s:* not reported  *Inclusion criteria:*  Karnofsky score >60  >50% predicted force expiratory volume in 1 second  Vital capacity score >60  *Exclusion criteria:* none notable | Esophagectomy  *Type of surgery:*  MIE with cervical anastomosis  *Operative time:*  Mean: 7.8 hours ± 2.1 hours  *Co-interventions:* none reported | *Outcomes:*  Survival  Length of stay (LOS)  -in ICU  -in hospital  *Adverse events:* | | *Outcomes:*  Survival at 12.6 months (mean): 12/12 patients (100%)  LOS:  -in ICU:  Mean: 2.6 days  Range: 1 to 8 days)  -in hospital:  Mean: 8.3 days  Range: 4 to 21 days  *Adverse events:*  Small bowel perforation 1/12 patient (8%)  Respiratory insufficiency: 2/12 patients (17%)  Delayed gastric emptying requiring pyloroplasty: 3/12 patients (25%)  J-tube infection: 1/12 patients (8%) | 4 |
| Romagnoli R (2003)[110] | BE + LGD (3 patients)  BE + HGD (24 patients)  BE + HGD + LGD (6 patients) | Case series  Single centre  Retrospective  *Countries:* Belgium  *Length of follow-up:* 120 months | *Number of patients:*33  (prompt ER: 20 patients; expectant ER: 13 patients)  *Gender:*  Male: 28  Female: 5  *Age:*  Range: 41 to 79 years  Prompt Esophagectomy Group  Number of patients: 20  Gender: not reported  Age: not reported  Expectant Esophagectomy Group  Number of patients: 13  Gender: not reported  Age: not reported  *Prior treatments:*  ARS (3 patients)  *Length of Barrett’s:* not reported  *Inclusion criteria:* none notable  *Exlusion criteria:* none notable | Esophagectomy  *Type of surgery:*  TTE with neck or thoracic anastomosis  THE  MIE (thoracoscopy)  *Operative time:* not reported  Prompt Esophagectomy Group  Esophagectomy after HGD detected in 1 or 2 endoscopies  Expectant Esophagectomy Group  Esophagectomy after 3 to 5 subsequent endoscopies positive for HGD or detection of EAC  *Co-interventions:* none reported | *Outcomes:*  Survival:  -Prompt Esophagectomy Group  -Expectant Esophagectomy Group  Neoplastic recurrence:  -Prompt Esophagectomy Group  -Expectant Esophagectomy Group  *Adverse events:* none reported | | *Outcomes:*  Survival at 120 months:  -Prompt Esophagectomy Group: 100%  -Expectant Esophagectomy Group: 52.5%  (p=0.0094)  Neoplastic recurrence at 120 months:  -Prompt Esophagectomy Group: 1/20 patients (5%)  -Expectant Esophagectomy Group: 4/13 patients (31%)  p=0.094 | 4 |
| Sujendran V, et al. (2005)[111] | BE + HGD | Case series  Single centre  Prospective  *Countries:* US  *Length of follow-up:* Median: 32 months Range: 3 to 68 months | *Number of patients:* 17  *Gender:*  Male: 15  Female: 2  *Age:*  Mean: 62 yrs  Range: 53 to 74 yrs  *Prior treatments:* none reported  *Length of Barrett’s:* not reported  *Inclusion criteria:* none notable  *Exclusion criteria*: none notable | Esophagectomy  *Type of surgery:*  THE (16 patients)  Ivor Lewis (1 patient)  *Operative time:* not reported  *Co-interventions:* none reported | *Outcomes:*  Disease free survival  Length of stay (LOS):  -in ICU  -in hospital  *Adverse event* | | *Outcomes:*  Disease free survival:  - at 12 months: 17 patients (100%)  - at 24 months: 94%  - at 36 months:82%  - at 43 months:70%  *LOS*:  -in ICU:  1 day: 16 patients (94%)  7 days: 1 patient secondary to acute lung injury (6%)  -in hospital  Median: 11 days  Range: 9-26 days  *Adverse events:*  Lung injury, acute, requiring 7 days in ICU: 1/17 patient (6%)  Pneumonia: 3/17 patients (18%)  Anastomotic leak: 3/17 patients (18%) | 4 |
| Thomson BNJ & Cade RJ (2003)[112] | BE + HGD | Cohort study  Single centre  Prospective  Esophagectomy vs Surveillance  *Countries:* Australia  *Length of follow-up:*  Esophagectomy Group  Mean: 17.3 months  Range 8 to 31 months  Surveillance Group  Mean: 44 months  Range 7 to 74 months | *Number of patients: 12*  (Esophagectomy Group: 7 patients; Surveillance Group: 5 patients)  Esophagectomy Group  *Gender:* not reported  *Age:*  Mean: 59 yrs  Range: 50 to 74 yrs  Surveillance Group  *Gender:* not reported  *Age:*  Mean: 56.4 yrs  Range: 46 to 72 yrs  *Prior treatments:* none reported  *Length of Barrett’s:* not reported  *Inclusion criteria:* none notable  *Exclusion criteria:* none notable | Esophagectomy vs. Surveillance  Esophagectomy Group  No details reported  Surveillance Group  No details reported  *Co-interventions:* not reported | *Outcomes:*  Survival  *Adverse events* | *Outcomes:*  Survival:  -Esophagectomy Group, at 17.3 months (mean): 7/7 patients (100%)  -Surveillance Group, at 44 months (mean): 5/5 patients (100%)  *Adverse events:*  Esophagectomy Group  Pulmonary embolus: 1/7 patients (14%)  MRSA wound infection: 1/7 patients (14%)  Respiratory complications; cardiac complications; recurrent laryngeal nerve palsy, radiological leak; DVT: 0/7 patients (0%)  Surveillance Group  None reported | | 4 |

***Note:*** APC (argon plasma coagulation), ARS (anti-reflux surgery), BE (Barrett’s esophagus), CR (complete response), DVT (deep vein thrombosis), EAC (esophageal adenocarcinoma), EMR (endoscopic mucosal resection), HGD (high grade dysplasia), HpD (hematoporphyrin derivative), IV (intravenous), LGD (low grade dysplasia), OM (omeprazole), PDT (photodynamic therapy), PPI (proton pump inhibitor)
