# Supplementary material for: Endoscopic treatments for Barrett's esophagus: a systematic review of safety and effectiveness compared to esophagectomy
Source: BMC Gastroenterol. 2010 Sep 27;10:111. doi: 10.1186/1471-230X-10-111 (PMC2955687; doi:10.1186/1471-230X-10-111)
Supplement: Additional file 9 — Studies of adverse events (endoscopic treatments). Adverse events reported for individual studies of endoscopic alternatives are presented in Additional file 9. [file 1471-230X-10-111-S9.DOC]

| Additional file 9 - Studies of adverse events (endoscopic treatments) | | | | | | | |
| --- | --- | --- | --- | --- | --- | --- | --- |
| **Study** | **No. of**  **patients who received treatment** | **Dysphagia** | **Photosen.*** | **Stricture** | **Perfor.*** | **Odynoph.*** | **Bleed.*** |
| **PDT (ALA 15 mg/kg )** | | | | | | | |
| *Comparative studies* - none | | | | | | | |
| *Non-comparative studies* | | | | | | | |
| Ortner MA, et al. (2002)[26] | 14 | 15.4%  (2/13) | 23.1%  (3/13) | 0%  (0/13) | 0%  (0/13) | 0%  (0/13) | 0%  (0/13) |
| Ortner M, et al. (1997)[27] | 9 | ‘Occasion-ally’ | 0%  (0/9) | 0%  (0/9) | 0%  (0/9) | 0%  (0/9) | 0%  (0/9) |
| Pooled total | 23 | 15.4%  (2/13) | 13.6% (3/22)  0-23.1% | 0%  (0/22) | 0%  (0/22) | 0%  (0/22) | 0%  (0/22) |
| **PDT (ALA 30 mg/kg )** | | | | | | | |
| *Comparative studies* | | | | | | | |
| Kelty CJ, et al. (2004)[14] | 35 | 0%  (0/34) | 14.7%  (5/34) | 0%  (0/34) | 0%  (0/34) | 2.9%  (1/34) | 0%  (0/34) |
| *Non-comparative studies* | | | | | | | |
| Ackroyd R, et al. (2003)[28] | 40 | 0%  (0/40) | 2.5%  (1/40) | 0%  (0/40) | 0%  (0/40) | 0%  (0/40) | 0%  (0/40) |
| Ackroyd R, et al. (1997)[29] | 5 | Not  reported | Not  reported | Not  reported | Not reported | Not  reported | Not  reported |
| Ackroyd R, et al. (1999)[28] | 7 | Not  reported | Not  reported | Not  reported | Not reported | Not  reported | Not  reported |
| Mackenzie G, et al. (2005)[30] | 16 | 0%  (0/16) | 0%  (0/16) | 0%  (0/16) | 0%  (0/16) | 0%  (0/16) | 0%  (0/16) |
| Mackenzie G, et al. (2005)[22] | 16 | 0%  (0/16) | 0%  (0/16) | 0%  (0/16) | 0%  (0/16) | 0%  (0/16) | GI bleed:1 requiring transfusion  (*see note 3 below)* |
| Pooled total | 119 | 0%  (0/106) | 5.7%  (6/106)  0-14.7% | 0%  (0/106) | 0%  (0/106) | 0.9%  (1/106)  0-2.9% | 0%  (0/90) |
| **PDT (ALA 40 mg/kg )** | | | | | | | |
| *Comparative studies*-none | | | | | | | |
| *Non-comparative studies* | | | | | | | |
| Peters F, et al. (2005)[32] | 20 | 0%  (0/20) | 0%  (0/20) | 0%  (0/20) | 0%  (0/20) | 0%  (0/20) | 0%  (0/20) |
| van Hillegersberg R, et al. (2003)[33] | 2 | 0%  (0/2) | 0%  (0/2) | 0%  (0/2) | 0%  (0/2) | 0%  (0/2) | 0%  (0/2) |
| Pooled total | 22 | 0%  (0/22) | 0%  (0/22) | 0%  (0/22) | 0%  (0/22) | 0%  (0/22) | 0%  (0/22) |
| **PDT (ALA 60 mg/kg )** | | | | | | | |
| *Comparative studies* | | | | | | | |
| Behrens A, et al. (2005)[25] | 27 | 0%  (0/27) | 0%  (0/27) | 0%  (0/27) | 0%  (0/27) | 0%  (0/27) | 0%  (0/27) |
| Hage M, et al. (2004)[13] | 26 | 0%  (0/26) | 0%  (0/26) | 0%  (0/26) | 0%  (0/26) | 92.3%  (24/26) | 0%  (0/26) |
| Zoepf T, et al. (2003)[16] | 10 | 40.0%  (4/10) | 0%  (0/10) | 0%  (0/10) | 0%  (0/10) | 0%  (0/10) | 0%  (0/10) |
| *Non-comparative studies* | | | | | | | |
| Barr H, et al. (1996)[34] | 5 | 0%  (0/5) | 0%  (0/5) | 0%  (0/5) | 0%  (0/5) | 0%  (0/5) | 0%  (0/5) |
| Gossner L, et al. (1998)[35] | 10 | Not  reported | Not  reported | Not  reported | Not reported | Not  reported | Not  reported |
| Gossner L, et al. (1999)[36] | 2 | 0%  (0/2) | 0%  (0/2) | 0%  (0/2) | 0%  (0/2) | 0%  (0/2) | 0%  (0/2) |
| Kashtan H, et al. (2002)[37] | 8 | 0%  (0/8) | 75.0%  (6/8) | 0%  (0/8) | 0%  (0/8) | 0%  (0/8) | 0%  (0/8) |
| Mackenzie G, et al. (2005)[22] | 33 | 0%  (0/33) | 0%  (0/33) | 0%  (0/33) | 0%  (0/33) | 0%  (0/33) | GI bleed:1 requiring transfusion  *(see note 3 below)* |
| Mackenzie GD, et al. (2008)[38] | 16 | 0%  (0/16) | 0%  (0/16) | 6.3%  (1/16) | 0%  (0/16) | 0%  (0/16) | 0%  (0/16) |
| Macrae FA, et al. (2004)[39] | 8 | 0%  (0/8) | ‘Common’ | 12.5%  (1/8) | 0%  (0/8) | 0%  (0/8) | 0%  (0/8) |
| Mellidez JC, et al. (2005)[40] | 13 | 0%  (0/13) | 0%  (0/13) | 0%  (0/13) | 0%  (0/13) | 0%  (0/13) | 7.7%  (GI bleed 1/13) |
| Pooled total | 158 | 2.7% (4/148)  0-40.0% | 4.3% (6/140)  0-75.0% | 1.4%  (2/148)  0-12.5% | 0%  (0/148) | 16.2%  (24/148)  0-92.3% | 0.9%  (1/115)  0-7.7% |
| **PDT (HpD 1.5 mg/kg )** | | | | | | | |
| *Comparative studies* - none | | | | | | | |
| *Non-comparative studies* | | | | | | | |
| Laukka MA, et al. (1995)[41] | 5 | 0%  (0/5) | 40.0%  (2/5) | 0%  (0/5) | 0%  (0/5) | 0%  (0/5) | 0%  (0/5) |
| Wang KK, et al. (1997)[42] | 55 | 0%  (0/54) | ‘Common’ | 0%  (0/54) | 0%  (0/54) | ‘Common’ | 0%  (0/54) |
| Wang KK, et al. (1999)[43] | 50 | Not  reported | Not  reported | Not  reported | Not reported | Not  reported | Not  reported |
| Pooled total | 110 | 0%  (0/59) | 40.0%  (2/5) | 0%  (0/59) | 0%  (0/59) | 0%  (0/5) | 0%  (0/59) |
| **PDT (mTHPC 0.15 mg/kg )** | | | | | | | |
| *Comparative studies* - none | | | | | | | |
| *Non-comparative studies* | | | | | | | |
| Javaid B, et al. (2002)[44] | 6 | Not  reported | Not  reported | Not  reported | Not reported | Not  reported | Not  reported |
| Lovat LB, et al. (2005)[45] | 7 | Not  reported | Not  reported | Not  reported | Not reported | Not  reported | Not  reported |
| Pooled total | 13 | -** | -** | -** | -** | -** | -** |
| **PDT (Porfimer sodium 2mg/kg )** | | | | | | | |
| *Comparative studies* | | | | | | | |
| Ragunath K, et al. (2005)[15] | 13 | 0%  (0/13) | 15.4%  (2/13) | 15.4%  (2/13) | 0%  (0/13) | 0%  (0/13) | 0%  (0/13) |
| *Non-comparative studies* | | | | | | | |
| Attila T, et al. (2005)[46] | 19 | 0%  (0/19) | 0%  (0/19) | 36.8%  (7/19) | 0%  (0/19) | 0%  (0/19) | 0%  (0/19) |
| Bronner M, et al. (2006)[47] | 138 | 0%  (0/138) | 0%  (0/138) | 0%  (0/138) | 0%  (0/138) | 0%  (0/138) | 0%  (0/138) |
| Keeley SB, et al. (2007)[48] | 13 | Not  reported | Not  reported | Not  reported | Not reported | Not  reported | Not  reported |
| Mackenzie GD, et al. (2008)[38] | 16 | 0%  (0/16) | 43.8%  (7/16) | 37.5%  (6/16) | 0%  (0/16) | 0%  (0/16) | 0%  (0/16) |
| Overholt BF, et al. (2007)[49] | 138 | 18.8%  (26/138) | 68.8%  (95/138) | 36.2%  (50/138) | 0%  (0/138) | 0%  (0/138) | 0%  (0/138) |
| Overholt BF, et al. (2003)[50] | 94 | Not  reported | Not  reported | Not  reported | Not reported | Not  reported | Not  reported |
| Overholt BF, et al. (1997)[51] | 11 | 0%  (0/11) | 0%  (0/11) | 0%  (0/11) | 0%  (0/11) | 0%  (0/11) | 0%  (0/11) |
| Weiss AA, et al. (2006)[52] | 13 | Not  reported | Not  reported | Not  reported | Not reported | Not  reported | Not  reported |
| Wolfsen HC, et al. (2004)[53] | 69 | Not  reported | Not  reported | Not  reported | Not reported | Not  reported | Not  reported |
| Yachimski P, et al. (2008)[54] | 59 | 0%  (0/59) | 0%  (0/59) | 13.6%  (8/59) | 0%  (0/59) | 0%  (0/59) | 0%  (0/59) |
| Pooled total | 583 | 6.6%  (26/394)  0-18.8% | 26.4%  (104/394)  0-68.8% | 18.5%  (73/394)  0-37.5% | 0%  (0/394) | 0%  (0/394) | 0%  (0/394) |
| **APC** | | | | | | | |
| *Comparative studies* | | | | | | | |
| Dulai GS, et al. (2005)[17] | 26 | 0%  (0/26) | 0%  (0/26) | 0%  (0/26) | 0%  (0/26) | 0%  (0/26) | 0%  (0/26) |
| Hage M, et al. (2004)[13] | 14 | 0%  (0/14) | 0%  (0/14) | 7.1%  (1/14) | 0%  (0/14) | 85.7%  (12/14) | 0%  (0/14) |
| Kelty CJ, et al. (2004)[14] | 37 | 2.9%  (1/34) | 0%  (0/34) | 0%  (0/34) | 0%  (0/34) | 94.1%  (32/34) | 0%  (0/34) |
| Ragunath K, et al. (2005)[15] | 13 | 0%  (0/13) | 0%  (0/13) | 23.1%  (3/13) | 0%  (0/13) | 7.7%  (1/13) | 0%  (0/13) |
| Sharma P, et al. (2006)[18] | 19 | 10.5%  (2/19) | 0%  (0/19) | 5.3%  (1/19) | 0%  (0/19) | 0%  (0/19) | 0%  (0/19) |
| Thomas T, et al. (2005)[55] | 5 | Not  reported | Not  reported | Not  reported | Not reported | Not  reported | Not  reported |
| Zoepf T, et al. (2003)[16] | 10 | 30.0%  (3/10) | 0%  (0/10) | 0%  (0/10) | 0%  (0/10) | 0%  (0/10) | 0%  (0/10) |
| *Non-comparative studies* | | | | | | | |
| Attwood SE, et al. (2003)[56] | 29 | 0%  (0/29) | 0%  (0/29) | 0%  (0/29) | 3.4%  (1/29) | 0%  (0/29) | 0%  (0/29) |
| Basu KK (2006)[57] | 33 | Not  reported | Not  reported | Not  reported | Not reported | Not  reported | Not  reported |
| Brand B, et al. (2000)[58] | 12 | 0%  (0/12) | 0%  (0/12) | 0%  (0/12) | 0%  (0/12) | 91.7%  (11/12) | 0%  (0/12) |
| Bright T, et al. (2007)[59] | 20 | 0%  (0/20) | 0%  (0/20) | 10.0%  (2/20) | 0%  (0/20) | ‘some’ | 0%  (0/20) |
| Dumoulin FL, et al. (1997)[60] | 2 | 100%  (2/2) | 0%  (0/2) | 0%  (0/2) | 0%  (0/2) | 0%  (0/2) | 0%  (0/2) |
| Familiari L (2003)[61] | 32 | 0%  (0/32) | 0%  (0/32) | 0%  (0/32) | 0%  (0/32) | 0%  (0/32) | 0%  (0/32) |
| Ferraris R, et al. (2007)[62] | 96 | Not  reported | Not  reported | Not  reported | Not reported | Not  reported | Not  reported |
| Formentini A (2007)[63] | 21 | 4.8%  (1/21) | 0%  (0/21) | 4.8%  (1/21) | 0%  (0/21) | 0%  (0/21) | 0%  (0/21) |
| Grade AJ, et al. (1999)[64] | 9 | 0%  (0/9) | 0%  (0/9) | 0%  (0/9) | 0%  (0/9) | 11.1%  (1/9) | 0%  (0/9) |
| Madisch A, et al. (2005)[65] | 73 | 0%  (0/73) | 0%  (0/73) | 4.1%  (3/73) | 0%  (0/73) | 0%  (0/73) | 0%  (0/73) |
| Manner H, et al. (2007)[66] | 104 | 9.6%  (10/104) | 0%  (0/104) | 1.0%  (1/104) | 0%  (0/104) | 0%  (0/104) | 0%  (0/104) |
| Manner H, et al. (2006)[67] | 41 | 0%  (0/41) | 0%  (0/41) | 2.4%  (1/41) | 0%  (0/41) | 0%  (0/41) | 0%  (0/41) |
| Manner H, et al. (2006)[23] | 51 | 0%  (0/51) | 0%  (0/51) | 3.9%  (2/51) | 2.0%  (1/51) | 3.9%  (2/51) | 3.9%  (2/51 requiring transfusion) |
| Pedrazzani C, et al. (2005)[68] | 25 | 2/40 sessions  (*see note 2 below)* | 0/40  sessions  (*see note 2 below)* | 0/40  sessions  (*see note 2 below)* | 0/40  sessions  (*see note 2 below)* | 0/40  sessions  (*see note 2 below)* | 1/40 sessions  (*see note 2 below)* |
| Pereira-Lima JC, et al. (2000)[69] | 33 | 0%  (0/33) | 0%  (0/33) | 9.1%  (3/33) | 0%  (0/33) | 54.5%  (18/33) | 0%  (0/33) |
| Pinotti AC, et al. (2004)[70] | 19 | 21.1%  (4/19) | 0%  (0/19) | 0%  (0/19) | 0%  (0/19) | 21.1%  (4/19) | 0%  (0/19) |
| Tigges H, et al. (2001)[71] | 30 | 6.7%  (2/30) | 0%  (0/30) | 3.3%  (1/30) | 0%  (0/30) | 6.7%  (2/30) | 0%  (0/30) |
| Van Laethem JL, et al. (2001)[72] | 7 | Not  reported | Not  reported | Not  reported | Not reported | Not  reported | Not  reported |
| Van Laethem JL, et al. (1998)[73] | 31 | 6.5%  (2/31) | 0%  (0/31) | 6.5%  (2/31) | 0%  (0/31) | 6.5%  (2/31) | 3.2%  (1/31) |
| Pooled total | 792 | 4.3%  (27/623)  0-100% | 0%  (0/623) | 3.4%  (21/623)  0-23.1% | 0.3%  (2/623)  0-3.4% | 13.6%  (85/623)  0-94.1% | 0.5%  (3/623)  0-3.9% |
| **Cryoablation** | | | | | | | |
| *Comparative studies* - none | | | | | | | |
| *Non-comparative studies* | | | | | | | |
| Dumot JA, et al. (2008)[74] | 20 | Not  reported | Not  reported | Not  reported | Not reported | Not  reported | Not  reported |
| Johnston MH (2005)[75] | 11 | 9.1%  (1/11) | 0%  (0/11) | 0%  (0/11) | 0%  (0/11) | 0%  (0/11) | 0%  (0/11) |
| Pooled total | 31 | 9.1%  (1/11) | 0%  (0/11) | 0%  (0/11) | 0%  (0/11) | 0%  (0/11) | 0%  (0/11) |
| **Combined EMR & PDT** | | | | | | | |
| *Comparative studies* | | | | | | | |
| Behrens A, et al.  (2005)[25] | 3 | 0%  (0/3) | 0%  (0/3) | 0%  (0/3) | 0%  (0/3) | 0%  (0/3) | 0%  (0/3) |
| *Non-comparative studies* | | | | | | | |
| Wolfsen HC, et al.  (2004)[76] | 3 | 0%  (0/3) | 0%  (0/3) | 0%  (0/3) | 0%  (0/3) | 0%  (0/3) | 0%  (0/3) |
| Pooled total | 6 | 0%  (0/6) | 0%  (0/6) | 0%  (0/6) | 0%  (0/6) | 0%  (0/6) | 0%  (0/6) |
| **Thermocoagulation** | | | | | | | |
| *Comparative studies* - none | | | | | | | |
| *Non-comparative studies* | | | | | | | |
| Michopoulos S, et al. (1999)[77] | 13 | 0%  (0/13) | 0%  (0/13) | 0%  (0/13) | 0%  (0/13) | 0%  (0/13) | 0%  (0/13) |
| Pooled total | 13 | 0%  (0/13) | 0%  (0/13) | 0%  (0/13) | 0%  (0/13) | 0%  (0/13) | 0%  (0/13) |
| **EMR** | | | | | | | |
| *Comparative studies* | | | | | | | |
| Behrens A, et al. (2005)[25] | 14 | 0%  (0/14) | 0%  (0/14) | 0%  (0/14) | 0%  (0/14) | 0%  (0/14) | 0%  (0/14) |
| Reed MF, et al. (2005)[20] | 5 | 0%  (0/5) | 0%  (0/5) | 0%  (0/5) | 0%  (0/5) | 0%  (0/5) | 0%  (0/5) |
| *Non-comparative studies* | | | | | | | |
| Giovannini M, et al. (2004)[78] | 12 | 0%  (0/12) | 0%  (0/12) | 0%  (0/12) | 0%  (0/12) | 0%  (0/12) | 25.0%  (3/12) |
| Mino-Kenudson M, et al. (2005)[79] | 3 | Not  reported | Not  reported | Not  reported | Not reported | Not  reported | Not  reported |
| Seewald S, et al. (2003)[80] | 3 | Not  reported | Not  reported | Not  reported | Not reported | Not  reported | Not  reported |
| Tang SJ, et al. (2008)[81] | 1 | 0%  (0/1) | 0%  (0/1) | 0%  (0/1) | 0%  (0/1) | 0%  (0/1) | 0%  (0/1) |
| Pooled total | 38 | 0%  (0/32) | 0%  (0/32) | 0%  (0/32) | 0%  (0/32) | 0%  (0/32) | 9.4%  (3/32)  0-25.0% |
| **Laser ablation** | | | | | | | |
| *Comparative studies* - none | | | | | | | |
| *Non-comparative studies* | | | | | | | |
| Barham CP, et al. (1997)[82] | 16 | 0%  (0/16) | 0%  (0/16) | 0%  (0/16) | 0%  (0/16) | 0%  (0/16) | 0%  (0/16) |
| Bonavina L, et al. (1999)[83] | 18 | 0%  (0/16) | 0%  (0/16) | 12.5%  (2/16) | 0%  (0/16) | 0%  (0/16) | 0%  (0/16) |
| Bowers SP, et al. (2003) [84] | 9 | Not  reported | Not  reported | Not  reported | Not reported | Not  reported | Not  reported |
| Ertan A, et al. (1995)[85] | 1 | Not  reported | Not  reported | Not  reported | Not reported | Not  reported | Not  reported |
| Fisher RS, et al. (2003)[24] | 21 | 0%  (0/21) | 0%  (0/21) | 4.8%  (1/21) | 4.8%  (1/21) | 0%  (0/21) | 4.8%  (1/21) required transfusion |
| Norberto L, et al. (2004)[86] | 15 | 0%  (0/15) | 0%  (0/15) | 0%  (0/15) | 0%  (0/15) | 0%  (0/15) | 0%  (0/15) |
| Salo JA, et al. (1998)[87] | 11 | Not  reported | Not  reported | Not  reported | Not reported | Not  reported | Not  reported |
| Pooled total | 91 | 0%  (0/68) | 0%  (0/68) | 4.4%  (3/68)  0-12.5% | 1.5%  (1/68)  0-4.8% | 0%  (0/68) | 1.5%  (1/68)  0-4.8% |
| **MPEC** | | | | | | | |
| *Comparative studies* | | | | | | | |
| Dulai GS, et al. (2005)[17] | 26 | 0%  (0/26) | 0%  (0/26) | 0%  (0/26) | 0%  (0/26) | 0%  (0/26) | 0%  (0/26) |
| Sharma P, et al. (2006)[18] | 16 | 31.3%  (5/16) | 0%  (0/16) | 0%  (0/16) | 0%  (0/16) | 0%  (0/16) | 0%  (0/16) |
| *Non-comparative studies* | | | | | | | |
| Faigel DO, et al. (2002)[88] | 25 | Not  reported | Not  reported | Not  reported | Not reported | Not  reported | Not  reported |
| Kovacs BJ, et al. (1999)[89] | 27 | 40.7%  (11/27) | 0%  (0/27) | 3.7%  (1/27) | 0%  (0/27) | 40.7%  (11/27) | 0%  (0/27) |
| Montes CG, et al. (1999)[90] | 14 | 7.1%  (1/14) | 0%  (0/14) | 0%  (0/14) | 0%  (0/14) | 14.3%  (2/14) | 0%  (0/14) |
| Sampliner RE, et al. (1996)[91] | 10 | 10.0%  (1/10) | 0%  (0/10) | 0%  (0/10) | 0%  (0/10) | 20.0%  (2/10) | 10.0%  (1/10) |
| Sampliner RE (1999)[92] | 11 | 63.6%  (7/11) | 0%  (0/11) | 0%  (0/11) | 0%  (0/11) | 0%  (0/11) | 0%  (0/11) |
| Pooled total | 129 | 24.0%  (25/104)  0-63.6% | 0%  (0/104) | 1.0%  (1/104)  0-3.7% | 0%  (0/104) | 14.4% (15/104)  0-40.7% | 1.0%  (1/104)  0-10.0% |
| **RFA** | | | | | | | |
| *Comparative studies* | | | | | | | |
| Bumgarner JM, et al. (2008)[93] | 103 | Not  reported | Not  reported | Not  reported | Not reported | Not  reported | Not  reported |
| Shaheen NJ, et al. (2009)[19] | 84 | 0%  (0/84) | 0%  (0/84) | 6.0%  (5/84) | 0%  (0/84) | 0%  (0/84) | 1.2%  (1/84)  (*see note 4 below*) |
| *Non-comparative studies* | | | | | | | |
| Eldaif SM, et al. (2009)[94] | 27 | 0%  (0/27) | 0%  (0/27) | 0%  (0/27) | 0%  (0/27) | 0%  (0/27) | 0%  (0/27) |
| Fleischer DE, et al. (2008)[95] | 70  (after 1.5 sessions /patient) | 0%  (0/70) | 0%  (0/70) | 0%  (0/70) | 0%  (0/70) | 0%  (0/70) | 1.4%  (1/70) |
| Ganz RA, et al. (2008)[96] | 142 | 0%  (0/142) | 0%  (0/142) | 0.7%  (1/142) | 0%  (0/142) | 0%  (0/142) | 0%  (0/142) |
| Hernandez JC, et al. (2008)[97] | 10 | 0%  (0/10) | 0%  (0/10) | 0%  (0/10) | 0%  (0/10) | 0%  (0/10) | 0%  (0/10) |
| Hubbard N & Velanovich V (2007)[98] | 7 | Not  reported | Not  reported | Not  reported | Not reported | Not  reported | Not  reported |
| Pouw RE, et al. (2008)[99] | 44 | 9.1%  (4/44) | 0%  (0/44) | 0%  (0/44) | 0%  (0/44) | 0%  (0/44) | 0%  (0/44) |
| Roorda AK, et al. (2007)[99] | 13 | 23.1%  (3/13) | 0%  (0/13) | 0%  (0/13) | 0%  (0/13) | 23.1%  (3/13) | 0%  (0/13) |
| Sharma VK, et al. (2007)[101] | 32 | 0%  (0/32) | 0%  (0/32) | 0%  (0/32) | 0%  (0/32) | 0%  (0/32) | 0%  (0/32) |
| Smith CD, et al. (2007)[102] | 5 | Not  reported | Not  reported | Not  reported | Not reported | Not  reported | Not  reported |
| Sharma VK, et al. (2009)[103] | 63 | 0%  (0/63) | 0%  (0/63) | 1.6%  (1/63) | 0%  (0/63) | 0%  (0/63) | 1.6%  (1/63) |
| Vassiliou MC, et al. (2009)[104] | 25 | 0/59 ablations  (*see note 5 below)* | 0/59  ablations  (*see note 5 below)* | 2/59 ablations  (*see note 5 below)* | 0/59 ablations  (*see note 5 below)* | 0/59 ablations  (*see note 5 below)* | 1/59 ablations  (*see note 5 below)* |
| Velanovich V (2009)[105] | 66 | 0%  (0/66) | 0%  (0/66) | 6.1%  (4/66) | 0%  (0/66) | 0%  (0/66) | 0%  (0/66) |
| Gondrie JJ, et al. (2008)[106] | 11 | 0%  (0/11) | 0%  (0/11) | 0%  (0/11) | 0%  (0/11) | 0%  (0/11) | 0%  (0/11) |
| Gondrie JJ, et al. (2008)[21] | 12 | 8.3%  (1/12) | 0%  (0/12) | 0%  (0/12) | 0%  (0/12) | 0%  (0/12) | 0%  (0/12) |
| Pooled total | 714 | 1.4%  (8/574)  0-23.1% | 0%  (0/574) | 1.9%  (11/574)  0-6.1% | 0%  (0/574) | 0.5%  (3/574)  0-23.1% | 0.5%  (3/574)  0-1.6% |

***Notes:*** (1) ALA (aminolevulinic acid), APC (argon plasma coagulation), EMR (endoscopic mucosal resection), GI (gastrointestinal), HpD (hematoporphyrin derivative), MPEC (multipolar electrocoagulation), mTHPC (meta-tetrahydroxyphenylchlorin), PDT (photodynamic therapy), RFA (radiofrequency ablation) (2) As these numbers are rates per number of sessions, they have not been included in the pooled totals. (3) As a single case of a gastrointestinal bleed reported in the MacKenzie G et al (2005) study cannot be definitely attributed to the 30 mg/kg ALA treatment or the 60 mg/kg ALA treatment, it has not been included in the pooled totals. (4) Possibly or probably related to RFA (5) As these numbers are rates per number of ablations, they have not been included in the pooled totals. (6) *Photosen. (photosensitivity), Perfor. (perforation), Odynoph. (odynophagia), Bleed. (bleeding), ** - (not available)
