# Supplementary material for: Endoscopic treatments for Barrett's esophagus: a systematic review of safety and effectiveness compared to esophagectomy
Source: BMC Gastroenterol. 2010 Sep 27;10:111. doi: 10.1186/1471-230X-10-111 (PMC2955687; doi:10.1186/1471-230X-10-111)
Supplement: Additional file 10 — Studies of complete eradication of Barrett's esophagus (endoscopic treatments). Values reported for the complete eradication of BE with endoscopic treatments are presented in Additional file 10. [file 1471-230X-10-111-S10.DOC]

| Additional file 10 - Studies of complete eradication of Barrett’s esophagus (endoscopic treatments) | | |
| --- | --- | --- |
| **Study** | **No. of patients who**  **received treatment** | **Complete eradication of BE**  **(up to 3 months post-treatment)** |
| **PDT (ALA 15mg/kg)** |  |  |
| *Comparative studies* - none |  |  |
| *Non-comparative studies* |  |  |
| Ortner M, et al. (1997)[27] | 9 | 44.4% (4/9) |
| Ortner MA, et al. (2002)[26] | 14 | 21.4 (3/14) |
| Pooled total | 23 | 30.4% (7/23)  21.4% - 44.4% |
| **PDT (ALA 30mg/kg)** |  |  |
| *Comparative studies* |  |  |
| Kelty CJ, et al. (2004)[14] | 35 | 50.0% (17/34) |
| *Non-comparative studies* |  |  |
| Ackroyd R, et al. (2003)[28] | 40 | Not reported |
| Ackroyd R, et al. (1999)[30] | 7 | 14.3% (1/7) |
| Ackroyd R, et al. (1997)[29] | 5 | Not reported |
| Mackenzie G, et al. (2005)[31] | 16 | Not reported |
| Mackenzie G, et al. (2005)[22] | 16 | Not reported |
| Pooled total | 119 | 43.9% (18/41)  14.3% - 50.0% |
| **PDT (ALA 40mg/kg)** |  |  |
| *Comparative studies* - none |  |  |
| *Non-comparative studies* |  |  |
| Peters F, et al. (2005)[32] | 20 | Not reported |
| Van Hillegersberg R, et al. (2003)[33] | 2 | 0% |
| Pooled total | 22 | 0% (0/2) |
| **PDT (ALA 60mg/kg)** |  |  |
| *Comparative studies* |  |  |
| Behrens A, et al. (2005)[25] | 27 | Not reported |
| Hage M, et al. (2004)[13] | 26 | 19.2% (5/26)  (PDT20+100 group: 4/13  PDT100 group: 1/13) |
| Zoepf T, et al. (2003)[16] | 10 | Not reported |
| *Non-comparative studies* |  |  |
| Barr H, et al. (1996)[34] | 5 | Not reported |
| Gossner L, et al. (1998)[35] | 10 | Not reported |
| Gossner L, et al. (1999)[36] | 2 | Not reported |
| Kashtan H, et al. (2002)[38] | 8 | Not reported |
| Mackenzie G, et al. (2005)[22] | 33 | Not reported |
| Mackenzie GD, et al. (2008)[38] | 16 | Not reported |
| Macrae FA, et al. (2004)[39] | 8 | Not reported |
| Mellidez JC, et al. (2005)[40] | 13 | Not reported |
| Pooled total | 158 | 19.2% (5/26) |
| **PDT (HpD 1.5mg/kg)** |  |  |
| *Comparative studies* - none |  |  |
| *Non-comparative studies* |  |  |
| Laukka MA, et al. (1995)[41] | 5 | 0% |
| Wang KK, et al. (1997)[42] | 55 | Not reported |
| Wang KK, et al. (1999)[43] | 50 | Not reported |
| Pooled total | 110 | 0% (0/5) |
| **PDT (mTHPC 0.15mg/kg)** |  |  |
| *Comparative studies* - none |  |  |
| *Non-comparative studies* |  |  |
| Javaid B, et al. (2002)[44] | 6 | 16.7% (1/6) |
| Lovat LB, et al. (2005)[45] | 7 | Not reported |
| Pooled total | 13 | 16.7% (1/6) |
| **PDT (Porfimer sodium 2mg/kg)** |  |  |
| *Comparative studies* |  |  |
| Ragunath K, et al. (2005)[15] | 13 | Not reported |
| *Non-comparative studies* |  |  |
| Attila T, et al. (2005)[46] | 19 | 26.3% (5/19) |
| Bronner M, et al. (2006)[47] | 138 | Not reported |
| Keeley SB, et al. (2007)[48] | 13 | Not reported |
| Mackenzie GD, et al. (2008)[38] | 16 | Not reported |
| Overholt BF, et al. (2007)[49] | 138 | Not reported |
| Overholt BF, et al. (2003)[50] | 94 | 56.4% (53/94) |
| Overholt BF, et al. (1997)[51] | 11 | Not reported |
| Weiss AA, et al. (2006)[52] | 13 | Not reported |
| Wolfsen HC, et al. (2004)[53] | 69 | 52.2% (36/69) |
| Yachimski P, et al. (2008)[54] | 59 | Not reported |
| Pooled total | 583 | 51.6% (94/182)  26.3% - 56.4% |
| **APC** |  |  |
| *Comparative studies* |  |  |
| Dulai GS, et al. (2005)[17] | 26 | 80.8% (21/26) |
| Hage M, et al. (2004)[13] | 14 | 35.7% (5/14) |
| Kelty CJ, et al. (2004)[14] | 37 | 97.1% (33/34) |
| Ragunath K, et al. (2005)[15] | 13 | Not reported |
| Sharma P, et al. (2006)[18] | 19 | Not reported |
| Thomas T, et al. (2005)[55] | 5 | Not reported |
| Zoepf T, et al. (2003)[16] | 10 | Not reported |
| *Non-comparative studies* |  |  |
| Attwood SE, et al. (2003)[56] | 29 | 75.9% (22/29) |
| Basu KK (2006)[57] | 33 | 84.8% (28/33) |
| Brand B, et al. (2000)[58] | 12 | 91.7% (11/12) |
| Bright T, et al. (2007)[59] | 20 | 60.0% (12/20) |
| Dumoulin FL, et al. (1997)[60] | 2 | 0% (0/2) |
| Familiari L (2003)[61] | 32 | 100% (32/32) |
| Ferraris R, et al. (2007)[62] | 96 | Not reported |
| Formentini A (2007)[63] | 21 | 100% (17/17) |
| Grade AJ, et al. (1999)[64] | 9 | 77.8% (7/9) |
| Madisch A, et al. (2005)[65] | 73 | 98.6% (69/70) |
| Manner H, et al. (2007)[66] | 104 | Not reported |
| Manner H, et al. (2006)[66] | 41 | Not reported |
| Manner H, et al. (2006)[23] | 51 | Not reported |
| Pedrazzani C, et al. (2005)[68] | 25 | 96.0% (24/25) |
| Pereira-Lima JC, et al. (2000)[69] | 33 | 97.0% (32/33) |
| Pinotti AC, et al. (2004)[70] | 19 | 94.7% (18/19) |
| Tigges H, et al. (2001)[71] | 30 | 100% (22/22) |
| Van Laethem JL, et al. (2001)[72] | 7 | 57.1% (4/7) |
| Van Laethem JL, et al. (1998)[73] | 31 | 48.4% (15/31) |
| Pooled total | 792 | 85.5% (372/435)  0 – 100% |
| **Cryoablation** |  |  |
| *Comparative studies* - none |  |  |
| *Non-comparative studies* |  |  |
| Dumot JA, et al. (2008)[74] | 20 | Not reported |
| Johnston MH (2005)[75] | 11 | 81.8% (9/11) |
| Pooled total | 31 | 81.8% (9/11) |
| **Combined EMR & PDT** |  |  |
| *Comparative studies* |  |  |
| Behrens A, et al. (2005)[25] | 3 | Not reported |
| *Non-comparative studies* |  |  |
| Wolfsen HC, et al. (2004)[76] | 3 | Not reported |
| Pooled total | 6 | -** |
| **Thermocoagulation** |  |  |
| *Comparative studies* - none |  |  |
| *Non-comparative studies* |  |  |
| Michopoulos S, et al. (1999)[77] | 13 | 100% (13/13) |
| Pooled total | 13 | 100% (13/13) |
| **EMR** |  |  |
| *Comparative studies* |  |  |
| Behrens A, et al. (2005)[25] | 14 | Not reported |
| Reed MF, et al. (2005)[20] | 5 | Not reported |
| *Non-comparative studies* |  |  |
| Giovannini M, et al. (2004) [78] | 12 | Not reported |
| Mino-Kenudson M, et al. (2005)[79] | 3 | Not reported |
| Seewald S, et al. (2003)[80] | 3 | Not reported |
| Tang SJ, et al. (2008)[81] | 1 | 100% (1/1) |
| Pooled total | 38 | 100% (1/1) |
| **Laser ablation** |  |  |
| *Comparative studies* - none |  |  |
| *Non-comparative studies* |  |  |
| Barham CP, et al. (1997)[82] | 16 | 81.3% (13/16) |
| Bonavina L, et al. (1999)[83] | 18 | 61.1% (11/18) |
| Bowers SP, et al. (2003)[84] | 9 | 22.2% (2/9) |
| Ertan A, et al. (1995)[85] | 1 | Not reported |
| Fisher RS, et al. (2003)[24] | 21 | 100% (21/21) |
| Norberto L, et al. (2004)[86] | 15 | Not reported |
| Salo JA, et al. (1998)[87] | 11 | 100% (11/11) |
| Pooled total | 91 | 77.3% (58/75)  22.2% - 100% |
| **MPEC** |  |  |
| *Comparative studies* |  |  |
| Dulai GS, et al. (2005)[17] | 26 | 88.5% (23/26) |
| Sharma P, et al. (2006)[18] | 16 | Not reported |
| *Non-comparative studies* |  |  |
| Faigel DO, et al. (2002)[88] | 25 | Not reported |
| Kovacs BJ, et al. (1999)[89] | 27 | Not reported |
| Montes CG, et al. (1999)[90] | 14 | Not reported |
| Sampliner RE, et al. (1996)[91] | 10 | Not reported |
| Sampliner RE (1999)[92] | 11 | Not reported |
| Pooled total | 129 | 88.5% (23/26) |
| **RFA** |  |  |
| *Comparative studies* |  |  |
| Bumgarner JM, et al. (2008)[93] | 103 | Not reported |
| Shaheen NJ, et al. (2009)[19] | 84 | Not reported |
| *Non-comparative studies* |  |  |
| Eldaif SM, et al. (2009)[94] | 27 | 92.6% (25/27) |
| Fleischer DE, et al. (2008)[95] | 70 | Not reported |
| Ganz RA, et al. (2008)[96] | 142 | Not reported |
| Hernandez JC, et al. (2008)[97] | 10 | Not reported |
| Hubbard N & Velanovich V (2007)[98] | 7 | 85.7% (6/7) |
| Pouw RE, et al. (2008)[99] | 44 | 97.7% (43/44) |
| Roorda AK, et al. (2007)[100] | 13 | Not reported |
| Sharma VK, et al. (2007)[101] | 32 | 21.9% (7/32) |
| Smith CD, et al. (2007)[102] | 5 | 9/10 ablation regions  (see note 2 below) |
| Sharma VK, et al. (2009)[103] | 63 | Not reported |
| Vassiliou MC, et al. (2009)[104] | 25 | Not reported |
| Velanovich V (2009)[105] | 66 | 59.2% (29/49) |
| Gondrie JJ, et al. (2008)[106] | 11 | Not reported |
| Gondrie JJ, et al. (2008)[12106] | 12 | 66.7% (8/12) |
| Pooled total | 714 | 69.0% (118/171)  21.9% – 97.7% |

***Notes:*** (1) ALA (aminolevulinic acid), APC (argon plasma coagulation), BE (Barrett’s esophagus), EMR (endoscopic mucosal resection), HpD (hematoporphyrin derivative), MPEC (multipolar electrocoagulation), mTHPC (meta-tetrahydroxyphenylchlorin), PDT (photodynamic therapy), RFA (radiofrequency ablation), **- (not available) (2) As this number is a rate per number of ablation regions, it has not been included in the pooled total.
