# Supplementary material for: Endoscopic treatments for Barrett's esophagus: a systematic review of safety and effectiveness compared to esophagectomy
Source: BMC Gastroenterol. 2010 Sep 27;10:111. doi: 10.1186/1471-230X-10-111 (PMC2955687; doi:10.1186/1471-230X-10-111)
Supplement: Additional file 11 — Studies of complete eradication of high grade dysplasia (endoscopic treatments). Values reported for the complete eradication of HGD with endoscopic treatments are presented in Additional file 11. [file 1471-230X-10-111-S11.DOC]

| **Additional file 11 - Studies of complete eradication of high grade dysplasia (endoscopic treatments)** | | | |
| --- | --- | --- | --- |
| **Study** | | **No. of patients with HGD who**  **received treatment** | **Complete eradication of HGD**  **(up to 3 months post-treatment)** |
| **PDT (ALA 15mg/kg)** | |  |  |
| *Comparative studies* - none | |  |  |
| *Non-comparative studies* – none for HGD | |  |  |
| **PDT (ALA 30mg/kg)** | |  |  |
| *Comparative studies* – none for HGD | |  |  |
| *Non-comparative studies* | |  |  |
| Ackroyd R, et al. (1997)[29] | | 4 | Not reported |
| Ackroyd R, et al. (1999)[30] | | 4 | 100% (4/4) |
| Mackenzie G, et al. (2005)[31] | | 16 | Not reported |
| Mackenzie G, et al. (2005)[22] | | 16 | Not reported |
| Pooled total | | 40 | 100% (4/4) |
| **PDT (ALA 40mg/kg)** | |  |  |
| *Comparative studies* - none | |  |  |
| *Non-comparative studies* | |  |  |
| Peters F, et al. (2005)[32] | | 20 | 75.0% (15/20) |
| Van Hillegersberg R, et al. (2003)[33] | | 2 | 0% |
| Pooled total | | 20 | 68.2% (15/22)  0– 75.0% |
| **PDT (ALA 60mg/kg)** | |  |  |
| *Comparative studies* | |  |  |
| Behrens A, et al. (2005)[25] | | 27 | 96.3% (26/27) |
| Zoepf T, et al. (2003)[16] | | 10 | Not reported |
| *Non-comparative studies* | |  |  |
| Barr H, et al. (1996)[34] | | 5 | Not reported |
| Gossner L, et al. (1998)[35] | | 10 | Not reported |
| Gossner L, et al. (1999)[36] | | 2 | 100% (2/2) |
| Kashtan H, et al. (2002)[37] | | 1 | Not reported |
| Mackenzie G, et al. (2005)[22] | | 33 | Not reported |
| Mackenzie GD, et al. (2008)[38] | | 16 | Not reported |
| Macrae FA, et al. (2004)[39] | | 8 | Not reported |
| Mellidez JC, et al. (2005)[40] | | 13 | Not reported |
| Pooled total | | 125 | 96.6% (28/29)  96.3% - 100% |
| **PDT (HpD 1.5mg/kg)** | |  |  |
| *Comparative studies* - none | |  |  |
| *Non-comparative studies* | |  |  |
| Laukka MA, et al. (1995)[39] | | 1 | 100% (1/1 CR at 2 to 12 months) |
| Wang KK, et al. (1997)[42] | | 55  (Mixed patient population – No. of patients with HGD unknown) | Not reported |
| Wang KK, et al. (1999)[43] | | 11 | Not reported |
| Pooled total | | 67 | -** |
| **PDT (mTHPC 0.15mg/kg)** | |  |  |
| *Comparative studies* - none | |  |  |
| *Non-comparative studies* | |  |  |
| Javaid B, et al. (2002)[44] | | 6 | 66.7% (4/6) |
| Lovat LB, et al. (2005)[45] | | 7 | Not reported |
| Pooled total | | 13 | 66.7% (4/6) |
| **PDT (Porfimer sodium 2mg/kg)** | |  |  |
| *Comparative studies* | |  |  |
| Ragunath K, et al. (2005)[15] | | 2 | Not reported |
| *Non-comparative studies* | |  |  |
| Attila T, et al. (2005)[46] | | 19 | Not reported |
| Bronner M, et al. (2006)[47] | | 138 | Not reported |
| Keeley SB, et al. (2007)[48] | | 13 | Not reported |
| Mackenzie GD, et al. (2008)[38] | | 16 | Not reported |
| Overholt BF, et al. (2007)[49] | | 138 | Not reported |
| Overholt BF, et al. (2003)[50] | | 80 | 77.5% (62/80) |
| Overholt BF, et al. (1997)[51] | | 11 | Not reported |
| Weiss AA, et al. (2006)[52] | | 13 | Not reported |
| Wolfsen HC, et al. (2004)[53] | | 69 | Not reported |
| Yachimski P, et al. (2008)[54] | | 59 | Not reported |
| Pooled total | | 558 | 77.5% (62/80) |
| **APC** | |  |  |
| *Comparative studies* | |  |  |
| Ragunath K, et al. (2005)[15] | | 1 | Not reported |
| Thomas T, et al. (2005)[55] | | 5 | Not reported |
| Zoepf T, et al. (2003)[16] | | 10 | Not reported |
| *Non-comparative studies* | |  |  |
| Attwood SE, et al. (2003)[56] | | 29 | Not reported |
| Pereira-Lima JC, et al. (2000)[69] | | 1 | Not reported |
| Van Laethem JL, et al. (2001)[72] | | 7 | 85.7% (6/7) |
| Pooled total | | 53 | 85.7% (6/7) |
| **Cryoablation** | |  |  |
| *Comparative studies* - none | |  |  |
| *Non-comparative studies* | |  |  |
| Dumot JA, et al. (2008)[74] | | 20 | Not reported |
| Johnston MH (2005)[75] | | 1 | 100% (1/1) |
| Pooled total | | 21 | 100% (1/1) |
| **Combined EMR & PDT** | |  |  |
| *Comparative studies* | |  |  |
| Behrens A, et al. (2005)[25] | | 3 | 66.7% (2/3) |
| *Non-comparative studies* | |  |  |
| Wolfsen HC, et al. (2004)[76] | | 3 | Not reported |
| Pooled total | | 6 | 66.7% (2/3) |
| **Thermocoagulation** | |  |  |
| *Comparative studies* - none | |  |  |
| *Non-comparative studies* - none for HGD | | | |
| **EMR** |  | |  |
| *Comparative studies* |  | |  |
| Behrens A, et al. (2005)[25] | 14 | | 92.9% (13/14) |
| Reed MF, et al. (2005)[20] | 5 | | Not reported |
| *Non-comparative studies* |  | |  |
| Giovannini M, et al. (2004)[78] | 12 | | 100% (12/12) |
| Mino-Kenudson M, et al. (2005)[79] | 3 | | Not reported |
| Seewald S, et al. (2003)[80] | 3 | | Not reported |
| Tang SJ, et al. (2008)[81] | 1 | | 100% (1/1) |
| Pooled total | 38 | | 96.3% (26/27)  92.9% - 100% |
| **Laser ablation** |  | |  |
| *Comparative studies* - none |  | |  |
| *Non-comparative studies* |  | |  |
| Ertan A, et al. (1995)[85] | 1 | | Not reported |
| Fisher RS, et al. (2003)[24] | 3 | | Not reported |
| Norberto L, et al. (2004)[86] | 2 | | Not reported |
| Pooled total | 6 | | -** |
| **MPEC** |  | |  |
| *Comparative studies* – none for HGD |  | |  |
| *Non-comparative studies* – none for HGD | | | |
| **RFA** |  | |  |
| *Comparative studies* |  | |  |
| Bumgarner JM, et al. (2008)[93] | 103 | | Not reported |
| Shaheen NJ, et al. (2009)[19] | 63 | | Not reported |
| *Non-comparative studies* |  | |  |
| Ganz RA, et al. (2008)[96] | 142 | | 90.2% (83/92) |
| Hernandez JC, et al. (2008)[98] | 3 | | Not reported |
| Pouw RE, et al. (2008)[99] | 32 | | Not reported |
| Roorda AK, et al. (2007)[100] | 3 | | Not reported |
| Smith CD, et al. (2007)[102] | 5 | | Not reported |
| Sharma VK, et al. (2009)[103] | 24 | | Not reported |
| Vassiliou MC, et al. (2009)[104] | 15 | | Not reported |
| Velanovich V (2009)[105] | 12 | | Not reported |
| Gondrie JJ, et al. (2008)[106] | 9 | | Not reported |
| Gondrie JJ, et al. (2008)[21] | 11 | | 90.9% (10/11) |
| Pooled total | 422 | | 90.3% (93/103)  90.2% - 90.9% |

***Notes:*** (1) ALA (aminolevulinic acid), APC (argon plasma coagulation), EMR (endoscopic mucosal resection), HGD (high grade dysplasia), HpD (hematoporphyrin derivative), MPEC (multipolar electrocoagulation), mTHPC (meta-tetrahydroxyphenylchlorin), PDT (photodynamic therapy), RFA (radiofrequency ablation), * * - (not available)
